# Synthesis and Biological Studies of Benzo[*b*]furan Derivatives: A Review from 2011 to 2022

**DOI:** 10.3390/ph16091265

**Published:** 2023-09-06

**Authors:** Lizeth Arce-Ramos, Juan-Carlos Castillo, Diana Becerra

**Affiliations:** Escuela de Ciencias Químicas, Universidad Pedagógica y Tecnológica de Colombia, Avenida Central del Norte 39-115, Tunja 150003, Colombia; lizeth.arce@uptc.edu.co

**Keywords:** benzo[*b*]furans, benzofurans, anticancer, antibacterial, antifungal, medicinal chemistry, drug discovery

## Abstract

The importance of the benzo[*b*]furan motif becomes evident in the remarkable results of numerous biological investigations, establishing its potential as a robust therapeutic option. This review presents an overview of the synthesis of and exhaustive biological studies conducted on benzo[*b*]furan derivatives from 2011 to 2022, accentuating their exceptional promise as anticancer, antibacterial, and antifungal agents. Initially, the discussion focuses on chemical synthesis, molecular docking simulations, and both in vitro and in vivo studies. Additionally, we provide an analysis of the intricate interplay between structure and activity, thereby facilitating comparisons and profoundly emphasizing the applications of the benzo[*b*]furan motif within the realms of drug discovery and medicinal chemistry.

## 1. Introduction

Heterocyclic chemistry plays a pivotal role in both chemical and life sciences, serving as a focal point for extensive global research. This branch of organic chemistry is dedicated to developing innovative molecules through the application of numerous synthetic protocols [1,2,3]. Heterocyclic compounds are widely distributed in naturally occurring and synthetic molecules, showcasing a broad range of physiological and pharmacological properties [4,5,6,7]. This diversity makes them particularly intriguing for applications in medicinal chemistry, material sciences, drug discovery, and the agrochemical and pharmaceutical industries [8,9,10,11]. Specifically, an analysis of the U.S. FDA-approved drug database revealed that around 60% of the top-selling drugs contain at least one heterocyclic nucleus [4]. These outstanding results can be attributed to the diverse intermolecular interactions between heterocycles and enzymes, involving hydrogen bonding, π-stacking, metal coordination bonds, and van der Waals and hydrophobic forces, along with their varied ring sizes, which enable a wide range of shapes to match the diverse enzyme binding pockets [12]. Moreover, drugs incorporating heterocycles exhibit improved solubility and the ability to facilitate salt formation, which are crucial factors in enhancing oral absorption and overall bioavailability [12]. Additionally, recent developments in synthetic methodologies targeting functionalized heterocycles play a key role in medicinal chemistry and drug discovery, effectively expanding the drug-like chemical space [1,13,14,15]. The establishment of reliable synthetic pathways for large-scale production further expedites drug development. Notably, the introduction of inventive heterocyclic syntheses, incorporating diverse bond-forming strategies, profoundly influences the pharmaceutical industry [1,13,14,15].

**Figure 1:**
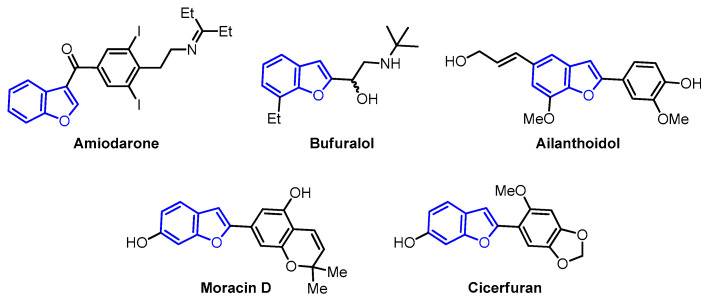
Bioactive benzo[*b*]furan derivatives.

In this context, oxygen-containing heterocycles stand out due to their broad spectrum of biological and pharmacological activities. Their significance stems from their structural similarities with a variety of well-established natural and synthetic compounds [16]. The inherent importance of oxygen-containing heterocycles as therapeutic agents resides in their distinctive structural characteristics, closely resembling those present in biologically active compounds, like ribose derivatives [17,18]. In these compounds, the prevalence presence of oxygen atoms contributes to polar interactions that foster stabilization within the active site [17,18]. Among these compounds, the benzo[*b*]furan scaffold, positioned within the domain of oxa-heterocycles has recently garnered significant attention. Its distinct physiological and chemotherapeutic properties are accentuated, further accompanied by its widespread prevalence in the natural realm. Some of the most prominent benzo[*b*]furan derivatives exhibit remarkable pharmaceutical applications, such as Amiodarone, used to treat life-threatening ventricular arrhythmias [19], and Bufuralol, employed as a nonselective *β*-adrenoceptor antagonist, which can lead to potential complications like hepatic toxicity or adverse drug interactions (Figure 1) [20]. Recent studies have shown that Ailanthoidol exhibits antitumor potential by suppressing TGF-*β*1-promoted HepG2 hepatoblastoma cell progression [21]. On the other hand, benzo[*b*]furan derivatives are widely distributed in various plant families, including Asteraceae, Fabaceae, and Moraceae [22,23]. For instance, Moracin D, isolated from *Morus alba*, exhibited anti-inflammatory and antioxidant activities, as well as induced apoptotic effects in prostate and breast cancer cells (Figure 1) [24,25]. Furthermore, Cicerfuran, isolated from the roots of chickpea (*Cicer* spp.), demonstrated antibacterial and antifungal activities [26]. The myriad applications in medicinal chemistry, biomedical science, and drug discovery have spurred both academia and the pharmaceutical industry to develop novel, efficient, and straightforward synthetic protocols for preparing a wide array of structurally diverse benzo[*b*]furan derivatives [23,27,28,29,30,31].

**Figure 2:**
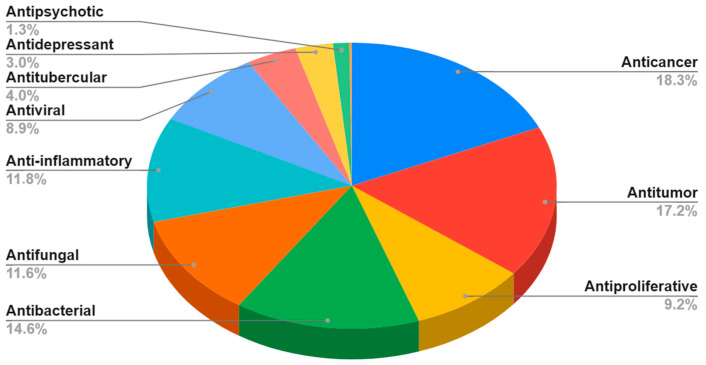
Bibliometric analysis: percentage distribution of articles across various biological activities of benzo[*b*]furan derivatives from 2011 to 2022 (data obtained from the Scopus database using the keywords: “benzo[*b*]furan” and each one activity).

Surprisingly, an exploration of the Scopus database covering the years from 2011 to 2022, encompassing all fields with the keywords “benzo[*b*]furan” and “biological activity,” has revealed a total of 7482 documents. Among these, 5431 are categorized as articles, while 1451 are identified as reviews. Notably, the array of published articles showcases a diverse spectrum of keywords, spanning a wide range of activities, including anticancer, antitumor, antiproliferative, antibacterial, antifungal, anti-inflammatory, antiviral, antitubercular, antidepressant, antipsychotic, α7 nAChR agonist, and antiosteoporosis activities (Figure 2). The data related to the last two activities are somewhat limited and may not be readily distinguishable on a graphic. Within this broad spectrum of activities, it is important to emphasize that articles related to cancer (anticancer, antitumor, and antiproliferative), as well as those focused on antimicrobial activity (antibacterial and antifungal), collectively constitute approximately 70% of the observed activities (Figure 2).

The most representative articles from these activities were selected and grouped under the headings of anticancer, antibacterial, and antifungal activities. In doing so, a focus was primarily placed on aromatic benzo[*b*]furans, excluding related search terms like furans and furanones. This approach resulted in a collection of 36 articles for the current review, of which 27 pertain to cancer (anticancer, antitumor, and antiproliferative) and 9 involve antimicrobial activity (antibacterial and antifungal). The articles chosen for this review offer insights into various aspects, including chemical synthesis, molecular docking discussions involving enzyme–substrate complexes, in vitro and in vivo studies, and diverse analytical techniques, such as flow cytometry, Western blotting, and confocal microscopy. These methodologies elucidate how the compounds impact the inhibition of the cell cycle, tumor regression, reduction in colony-forming units, and microbial growth. This broadens the scope for future investigations in the field of benzo[*b*]furan derivatives, encompassing chemical synthesis and the evaluation of anticancer and antimicrobial activities.

## 2. Synthesis of Bioactive Benzo[*b*]furan Derivatives

### 2.1. Anticancer Activity

**Scheme 1:**
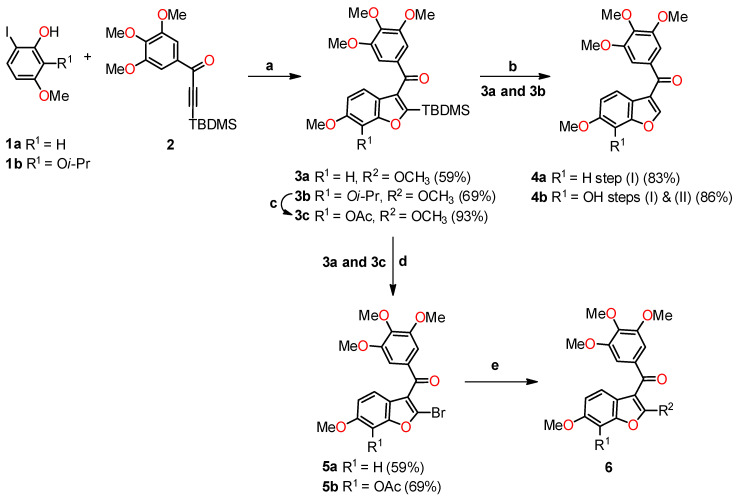
Modified Larock coupling synthesis. Reagents and conditions: (**a**) Pd(OAc)_2_, Na_2_CO_3_, DMF; (**b**) TBAF, THF, AlCl_3_, CH_2_Cl_2_; (**c**) AlCl_3_, CH_2_Cl_2_, Ac_2_O, Pyridine; (**d**) Br_2_, 1,2-dichloroethane; (**e**) coupling with palladium or nucleophilic displacement.

Benzo[b]furan derivatives have demonstrated a fascinating array of biological and pharmaceutical activities, including antitumor properties. For instance, Flynn et al. described the discovery of 7-hydroxy-6-methoxy-2-methyl-3-(3,4,5-trimethoxybenzoyl)benzo[b]furan (BNC105, **6a**, R^1^ = OH, R^2^ = Me), a potent and selective antiproliferative agent. They achieved the synthesis of various derivatives, many of which were obtained through a modified Larock-type coupling between o-iodophenol **1a**,**b** and 3-silyl-1-arylpropinone **2**, yielding 2-silylbenzo[b]furans **3a**,**b** in 59% and 69% yields, respectively (Scheme 1) [32,33]. Subsequently, silanes **3a** (R^1^ = H) and **3b** (R^1^ = Oi-Pr) underwent treatment with TBAF in methanol to remove the silyl groups. For compound **3b**, an additional reaction with AlCl_3_ was performed to eliminate the isopropyl group, resulting in the formation of compounds **4a** (R^1^ = H) and **4b** (R^1^ = OAc) with yields of 83% and 86%, respectively. On the other hand, compound **3a** underwent bromodesilylation with a 59% yield, producing 2-bromobenzo[b]furan **5a** (R^1^ = H). To avoid competitive bromination of the C–4 position of benzo[b]furan during the bromodesilylation process of **3b**, the isopropyl group was first exchanged for an acetyl group, yielding compound **3c**. Subsequent bromodesilylation of **3c** resulted in the formation of compound **5b** with a 69% yield. Additionally, the brominated derivatives **5a** and **5b** exhibited versatile functionality allowing bromine replacement through palladium coupling or nucleophilic displacement, leading to the formation of analog series **6**, which includes heterocyclic, carbocyclic, and alicyclic analogs at C–2 of benzo[b]furan (Table 1). One striking example of the versatility of the brominated derivatives is observed in the synthesis of the biologically significant compound **6a** (R^1^ = OH, R^2^ = Me), which was obtained via a Negishi reaction, coupling the derivative **5b** with methylzinc bromide using palladium, achieving an impressive 93% yield.

**Figure 3:**
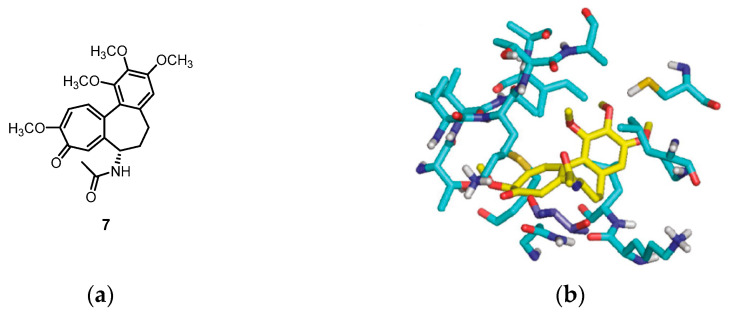

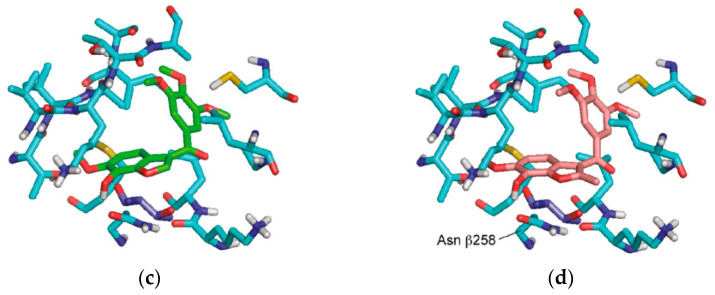
The molecular docking study utilized the X-ray crystal structure of the bovine *α*,*β*-tubulin dimer complexed with podophyllotoxin (PDB ID code: 1SA1). The benzo[*b*]furans were docked at the colchicine site using Glide. (**a**) The structure of colchicine **7**; (**b**) comparison with the orientation of colchicine **7** bound to the crystal; and (**c**,**d**) the docked orientations of **4b** and **6a**, respectively. The *β*-subunit of tubulin is represented by carbon atoms in light blue, and the Thr residue R179 is depicted in dark blue. Reprinted (adapted) with permission from ref. [32]. Copyright American Chemical Society, 2023.

A molecular docking simulation was performed to investigate the interactions of compounds **4b** and **6a** with the *α*,*β*-tubulin dimer complexed with podophyllotoxin (PDB ID code: 1SA1). During the docking study, the structures of compounds **4b** and **6a** were oriented, considering the structural similarities between these synthesized compounds and colchicine **7**. Specifically, the study focused on the interaction of colchicine with the *β*-tubulin subunit, where the 3,4,5-trimethoxyphenyl rings overlapped with similar rings in colchicine. Additionally, the C6–OMe and C7–OH substituents were examined for their interactions with the methoxy and carbonyl groups on the tropone ring of colchicine (Figure 3). The study showed the formation of a hydrogen bond between the C7–OH group of the benzo[*b*]furan (**4b** and **6a**) with the Asn *β*258 side chain, as well as the formation of a hydrogen bond with the amide nitrogen Val 181 in the adjacent subunit of *α*-tubulin. Furthermore, the orientation of the C–2 position of benzo[*b*]furan toward a gap between the *α*- and *β*-tubulin subunits allows it to harbor large substituents.

Another conducted study aimed to evaluate the effectiveness of benzo[*b*]furan **6a** on various human cancer cell lines using Combretastatin-A4 (CA-4) as a standard drug (Table 2). Interestingly, the results revealed that benzo[*b*]furan **6a** exhibited excellent selectivity against human aortic arterial endothelial cells (HAAECs), a characteristic not observed with CA-4. Furthermore, compound **6a** demonstrated significantly higher antiproliferative activity than CA-4, with up to a 10-fold increase in potency observed across many of the tested cell lines.

**Scheme 2:**
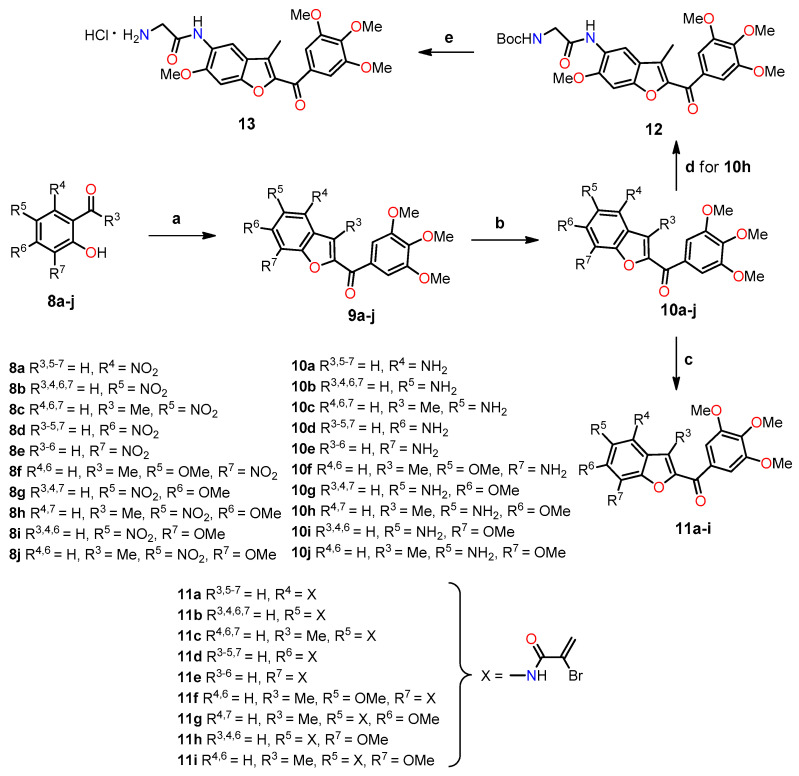
Synthesis of benzo[*b*]furans **9**–**13**. Reagents and conditions: (**a**) 2-bromo-1-(3′,4′,5′-trimethoxyphenyl)ethanone, K_2_CO_3_, acetone, reflux, 18 h; (**b**) Fe, HCl (37% in H_2_O), EtOH, reflux, 3 h, 42–79%; (**c**) *α*-bromoacrylic acid, EDCI, BtOH, DMF, r.t, 18 h, 44–66%; (**d**) *N*-Boc-glycine, EDCI, BtOH, DMF, r.t, 12 h, 83%; (**e**) 3M HCl in EtOAc, r.t, 3 h, 95%. Yields for compounds **9a**–**j** have not been reported by the authors.

On the other hand, Romagnoli et al. highlighted the significance of incorporating a 3,4,5-trimethoxybenzoyl group at the C–2 position of benzo[*b*]furan in determining the antiproliferative activity of benzofuran derivatives [34]. As depicted in Scheme 2, they synthesized a series of amino 2-(3′,4′,5′-trimethoxybenzoyl)-benzo[*b*]furan with good yields through three reaction steps: (1) a one-step cyclization reaction of nitrosalicylaldehydes or 2-hydroxyacetophenone **8** with 2-bromo-1-(3′,4′,5′-trimethoxyphenyl)ethanone and anhydrous potassium carbonate in acetone at reflux, yielding the nitro derivatives of 2-(3′,4′,5′-trimethoxybenzoyl)benzo[*b*]furanone **9a**–**j**; (2) the subsequent reduction of the nitro group using iron in a mixture of 37% HCl in water and ethanol at reflux, leading to the formation of the amino derivatives **10a**–**j**; and (3) the preparation of analogs **11a**–**i** in good yields through a substitution reaction between *α*-bromoacrylic acid and the amino benzo[*b*]furanone derivatives **10a**–**f** and **10h**–**j**, using an excess of two equivalents of EDCI and BtOH in dry DMF as a solvent. The glycine prodrug **13** was obtained in a 95% yield through the reaction between the amino derivative **10h** and *N*-Boc-glycine using EDCI and BtOH as coupling agents, with subsequent scission of the *N*-Boc protecting group with a solution of 3M HCl in ethyl acetate.

The antiproliferative activity of this series of derivatives, including amino 2-(3′,4′,5′-trimethoxybenzoyl)-benzo[*b*]furans **10a**–**j** and **11a**–**I**, was evaluated against various cancer cell lines, along with CA-4 as the standard drug (Table 3). Compound **10h** (R^4,7^ = H, R^3^ = Me, R^5^ = NH_2_, R^6^ = OMe) demonstrated the most promising results in the series, exhibiting significant growth inhibition against cancer cell lines L1210, FM3A/0, Molt4/C8, CEM/0, and HeLa, with IC_50_ values ranging from 16 to 24 nM. Notably, compound **10h** exhibited higher activity in the FM3A/0 cell line, with an IC_50_ value of 24 nM, compared to the standard drug CA-4 (IC_50_ = 42 nM). SAR information derived from the comparison of unsubstituted compounds **10b** (R^3,4,6,7^ = H, R^5^ = NH_2_), **10g** (R^3,4,7^ = H, R^5^ = NH_2_, R^6^ = OMe), and **10i** (R^3,4,6^ = H, R^5^ = NH_2_, R^7^ = OMe) vs. methyl derivatives **10c** (R^4,6,7^ = H, R^3^ = Me, R^5^ = NH_2_), **10h** (R^4,7^ = H, R^3^ = Me, R^5^ = NH_2_, R^6^ = OMe), and **10j** (R^4,6^ = H, R^3^ = Me, R^5^ = NH_2_, R^7^ = OMe) showed a significant increase in antiproliferative activity against cell lines by introducing the methyl group at the C–3 position of the benzofuran ring. Also, an increase in activity can be observed when comparing the methyl derivative **10c** with **10b**. When comparing the activities of compounds **10g** and **10h** with those of **10i** and **10j**, higher activity can be observed in compounds with methoxy groups at position C–6 rather than at position C–7 of the benzofuran ring. According to the results, compound **10h**, with a methyl group at the C–3 position and a methoxy group at the C–6 position, exhibited 2–4 times greater potency than the unsubstituted compound **10g** and 3–10 times higher activity than compound **10j**, which features a methoxy group at the C–7 position of the benzofuran ring. Changing the positions of the amino and methoxy groups from **10j** (R^4,6^ = H, R^3^ = Me, R^5^ = NH_2_, R^7^ = OMe) to **10f** (R^4,6^ = H, R^3^ = Me, R^5^ = OMe, R^7^ = NH_2_) resulted in a reduction in activity.

The unsubstituted *α*-bromoacryloylamide derivatives **11a** (R^3,5–7^ = H, R^4^ = X), **11b** (R^3,4,6,7^ = H, R^5^ = X), **11d** (R^3–5,7^ = H, R^6^ = X), and **11e** (R^3–6^ = H, R^7^ = X) exhibited antiproliferative potency that was 10–100 times greater than their amino counterparts, demonstrating the direct relationship between the presence of *α*-bromoacryloylamides and increased activity (Table 3). Shifting the *α*-bromoacryloylamide group from the C–4 at the 5 position to the C–6 at the 7 position led to decreased activity. Finally, there were no significant differences in antiproliferative activity on all cell lines observed between compound **10h** and its glycine hydrochloride prodrug **13**.

**Figure 4:**
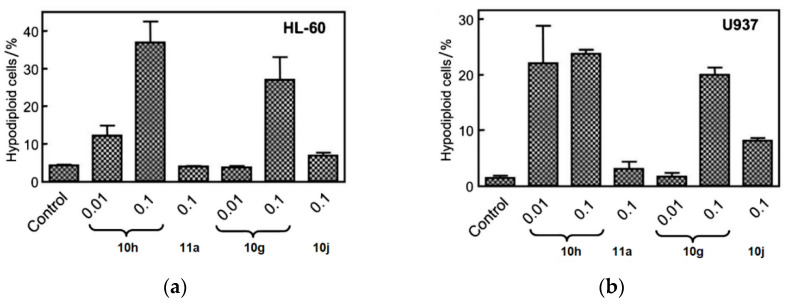
Compounds **10g**, **10h**, **10j**, and **11a** were put on approval against (**a**) HL-60 and (**b**) U937 cells for 24 h at indicated concentrations (in µM). Apoptosis was determined using flow cytometry. Values represent the mean ± SE of three independent experiments, each conducted in triplicate. Reproduced with permission from ref. [34]. Copyright John Wiley & Sons Inc., 2023.

To explore the potential correlation between antiproliferative activity and tubulin inhibition, the most active compounds, **10g**, **10h**, **10i**, **10j**, and **11a**, were evaluated in the inhibition of tubulin polymerization. Among these compounds, **10h** demonstrated the highest potency, displaying an IC_50_ value of 0.56 µM, which was two times higher than that of CA-4 (IC_50_ = 1.0 µM). Compounds **10g** and **10j** exhibited IC_50_ values of 1.4 and 1.6 µM, respectively, for tubulin polymerization, showing a marginal decrease in potency compared to CA-4. Compound **10i** exhibited approximately half the activity of **10j**. In contrast, compound **11a** did not induce any alteration in tubulin assembly, even at a concentration as high as 40 µM, suggesting that the mechanism of action of this *α*-bromoacryloylamide derivative does not involve interaction with tubulin. Subsequently, the effect of the selected compounds, **10g**, **10h**, **10j**, and **11a**, was assessed on the cell cycle of human myeloid leukemia cell lines HL-60 and U937 using flow cytometry. The cells were cultured for 24 h with a concentration of 100 nM for each compound, and the two most active compounds (**10g** and **10h**) were examined at a lower concentration of 10 nM. Figure 4 shows the fractions of hypodiploid cells in the sub-G1 peak of each compound studied, representing apoptotic cells. Compound **10j** showed a modest increase in apoptotic cells at 100 nM, while compounds **10g** and **10h** presented different effects on the cell cycle in the two cell lines. It was observed that compound **11a** had no effect on cell cycle distribution at 100 nM, in agreement with the previous results observed in tubulin inhibition. The significant increase in the sub-G1 peak in both cell lines with increasing concentrations of compounds **10g** and **10h** suggests that these compounds exert their growth inhibitory effect by inducing apoptosis.

**Figure 5:**
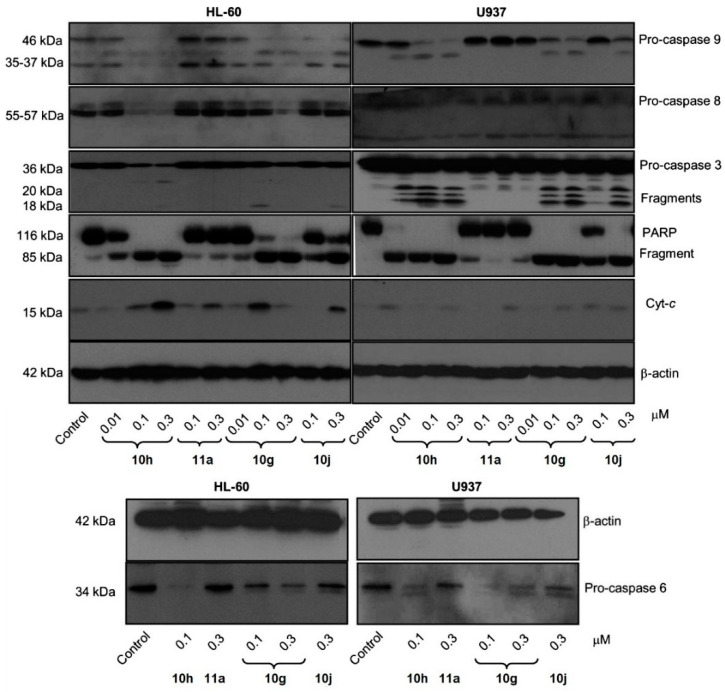
Relationship between caspases and the induction of apoptosis in two leukemia cell lines. Cells were incubated at different concentrations of compounds **10g**, **10h**, **10j**, or **11a**, and the cell lysates were analyzed by immunoblotting for cleavage of pro-caspases-9, -8, -6, and -3; poly(ADP-ribose) polymerase (PARP); and cytochrome c release. *β*-Actin served as the loading control. Reproduced with permission from ref. [34]. Copyright John Wiley & Sons Inc., 2023.

To further examine the apoptotic effects of compounds **10g**, **10h**, **10j**, and **11a**, proteolytic processing of caspases in HL-60 and U937 cells was observed by Western blot analysis (Figure 5). Compounds **10g**, **10h**, **10j**, and **11a** allowed the cleavage of inactive procaspase-9 to the active 37 kDa fragment, while lower concentrations of compounds **10g** and **10h** (0.3 µM) significantly promoted procaspase-8 hydrolysis. Furthermore, compounds **10g** and **10h** significantly led to the cleavage of inactive procaspases-3 and -6 in both cell lines (Figure 5). Additionally, the induction of poly(ADP-ribose)polymerase (PARP) cleavage showed that compounds **10j** and **11a** exhibited lower potency in inducing PARP cleavage compared to compounds **10g** and **10h**. The appearance of the fragment at 85 kDa coincided with caspase-3 activation, observed by a decrease in the proenzyme at 36 kDa and an increase in cleaved procaspase-3 levels at 20 and 18 kDa (Figure 5). Dose–response studies were performed, and cytosolic preparations were analyzed by immunoblotting to investigate whether apoptosis induced by compounds **10g**, **10h**, **10j**, and **11a** in HL-60 and U937 cells involved the release of cytochrome c from mitochondria into the cytosol during the apoptotic event. The results revealed a significant increase in the amount of cytochrome c in the cytosol of both cell lines at 15 kDa (Figure 5).

CA-4 and its analogs have been clinically termed vascular disruptors agents (VDAs) [35], so glycine prodrug **13** was tested to see its ability as a VDA in an in vivo model in rat breast cancer tumors, using the spatial frequency optical technique (SDFI). With 10 min of administration of compound **13** (30 mg kg^−1^), there is a rapid decrease in oxygen saturation in tumor tissues similar to that observed with CA-4, confirming that prodrug **13** causes vascular disruption in vivo.

**Scheme 3:**
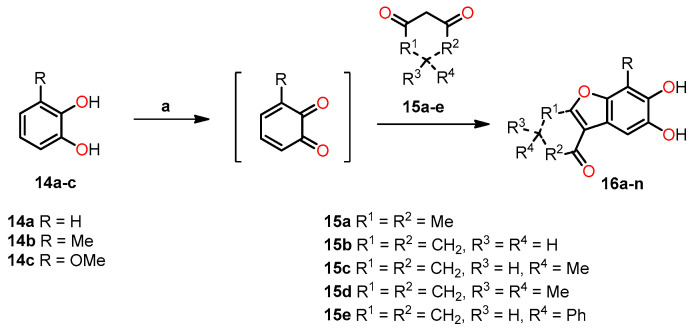
Synthesis of dihydroxylated 5,6-benzo[*b*]furans **16a**–**n**. Reagents and conditions: (**a**) Suberase, phosphate buffer, rt, pH 7.15.

Interestingly, Wellington et al. conducted the synthesis of a variety of dihydroxylated 5,6-benzo[*b*]furans **16** with catechol derivative **14** using a commercial laccase, Suberase^®^, under different reaction conditions [36]. All the synthesized compounds were evaluated for their anticancer properties. The synthetic protocol consisted of reacting an equivalent of catechol **14** with an equivalent of the 1,3-dicarbonyl compound **15** at room temperature using Suberase^®^ in an air-open vessel at pH 7.15 (Scheme 3). In method A, the reaction of catechol derivatives **14a**–**c** with 1,3-dicarbonyls compounds **15a**–**e** was performed at room temperature at pH 7.15 for 24 h. In method B, the reaction was conducted under similar conditions with an extended time of 44 h to investigate the potential enhancement in product yield with prolonged reaction time. In method C, a mixture of the 1,3-dicarbonyl compound and catechol, combined in a 4:1 ratio, was dissolved in DMF. Subsequently, the resulting mixture was left to react for 42 h. The results obtained from methods A–C are shown in Table 4. In particular, method A demonstrated the most favorable outcome of the three methods, exhibiting the highest yield of 98% for compound **16j** (Entry 15, Table 4). On the other hand, method B afforded compound **16k** in a 77% yield (Entry 18, Table 4), while method C yielded 71% for compound **16g** (Entry 11, Table 4). It is worth noting that the reaction time in Method B had minimal impact on the yield, whereas the presence of DMF in method C may have potentially deactivated the laccase, Suberase^®^, leading to lower yields.

Anticancer studies were performed on various types of cancer, including renal (TK10), melanoma (UACC62), breast (MCF7), and cervical (HeLa), using a sulforhodamine B (SRB) assay to determine the growth inhibitory effects of these compounds. Notably, the 5,6-dihydroxylated benzo[*b*]furans **16e**, **16g**, **16h**, **16k**, **16m** and **16n** exhibited potent cytotoxic effects against the melanoma cell line (UAC62), with GI_50_ values ranging from 0.77 to 9.76 µM. Among these compounds, **16h** (R = OMe, R^1^ = R^2^ = CH_2_, R^3^ = H, R^4^ = Me) and **16n** (R = OMe, R^1^ = R^2^ = CH_2_, R^3^ = H, R^4^ = Ph) showed better activity than the standard drug Etoposide (GI_50_ = 0.89 µM). Moreover, compound **16n** showed potent activity (GI_50_ = 9.73 µM) against the renal cancer cell line (TK10), while both **16h** and **16n** demonstrated strong activity against the breast cancer cell line (MCF7), with GI_50_ values of 8.79 and 9.30 µM, respectively.

**Scheme 4:**
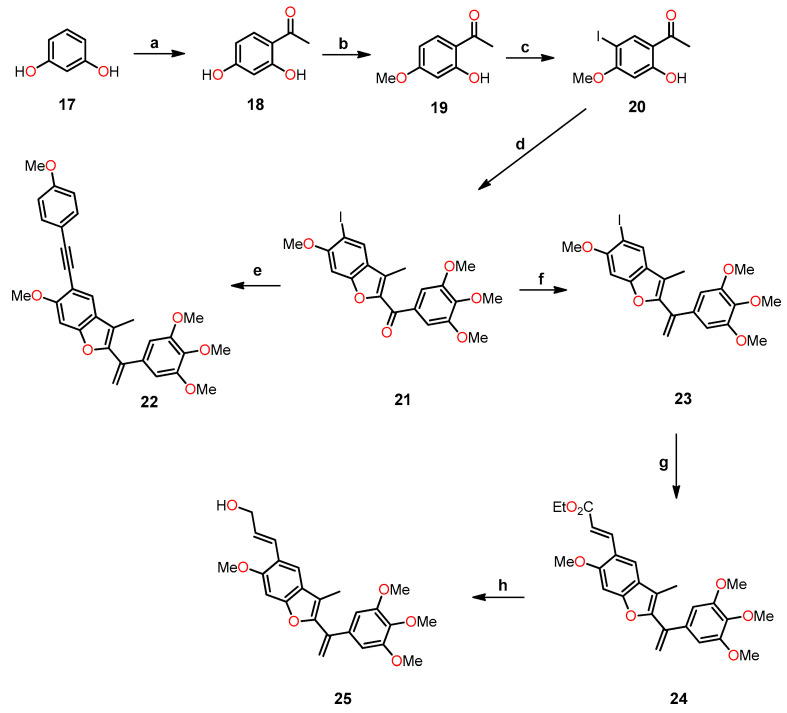
Synthesis of benzo[*b*]furan derivatives **22** and **25**, and the reaction conditions: (**a**) anhydrous ZnCl_2_, CH_3_COOH, reflux, 12 h, 73%; (**b**) K_2_CO_3_, CH_3_I, acetone, reflux, 18 h, 85%; (**c**) I_2_, AgNO_3_ (cat.), Ph_3_PCH_3_Br, THF, 0 °C, 50–70%; (**d**) 2-bromo-1-(3,4,5-trimethoxyphenyl)ethenone, K_2_CO_3_, acetone, reflux, 12 h, 75%; (**e**) substitution alkynes, PdCl_2_(PPh_3_)_3_, CuI, Et_3_N, THF, 60 °C, 14 h, 62–65%; (**f**) LiHMDS, Ph_3_PCH_3_Br, THF, 0 °C, 50–70%; (**g**) ethyl acrylate, PdCl_2_(Ph_3_P)_2_, Et_3_N, DMF, 60 °C, 24 h, 65%; (**h**) DIBAL, CH_2_Cl_2_, 0 °C, 1 h, 60%.

In 2013, Kamal et al. synthesized a series of benzo[*b*]furans with a modification at position 5 of the benzene ring by introducing C-linked substituents to generate 2-(3′,4′,5′-trimethoxybenzoyl)benzo[*b*]furan derivatives [37]. The most biologically interesting benzo[*b*]furan derivatives, **22** and **25**, were synthesized through a sequence of reactions depicted in Scheme 4, which included (a) the acylation of **17** to yield product **18**; (b) methylation using methyl iodide and potassium carbonate, resulting in **19**; (c) iodination with iodine and silver nitrate in a catalytic amount to produce **20**; (d) cyclization with 2-bromo-1-(3,4,5-trimethoxyphenyl)ethanone and potassium carbonate in acetone to furnish the benzofuran derivative **21**; (e) Sonogashira coupling reaction to obtain **22**; (f) Wittig reaction with the ylide generated from methyltriphenylphosphonium bromide in the presence of LiHMDS, leading to **23**; (g) Heck coupling reaction with ethyl acrylate to yield the ethyl cinnamate derivative **24**; and (h) ester reduction with DIBAL resulting in the formation of (*E*)-allyl alcohol **25**.

A study was performed to assess the cytotoxicity of benzofuran analogs against ME-180, A549, ACHNs, HT-29, and B-16 cell lines using a 3-(4,5-dimethylthiazol-2-yl)-2,5-diphenyltetrazolium bromide (MTT) assay and CA-4 as the standard drug (Table 5). The results from compound **22**, which contains a 4-MeO-phenylacetylene group, showed good activity, with IC_50_ values in the range of 0.08–1.14 µM against all evaluated cell lines, as shown in Table 5. However, derivative **22** was two times less active against A549 and significantly less active against all four cell lines compared to CA-4. On the other hand, analog **25** exhibited the effect of the alkenyl substituent at position 5 of benzofuran on cytotoxicity, showing higher potency compared to compound **22** against ME-180, A549, ACHN, and B-16 cancer cell lines, with IC_50_ values ranging from 0.06 to 0.17 µM. These values were comparable to those obtained with CA-4 against A549 and ACHN cancer lines, with IC_50_ values of 0.05 and 0.09 µM, respectively. Furthermore, compounds **22** and **25** inhibited tubulin polymerization by 37.9 and 65.4%, respectively, which is comparable to the 70.5% tubulin inhibition observed with CA-4 (Table 5).

**Figure 6:**
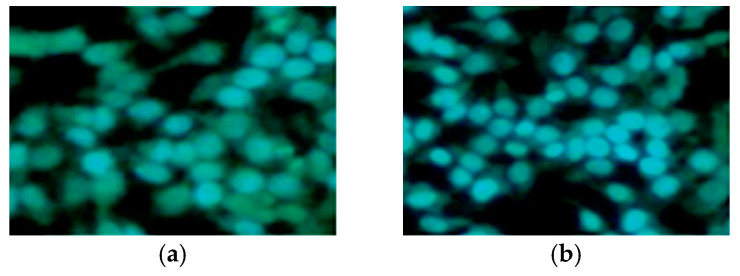

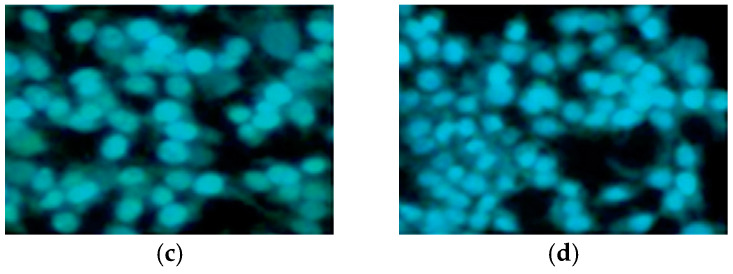
Hoechst staining in the A549 cell line. (**a**) A549 control cells; (**b**) CA-4 (50 nm); (**c**) compound **22** (50 nm); and (**d**) compound **25** (50 nm). Reproduced with permission from ref. [37]. Copyright John Wiley & Sons Inc., 2023.

Apoptotic studies were performed using various assays, including Hoechst staining assay, caspase-3 activation, DNA fragmentation analysis, and Western blot analysis. Specifically, a Hoechst staining assay was utilized to study the effects of compounds **22** and **25** on nuclear condensation. Remarkably, cells treated with these compounds showed a pronounced increase in nuclear condensation compared to untreated cells, strongly suggesting their potent ability to induce cell apoptosis (Figure 6). In addition, caspase-3 activation analysis was conducted on A549 cells, treating them with concentrations of 50 and 100 nM of compounds **22** and **25** and comparing them to CA-4 at 100 nM. The results indicated a significant increase in caspase-3 activation, ranging from 1.5- to 3-fold compared to the control experiment (50 and 100 nM), demonstrating the programmed apoptotic activity induced by compounds **22** and **25** in the A549 cells.

**Figure 7:**
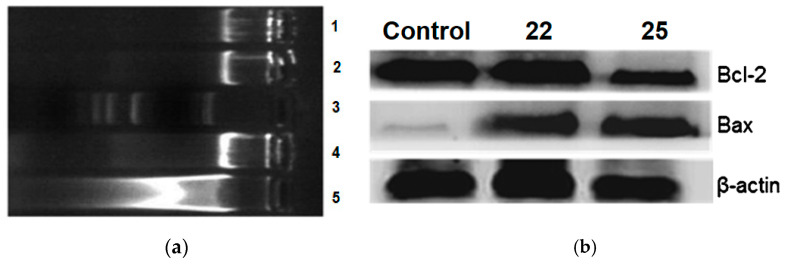
(**a**) DNA fragmentation analysis was performed on A549 lung cancer cells treated with compounds **22** and **25**. The gel electrophoresis results show lane 1: control (A549 cells), lane 2: CA-4 (50 nM), lane 3: marker (100 bp), lane 4: **22** (50 nM), and lane 5: **25** (50 nM). (**b**) The effect of compounds on Bcl-2 and Bax levels. A549 cells were treated with compounds **22** and **25** at 50 nM for 48 h. Cell lysates were subjected to Western blot analysis to determine the expression levels of Bcl-2 and Bax, using *β*-actin as the loading control. Reproduced with permission from ref. [37]. Copyright John Wiley & Sons Inc., 2023.

DNA fragmentation analysis was conducted by incubating A549 cells with a 50 nM concentration of compounds **22** and **25**. The results revealed a discrete staircase pattern after 48 h of treatment, indicative of significant fragmentation associated with cell death (Figure 7a). Additionally, Western blot analysis was realized on the same cancer cell line, treating it with the same concentration of compounds **22** and **25** as used in the DNA fragmentation analysis (Figure 7b). After 48 h of treatment, it was observed that the anti-apoptotic protein Bcl-2 was down-regulated, while the pro-apoptotic protein Bax was up-regulated. The results provide evidence that the induction of apoptosis by compounds **22** and **25** is associated with Bcl-2 down-regulation.

In a study conducted by Frías et al., an asymmetric synthesis of diheteroarylalkanes was presented. This synthesis involved a one-pot reaction using dienamine and Friedel–Crafts reactions between aldehyde **26** and indole **27**, catalyzed by the Hayashi–Jorgensen catalyst **28** (20 mol %). Various substituents at different positions on the aldehyde and indole were utilized during the reaction [38]. When starting, materials with electron-withdrawing groups (EWGs) or electron-donating groups (EDGs) were located at the *para* position to the oxygen atom, and products **29a**,**g**–**I** showed good yields and enantioselectivity ranging from 93% to 97% toward the (*S*)-enantiomer (Table 6). However, substrates substituted at the *ortho* and *meta* positions also enabled the synthesis of products **29j** and **29k** without a decrease in the final enantioselectivity (*ee* = 93% and 94%, respectively). Indoles with bromo (**29b**) or methoxy (**29c**) substituents exhibited satisfactory yields and enantioselectivity (>95%). Methyl groups displayed good yield and enantioselectivity in products **29d** and **29e** (*ee* = 99% and 96%, respectively). Additionally, the introduction of the 1*H*-benzo[*g*]indole group resulted in the desired aldehyde **29f** with excellent enantioselectivity (*ee* = 98%).

**Scheme 5:**
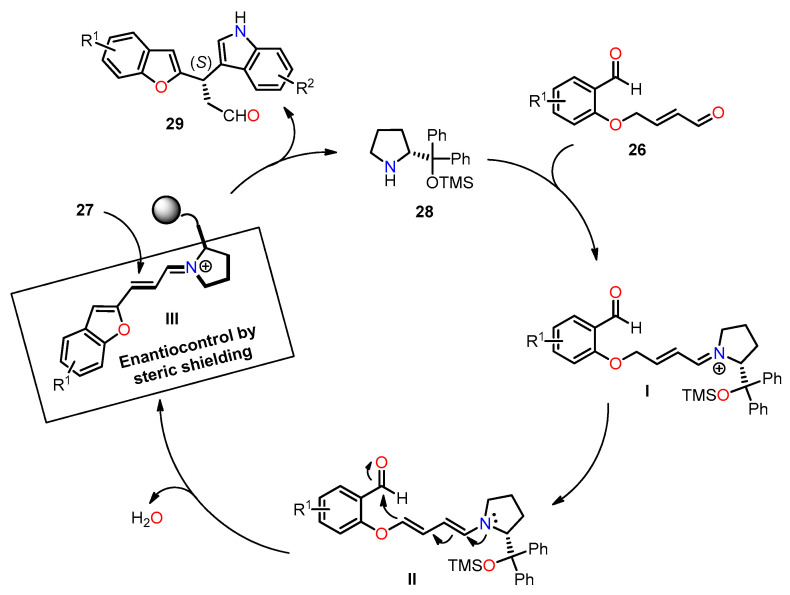
Plausible reaction mechanism for the synthesis of diheteroalkane **29**.

The enantioselectivity of compound **29** is explained through the proposed reaction mechanism depicted in Scheme 5. It begins with the condensation reaction between aldehyde **26** and organocatalyst **28** to form iminium ion **I**. Then, the isomerization of **I** gives dienamine intermediate **II**, which undergoes intramolecular condensation/dehydration sequence to afford iminium ion **III**. At this pivotal stage, indole **27** undergoes an attack on intermediate **III**, leading to the formation of product **29** with remarkable enantioselectivity. This precise enantiocontrol is facilitated by the steric shielding offered by the bulkier group (CPhPhOTMS) present in the organocatalyst.

**Figure 8:**
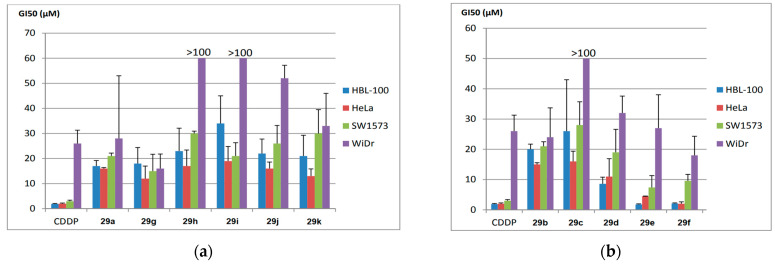
Antiproliferative activity (GI_50_, µM) in various human solid tumor cell lines, including HBL-100 (breast), HeLa (cervix), SW1573 (non-small-cell lung), and WiDr (colorectal). The compounds evaluated are presented in two groups: (**a**) **29a**, **29g**–**k**, and (**b**) **29b**–**f**. Reproduced with permission from ref. [38]. Copyright John Wiley & Sons Inc., 2023.

The synthesized products **29a**–**k** were evaluated for their antiproliferative activity against a panel of tumor cell lines, including HBL-100 (breast), HeLa (cervix), SW1573 (non-small-cell lung), and WiDr (colon), using the SRB assay. Figure 8 presents the GI_50_ values, comparing them with *cis*-platin as the standard drug. The results highlight the significance of the substituent on the aryl moiety of the benzofuran ring in influencing the antiproliferative activity of product **29**. The introduction of strong electron-withdrawing groups (EWGs) and electron-donating groups (EDGs) resulted in a decrease in activity for compounds **29h**, **29i**, and **29j** in WiDr cell lines. Notably, compounds **29a** (R^1^ = R^2^ = H) and **29g** (R^1^ = 5-Cl, R^2^ = H) exhibited GI_50_ values comparable to *cis*-platin in the WiDr cell line, with values of 28 and 16 µM for compounds **29a** and **29g**, respectively. However, the product of highest biological interest was **29f** (R^1^ = H, R^2^ = benzo[*g*]), which showed the most significant activity across all cell lines, achieving similar or even better potency than *cis*-platin (GI_50_ = 2–18 µM).

**Figure 9:**
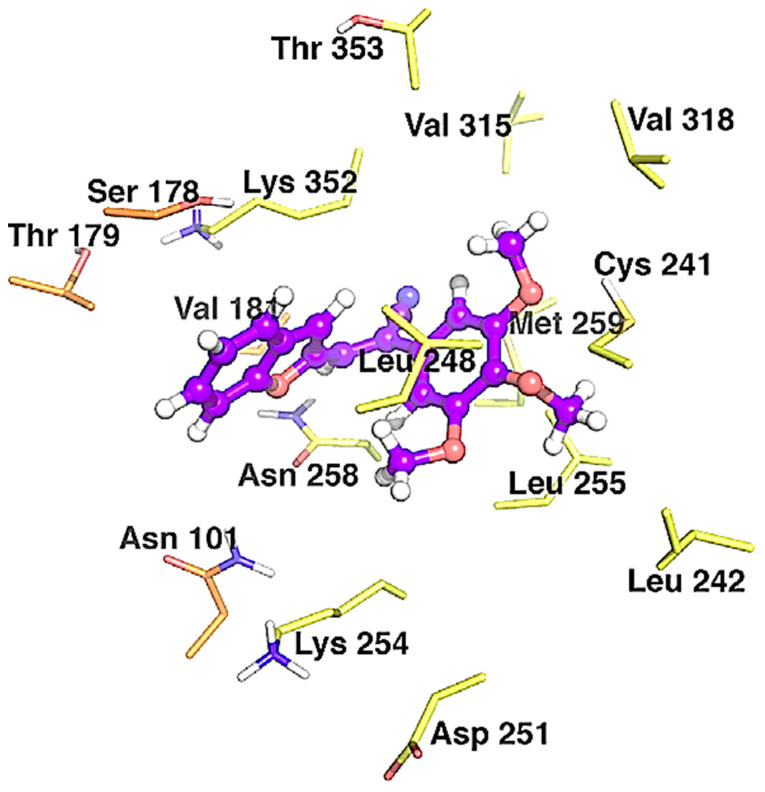
Binding modes between **32a** and α/β tubulin in complex with colchicine-DAMA (PDB ID code: 1SA0). The inhibitors are represented as purple ball-and-stick models, while the tubulin residues are shown as orange (*α*-tubulin) and yellow (*β*-tubulin) sticks. Reproduced with permission from ref. [39]. Copyright Elsevier Inc., 2023.

In a separate study, Penthala et al. synthesized a series of heterocyclic analogs, including indoles, benzofurans, and benzothiophenes, based on Combretastatin, and assessed their anticancer activity against a panel of 60 human cancer cell lines [39]. The benzo[*b*]furans were synthesized by condensing benzo[*b*]furanocarbaldehyde **30** (1.0 mol) with phenylacetonitrile **31** (1.1 mol) in a 5% sodium methoxide/methanol solution for 3–6 h, resulting in the successful formation of the desired product **32** (Table 7). The evaluation of anticancer studies focused on compounds **32a**, **32b**, and **32d** against 60 cancer cell lines. Compound **32a** exhibited the most favorable results, displaying GI_50_ values ranging from <0.01 to 73.4 µM across all 60 cell lines. It effectively inhibited the growth of 70% of the evaluated cancer cell lines, with a remarkable GI_50_ value < 0.01 µM in almost all cases. On the other hand, substituting the 3,4,5-trimethoxyphenyl group in **32a** (R = 2-CHO, R^1^ = OMe, R^2^ = OMe) with the 3,4-dimethoxyphenyl group in **32b** (R = 2-CHO, R^1^ = H, R^2^ = OMe) resulted in reduced growth inhibition against 54% of the cancer cells, exhibiting GI_50_ values ranging from 0.229 to 0.996 µM. Furthermore, compound **32b** exhibited potent anti-proliferative activity in MDA-MB-435 melanoma cells, exhibiting a remarkable GI_50_ value of 0.229 µM. Similarly, compound **32d** (R = 3-CHO, R^1^ = OMe, R^2^ = OMe) displayed significant antiproliferative inhibition, with GI_50_ values ranging from 0.237 to 19.1 µM, and effectively inhibited 52% of the evaluated cell lines with a GI_50_ value < 1 µM. In addition, the evaluation of anti-leukemia activity against the MV4–11 cell line was performed for compounds **32a**–**d** (Table 7), demonstrating that **32a** emerged as the most active compound among the evaluated benzo[*b*]furans in the leukemia cell line. Later, a molecular docking simulation was performed to investigate the interactions of compound **32a** with *α*/*β* tubulin in complex with colchicine-DAMA (PDB ID code: 1SA0). The simulation revealed a hydrophobic interaction at the *α*–*β* interface, where colchicine binds, and stability was observed through van der Waals interactions with Asn101, Ser178, Thr179, and Val181 in *α*-tubulin, as well as Asn258 and Lys352 in *β*-tubulin (Figure 9). The calculated free energy value for these interactions was −7.74 kcal/mol.

**Scheme 6:**
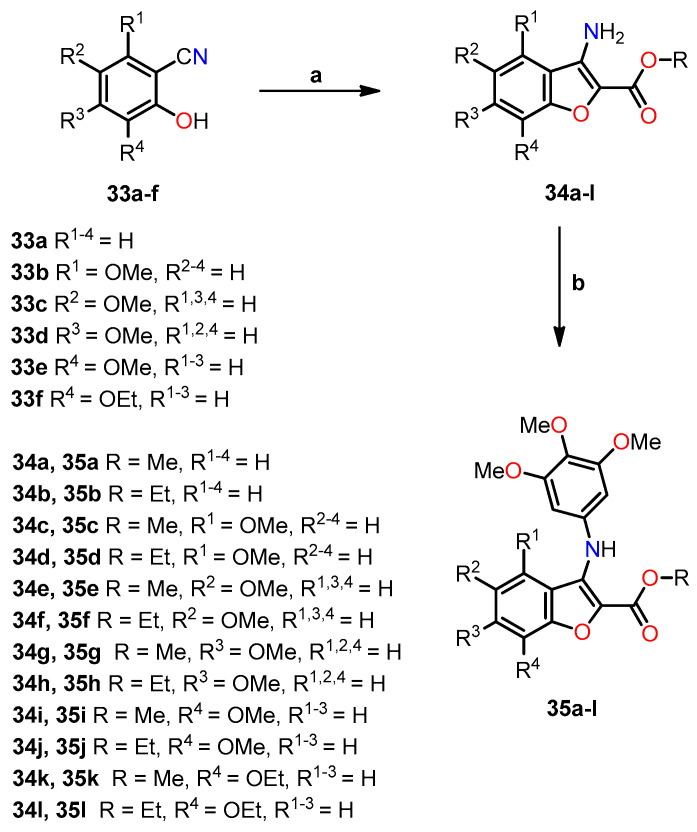
Synthesis of benzo[*b*]furan analogs **35a**–**l** and the reactions conditions: (**a**) BrCH_2_CO_2_CH_3_ or BrCH_2_CO_2_C_2_H_5_, K_2_CO_3_, DMF, 60 °C for 4 h, then reflux for 8 h, 52–78%; (**b**) 1-bromo-3,4,5-trimethoxybenzene, Pd(OAc)_2_, BINAP, Cs_2_CO_3_, PhMe, 100 °C, 16 h, 52–78%. Reprinted (adapted) with permission from ref. [40]. Copyright American Chemical Society, 2023.

In the same year, Romagnoli et al. synthesized a series of compounds known as 3-(3′,4′,5′-trimethoxyanilino)benzo[*b*]furan, wherein a 2-methoxy/ethoxycarbonyl group was combined with either no substituent or a methoxy group at each position of the benzene ring [40]. The synthesis of compounds **35a**–**l** involved a two-step reaction process (Scheme 6). In the first step, 2-hydroxybenzonitrile derivatives **33a**–**f** were condensed with methyl or ethyl bromoacetate and K_2_CO_3_ in DMF, leading to the formation of 3-aminobenzo[*b*]furan analogs **34a**–**l** in high yields through a one-pot tandem cyclization method. Subsequently, compounds **35a**–**l** were synthesized via palladium-catalyzed C-N Buchwald–Hartwig cross-coupling between the deactivated 3-aminobenzo[*b*]furans **34a**–**l** and 1-bromo-3,4,5-trimethoxybenzene in toluene at 100 °C, utilizing Pd(OAc)_2_, *rac*-BINAP, and Cs_2_CO_3_ as the catalyst, ligand, and base, respectively.

The in vitro antiproliferative activity was evaluated against seven cell lines, and the corresponding results are presented in Table 8. The findings revealed a notable correlation between the presence and position of the methoxy substituent on the benzene moiety of the benzo[*b*]furan system. Among the series of 2-alkoxycarbonyl derivatives, the highest activity was observed when the methoxy group was located at the C–6 position, as exemplified by compounds **35g** (R = Me, R^3^ = OMe, R^1,2,4^ = H) and **35h** (R = Et, R^3^ = OMe, R^1,2,4^ = H), exhibiting IC_50_ values ranging from 0.3 to 27 nM for **35g** and from 13 to 100 nM for **35h**. On the contrary, compounds **35c** (R = Me, R^1^ = OMe, R^2−4^ = H) and **35d** (R = Et, R^1^ = OMe, R^2−4^ = H) displayed the lowest activity when the methoxy group was situated at the C–4 position, with IC_50_ values exceeding 10 µM. Furthermore, the methoxycarbonyl group demonstrated superior efficacy compared to the ethoxycarbonyl substituent in all cell lines, except for MCF-7 cells, which exhibited equal sensitivity to both compounds. Notably, compounds **35i** (R = Me, R^4^ = OMe, R^1−3^ = H) (average IC_50_ = 370 nM) and **35j** (R = Et, R^4^ = OMe, R^1−3^ = H) (average IC_50_ = 670 nM), featuring a methoxy C–7 substituent, displayed higher activity compared to **35e** (R = Me, R^2^ = OMe, R^1,3,4^ = H) (average IC_50_ = 1.500 nM) and **35f** (R = Et, R^2^ = OMe, R^1,3,4^ = H) (average IC_50_ = 2.900 nM), which possessed a methoxy C–5 substituent. These compounds also demonstrated remarkable activity against RS 4;11 cells, with IC_50_ values of 39 nM for **35e** and 1 nM for **35i** (R = Me, R^4^ = OMe, R^1−3^ = H). Additionally, in Jurkat cells, they displayed an IC_50_ value of 30 nM for **35i**. In contrast, compounds **35a** (R = Me, R^1−4^ = H) and **35b** (R = Et, R^1−4^ = H) exhibited IC_50_ values of 3.300 and 2.600 nM, respectively. The absence of a methoxy substituent led to lower activity, highlighting the significant enhancement achieved by including a methoxy substituent at C–5. Furthermore, the substitution with ethoxycarbonyl at C–7 resulted in notably lower potency when compared to the substitution with C–7-methoxy (**35i**–**l**).

Compounds **35e** and **35g**–**j**, along with CA-4, were investigated to determine their inhibitory effects on tubulin polymerization and colchicine binding to tubulin. The aim was to gain insights into their mechanisms of antiproliferative action, particularly their interaction with tubulin microtubules (Table 9). The results revealed that compound **35g** exhibited the highest potency among the tested compounds, with an IC_50_ of 1.1 µM, comparable to that of CA-4. Meanwhile, compound **35h** demonstrated slightly lower activity compared to CA-4. Compounds **35e**, **35i**, and **35j** showed 6–7 times lower potency than CA-4, with IC_50_ values of 7.5, 7.6, and 6.4 µM, respectively. Regarding colchicine binding studies, outcomes were observed exclusively for compounds **35g** and **35h**, exhibiting inhibition percentages of 83% and 74%, respectively, which are comparable to the 99% inhibition observed with CA-A. The findings underscore the intricate interplay between the inhibition of tubulin polymerization and the hindrance of colchicine binding, shedding light on their potential synergistic effects in influencing antiproliferative pathways.

**Figure 10:**
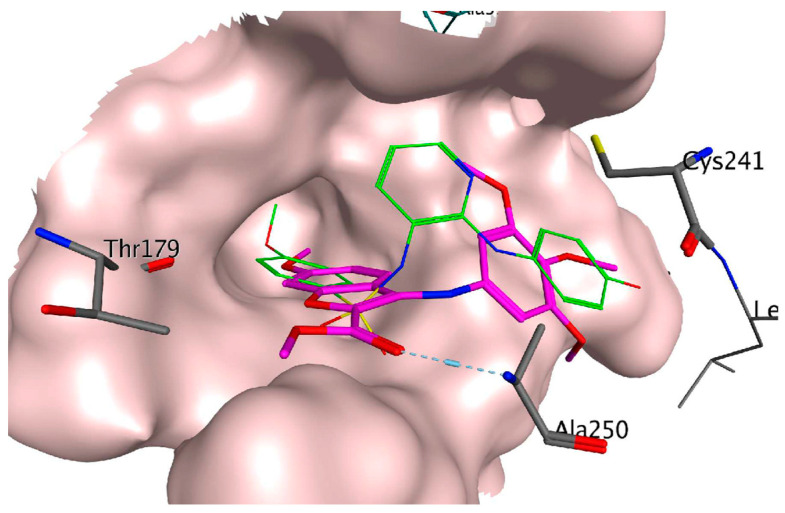
Binding modes of compound **35g** (represented in magenta) in the colchicine site of tubulin (PDB ID code: 3HKC). The co-crystallized ligand *N*-[2-[(4-hydroxyphenyl)amino]-3-pyridinyl]-4-methoxybenzenesulfonamide (PDB ID code: ABT751) is represented in green. Reprinted (adapted) with permission from ref. [40]. Copyright American Chemical Society, 2023.

A molecular docking simulation was performed to investigate the interactions of compound **35g** with the colchicine site of tubulin (PDB ID code: 3HKC) (Figure 10). This revealed that the trimethoxyphenyl ring of **35g** resides near Cys241. Moreover, a potential hydrogen bond interaction was observed between the ester moiety and Ala250, consistent with other colchicine site agents. These findings underscore the potential impact of substitutions at C–4, C–5, and C–7 on the antiproliferative activity of the compounds.

**Figure 11:**
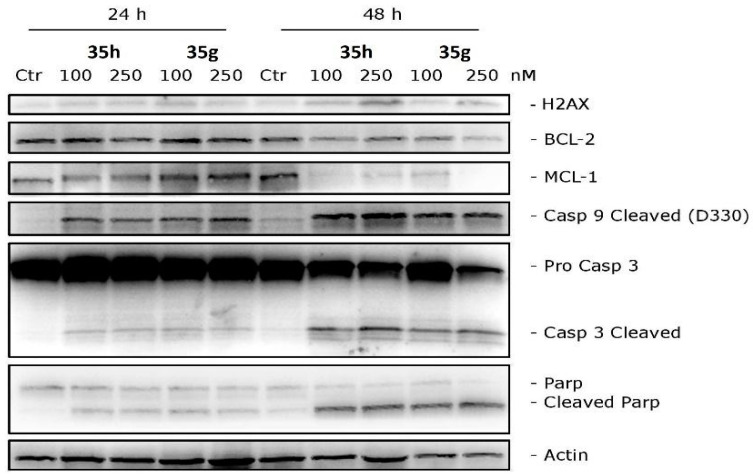
Western blot analysis was conducted on HeLa cells treated with different concentrations of compounds **35g** or **35h** to investigate their effects on H2AX, Bcl-2, Mcl-1, caspase-3, cleaved caspase-9, and PARP. *β*-Actin was used as the loading control. Reprinted (adapted) with permission from ref. [40]. Copyright American Chemical Society, 2023.

Conducting Western blot studies, we aimed to explore the potential of compounds **35h** and **35g** in triggering apoptosis via the activation of caspase-3 and caspase-9, crucial components of the mitochondrial apoptotic pathway. Upon exposing HeLa cells to these compounds, we observed a concentration- and time-dependent activation of caspases, as depicted in Figure 11. Moreover, both in vitro and in vivo revealed the activation of poly(ADP-ribose) polymerase (PARP), a major substrate targeted by caspase-3. In addition to these findings, we carefully examined the role of Bcl-2 and Mcl-1 proteins, well known for their capacity to counteract pro-apoptotic proteins and preserve mitochondrial membrane potential. After 48 h of treatment with concentrations of 100 and 250 nM for both compounds, a decrease in Bcl-2 protein expression was observed, while Mcl-1 showed strong down-regulation. Interestingly, at 24 h, Mcl-1 expression increased for **35g** but not for **35h**. These results suggest that compounds **35g** and **35h** effectively down-regulate anti-apoptotic proteins.

**Figure 12:**
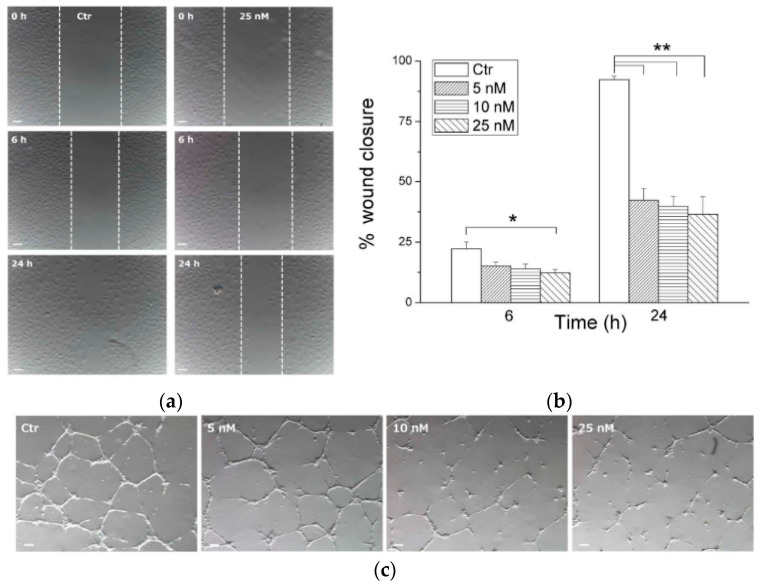
In vitro antivascular evaluation of compound **35g**. (**a**) Confluent HUVEC samples were prepared in a monolayer, and cells were treated with different concentrations of **35g**. The cells were photographed at various time intervals (magnification, 7×; bar, 100 μm). Dotted lines define areas lacking cells. (**b**) The graph shows the quantitative effect of compound **35g**, where gap closure was measured at specified time intervals to assess migration. Data are represented as mean of three independent experiments. * *p* < 0.05, ** *p* < 0.01 vs. control. (**c**) Inhibition of endothelial cell capillary tubule formation by compound **35g**. Representative images show preformed capillary tubules treated with increasing concentrations of **35g** for 1 or 3 h. Reprinted (adapted) with permission from ref. [40]. Copyright American Chemical Society, 2023.

To assess angiogenesis, the vascular properties of **35g** (the most potent within the series) were investigated in vitro using HUVEC endothelial cells. The endothelial cell motility and the ability of **35g** to disrupt tubular structures formed by HUVECs on Matrigel were investigated. As shown in Figure 12a,b, at a concentration of 25 nM, compound **35g** exhibited significant inhibition of cell motility within just 6 h of incubation. This inhibitory effect remained highly significant at all concentrations after 24 h of incubation. Moreover, in Figure 12c, it was observed that compound **35g** disrupted the network of HUVECs compared to the control after 1 h of incubation. Remarkably, after 3 h, all tested concentrations demonstrated significant disruption of the tubular-like structures.

**Figure 13:**
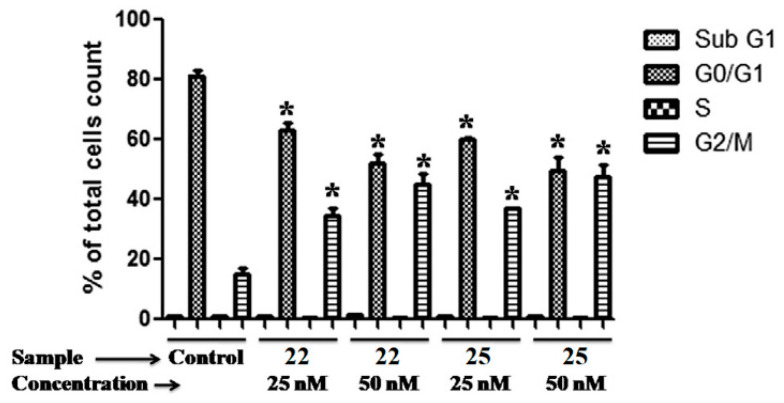
Cell cycle evaluation of compounds **22** and **25** in the MCF-7 cell line. The data are presented as the percentage of cell count at each induced cell cycle phase for each compound (* *p* < 0.05 vs. control). Reproduced with permission from ref. [41]. Copyright Elsevier Inc., 2023.

Expanding on the encouraging results regarding the antiproliferative and anticancer activity [37], Kamal et al. conducted more extensive investigations on benzo[*b*]furans **22** and **25** to explore their potential efficacy against breast cancer cell lines, specifically MCF-7 and MDA MB-231. These studies involved assessments of the cell cycle and the PI3K/Akt/mTOR signaling pathway, along with other complementary studies [41]. Table 10 presents the results obtained for the antiproliferative activity of compounds **22** and **25** against the mentioned cancer cell lines. Remarkably, **22** and **25** displayed significant activity, especially in the MCF-7 cell line, exhibiting IC_50_ values of 0.057 and 0.051 µM, respectively. Due to the MCF-7 cell line showing the highest anticancer activity among the tested cell lines, it was chosen for further analysis to investigate the correlation between cell growth inhibition and cell cycle arrest. In this study, MCF-7 cells were treated with the compounds **22** and **25** at concentrations of 25 nM and 50 nM for 48 h. The results revealed that these compounds induced G2/M cell cycle arrest compared to the untreated control cells. Specifically, at a concentration of 25 nM, compounds **22** and **25** caused a cell accumulation of 36.4% and 37.1%, respectively, in the G2/M phase. Moreover, at 50 nM, these percentages increased to 47.6% and 50.5%, respectively, in the same cell phase (Figure 13).

**Figure 14:**
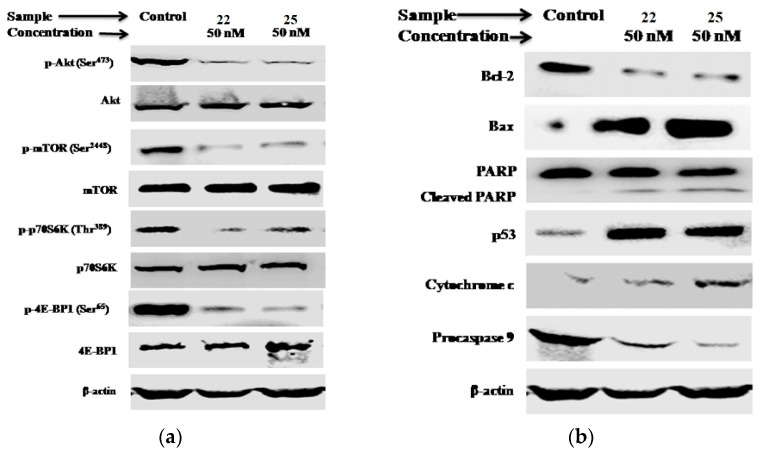
Analysis of the PI3K/Akt/mTOR pathway and apoptotic markers in MCF-7 cells treated with compounds **22** and **25** for 48 h at a concentration of 50 nM. The expression levels of (**a**) Akt, mTOR, p70S6K, and 4E-BP1, including their phosphorylated forms, as well as (**b**) cytochrome c, Bax, Bcl-2, p53, procaspase-9, and PARP, were measured. Protein expression levels were analyzed using Western blot analysis, with *β*-actin serving as the loading control in both cases. Reproduced with permission from ref. [41]. Copyright Elsevier Inc., 2023.

The PI3K/Akt/mTOR signaling pathway plays a crucial role in breast tumor cell growth. Thus, the impact of compounds **22** and **25** on this signaling pathway was investigated in MCF-7 cells. The results demonstrated effective suppression of p-Akt, p-mTOR, p-p70S6K, and p-4E-BP1 expression levels after 48 h of treatment with a concentration of 50 nM (Figure 14a). The findings strongly support the potent inhibitory activity of both compounds against the PI3K/Akt/mTOR pathway. Notably, given the involvement of this pathway in apoptosis regulation, the studies further unveiled that its inhibition resulted in the up-regulation of key apoptotic markers. These markers included the release of cytochrome c, up-regulation of p53, down-regulation of procaspase-9, cleavage of poly(ADP-ribose)polymerase (PARP), up-regulation of Bax, and down-regulation of Bcl-2 (Figure 14b). Collectively, these results firmly establish the inhibition of the PI3K/Akt/mTOR pathway as the primary mechanism underlying the induction of apoptosis in breast cancer cells by compounds **22** and **25**.

**Scheme 7:**
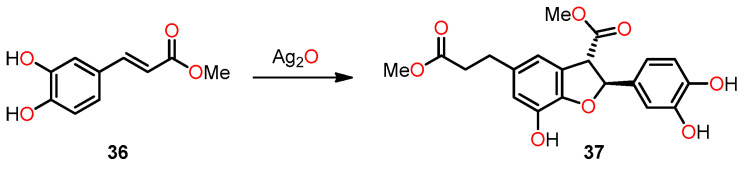
Synthesis of 2,3-dihydrobenzo[*b*]furan **37** by the dimerization of methyl caffeate **36**.

In their study, Yin et al. achieved the successful synthesis of 2,3-dihydrobenzo[*b*]furan **37** in a 32% yield through the dimerization of methyl caffeate **36** using silver oxide in the presence of anhydrous benzene and acetone (Scheme 7). The primary aim of this study was to explore the potential correlation between IL-25, an endogenous factor secreted by tumor-associated fibroblasts (TAFs), and the inhibition of metastasis in 4T1 mammary tumors in mice [42].

**Figure 15:**
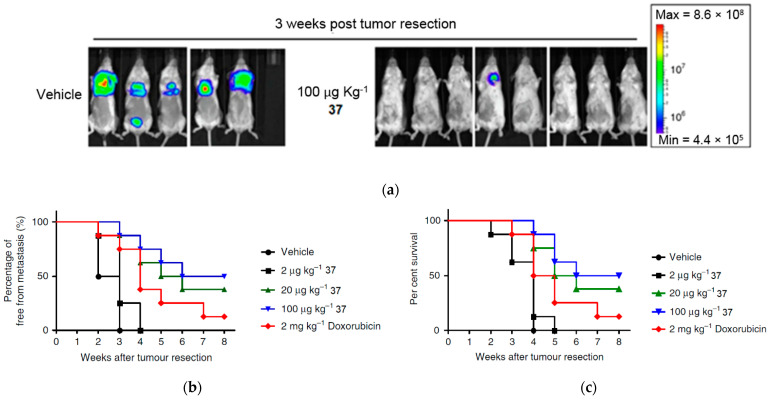
In vivo studies of compound **37** in suppressing metastasis in mouse mammary cells (4T1). (**a**) Bioluminescent images of mouse tumors (*n* = 8 per group) after treatment with PBS and at different doses of compound **37** after tumor resection. Three of the mice died after treatment with PBS before the end of treatment. (**b**) Percentage of metastasis by measurement of luciferase activity in photons over time (*n* = 8 per group). (**c**) Percentage survival of mice after treatment with compound **37**. This is an open-access article distributed under the terms of the Creative Commons CC BY license [42].

The investigation into the antimetastatic effects of compound **37** involved the injection of luciferase-expressing 4T1-Luc2 transgenic mouse cells into the mammary fat pad of the experimental mice [42]. At 15 days after tumor cell implantation, the 4T1 tumors were surgically resected in situ. Over the following 8 weeks, a comparative analysis of tumor metastatic activity and survival was conducted between the control group and the mice treated with compound **37** (Figure 15a–c). By detection of luminescent activity of 4T1-Luc2 cells as an indicator of tumor metastasis, it was observed that treatment with **37** (≥20 µg kg^−1^) significantly suppressed 4T1 cell metastasis to the lung (Figure 15a). In addition, treatment with **37** at a relatively low dose (>20 µg kg^−1^) had considerable antimetastatic activity in comparison with the treatment with Doxorubicin (2 mg kg^−1^) (Figure 15b), used as a control drug for breast cancer. Treatment with **37** also significantly increased the survival rate of mice with tumor resection (Figure 15c). These results demonstrate that the in vivo administration of compound **37** effectively prevents breast tumor metastasis following tumor resection.

**Figure 16:**
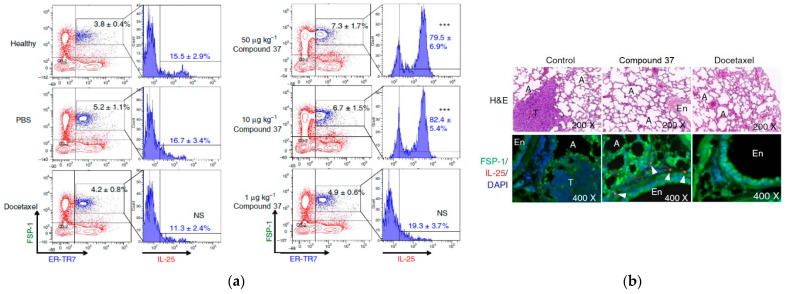
Effect of compound **37** on the regulation of IL-25 expression in tumor-associated fibroblasts. (**a**) Flow cytometry of the population change in FSP-1 + ER-TR7+ cells and their IL-25 expression level in the lungs of mice after treatment with compound **37**, PBS, and docetaxel. Data are reported as mean ± SD (*n* = 3). *** *p* < 0.001. NS—not significant (two-tailed *t*-test). (**b**) IF staining in mouse lung tissues after treatment with compound **37** and docetaxel 21 days after tumor resection. T—tumor, A—alveoli. This is an open-access article distributed under the terms of the Creative Commons CC BY license [42].

Following the previously mentioned findings, the study showed the potential regulatory effects of administering compound **37** in vivo on metastatic tissues [42]. To assess the physiological significance of compound **37** in the modulatory activity, the researchers evaluated the expression of various cytokines secreted in vivo in the lung tissue of the test mice. Remarkably, the findings demonstrated that the administration of compound **37** (at a dosage of 100 µg kg^−1^) had a stimulatory effect on IL-25 activity in pulmonary fibroblasts surrounding the pulmonary artery and vein (Figure 16b). In contrast, little to no IL-25 expression was observed in lung fibroblasts from control and Docetaxel-treated mice, which suggests that the induction of IL-25 expression in fibroblasts of the lung tissue microenvironment specifically resulted from the administration of compound **37**. This finding is intriguing as IL-25 expression is not typically considered a conventional drug target for anticancer medications.

To quantify the change in the cell population of IL-25-expressing lung fibroblasts in response to treatment with compound **37**, the researchers assessed FSP-1 + ER-TR7 + IL-25 + cells from lung tissues of test mice and compared their IL-25 expression levels at 3 weeks after tumor resection. The results unveiled a remarkable increase in the IL-25 fibroblast cell population from 16.7% to 79.5% in **37**-treated mice compared to those treated with PBS (Figure 16a). Furthermore, the population of fibroblasts in FSP-1 + ER-TR7 + cells in mice treated with **37** exhibited a significant dose-dependent increase from 5.2% to 7.3% (Figure 16a), in contrast to docetaxel treatment, which showed no augmentation in the number or level of evaluated fibroblasts. These findings solidify the evidence that compound **37** effectively stimulates lung fibroblasts in an in vivo setting.

**Figure 17:**
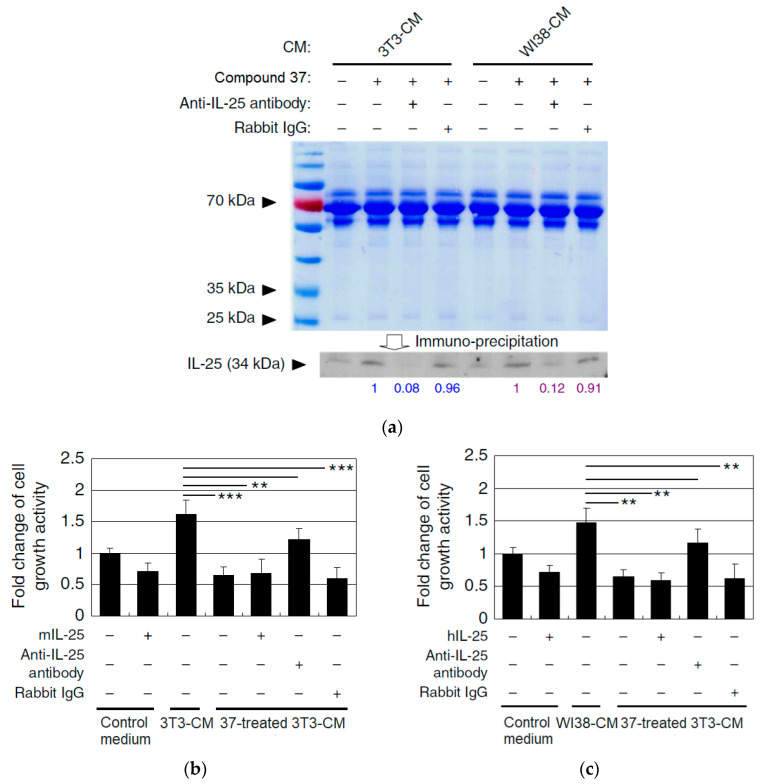
(**a**) Western blot of IL-25 secretion activity in mouse (3T3) and human (WI38) fibroblasts after treatment with compound **37** using Rabbit IgG as the negative control. (**b**) Evaluation of 3T3-CM cytotoxicity levels in 4T1 cells after IL-25 immunodepletion. (**c**) Evaluation of WI38-CM cytotoxicity levels in MDA-MB-231 cells after IL-25 immunodepletion. Data are reported as mean ± SD (*n* = 3). ** *p* < 0.01; *** *p* < 0.001 (two-tailed Student’s *t*-test). This is an open-access article distributed under the terms of the Creative Commons CC BY license [42].

Additionally, the researchers conducted complementary studies to assess the potential suppressive effect of IL-25 secreted by fibroblasts on the growth activity of mammary tumor cells [42]. In this regard, they compared the levels of IL-25 in mouse (4T1) and human (MDA-MB-231) tumor cells treated with compound **37** using an immunoprecipitation method mediated by anti-IL-25 antibodies. To ensure accuracy, the samples were first immunodepleted with 3T3 (3T3-CM) and WI38 (WI38-CM) fibroblasts for IL-25, utilizing anti-rabbit IgG antibody (isotype control) as the negative control for immunodepletion (Figure 17a). The results indicated that the levels of IL-25 secreted in media conditioned with compound **37** treated fibroblasts were significantly higher compared to untreated conditioned media (Figure 17a). Moreover, the researchers detected only a minor fraction of unspecific binding to the protein in the IgG antibody, confirming the high specificity and efficiency of the anti-IL-25 antibody employed. On the other hand, when 4T1 and MDA-MB-231 cells were cultured with 3T3-CM, their growth was notably higher compared to cells cultured solely with fresh medium. This observation implies that in both cases, fibroblasts released critical cellular and molecular factors that contribute to the expansion of tumor cells, potentially influencing the suppression of metastatic mammary tumor cell growth (Figure 17b,c).

**Figure 18:**
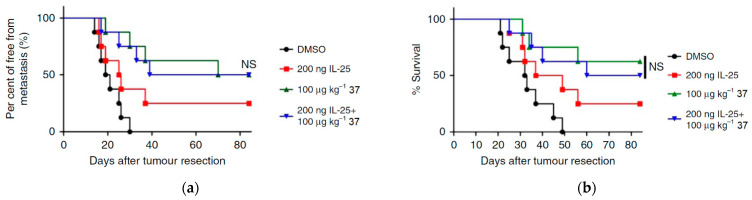
In vivo treatment of compound **37** exhibits comparable antimetastatic activity to IL-25 administration. (**a**) Mice with tumor resection (*n* = 8 per group) were treated with PBS, IL-25, compound **37**, or co-treated with IL-25 and compound **37** after three weeks. Tumor metastases were quantified by measuring luciferase activity over the indicated time course. (**b**) Percentage of survival of mice treated with PBS, compound **37**, or co-treated with IL-25 and compound **37**. The results were analyzed using the log-rank test. NS means not significant. This is an open-access article distributed under the terms of the Creative Commons CC BY license [42].

In their investigation, Yin et al. conducted a comparative analysis of the in vivo treatment effects of IL-25, examining its additive vs. overlapping impact [42]. They discovered that co-administration of compound **37** (at a dosage of 100 µg kg^−1^) and IL-25 (at a dosage of 10 µg kg^−1^) resulted in a similar antimetastatic activity compared to the group of mice treated solely with compound **37** (Figure 18a). The survival rate of mice in the co-treatment group (**37** + Anti-IL-25) was also comparable to the group treated with compound **37** alone, in contrast to the untreated group (Figure 18b). These findings suggest that the in vivo antimetastatic effect of **37** can be effectively substituted by the administration of exogenous IL-25. 

**Figure 19:**
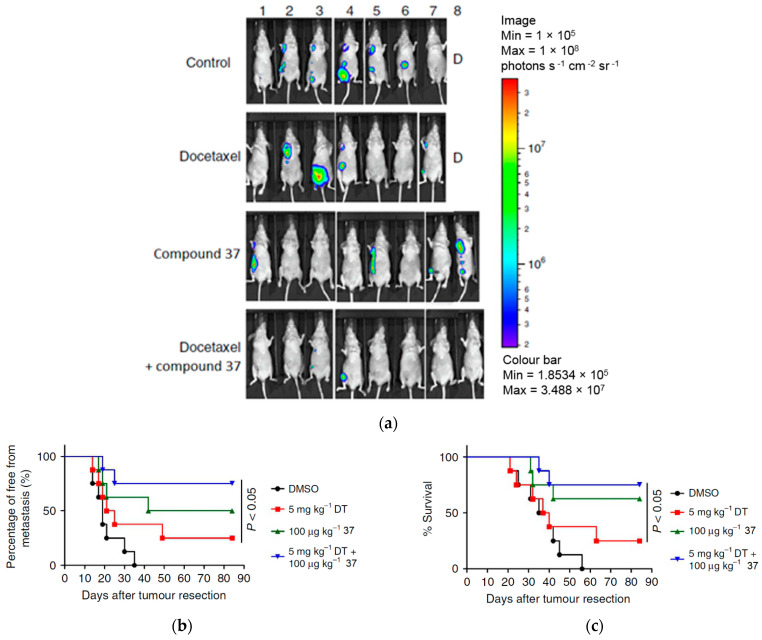
The antimetastatic effect after administration of compound **37** in MDA-MB-231 cells in combination with docetaxel. (**a**) Bioluminescent images of mice with tumor portions (*n* = 8 per group) after treatment with PBS, compound **37**, docetaxel, and co-treatment with docetaxel and compound **37** for three weeks and after tumor resection. (**b**) Levels of tumor metastasis by measurement of luciferase activity in photons in the treated mice over time. (**c**) Percentage survival of mice treated with docetaxel and those co-treated with docetaxel and compound **37**. This is an open-access article distributed under the terms of the Creative Commons CC BY license [42].

Moreover, the study evaluated the combined effect of compound **37** and docetaxel in suppressing the metastatic activities of human MDA-MB-231-Luc2 cells in mice through bioluminescent studies following the resection of mammary tumor tissues in the experimental mice (Figure 19a). The results demonstrated that the treatment with compound **37** (100 µg kg^−1^) and docetaxel (5 mg kg^−1^) showed significantly higher antimetastatic activity than the treatment with docetaxel alone (Figure 19b). As a result, the test mice receiving the combination of compound **37** and docetaxel exhibited a higher survival rate compared to those receiving single treatments (Figure 19c). These findings suggest a complementary effect on the anticancer activity of docetaxel when combined with compound **37**, effectively suppressing the metastatic activities of tumor cells by modulating the tumor-associated microenvironment.

**Figure 20:**
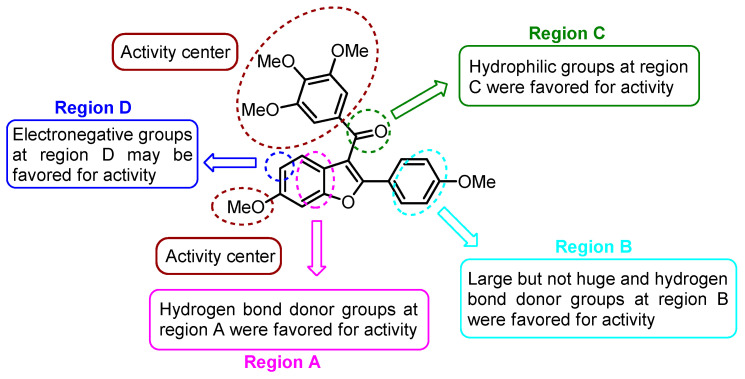
3D-QSAR studies in benzo[*b*]furan derivatives **43a**–**e**.

On a different note, Quan et al. conducted a molecular modeling study involving 64 Combretastatin A-4 analogs based on five-membered heterocycles. Their objective was to explore the development of novel anticancer agents by using 3D-QSAR, molecular docking, and molecular dynamic (MD) simulation [43]. Within the 3D-QSAR approach, both CoMFA and CoMSIA models were prepared for both the training and test sets. The CoMFA model incorporated steric and electrostatic fields, while the CoMSIA model included steric, electrostatic, hydrophobic, hydrogen bond donor, and hydrogen bond acceptor fields. Three-dimensional contour maps for both models were performed using the “Stdev*Coeff” field type. By analyzing the results obtained from the study, the researchers identified essential structure–activity relationships, which highlighted substitutions that could enhance biological activity. This was summarized in A–D regions, as shown in Figure 20. Building on this information, they designed five novel benzo[*b*]furan derivatives. The structures and predicted pIC_50_c values of compounds **43a**–**e** are provided in Table 11. Although these data indicate the presence of inhibitory activity for the designed compounds, they were not comparable to CA-4.

Additionally, the researchers conducted 20 ns molecular dynamics (MD) simulations and binding free energy calculations using the Amber 12.0 package [43]. The stability of the tubulin–inhibitor complex in the designed compounds **43a**–**e** was evaluated, employing the general Amber force field (gaff) for ligands and the ff99SB force field for proteins. In this study, binding free energy calculations were performed using both MM/GBSA and MM/PBSA methods. The results showed that the calculated binding free energies using MM/GBSA for CA-4 and the inhibitors **43a**–**e** were as follows: −34.32, −57.52, −54.41, −55.78, −50.77, and −55.56 kcal mol^−1^, respectively. Meanwhile, the MM/PBSA results revealed the binding free energies of −21.54, −43.45, −42.03, −40.99, −39.79, and −36.23 kcal mol^−1^ for CA-4 and the inhibitors **43a**–**e**, respectively. Among the five newly designed compounds, **43a** exhibited the most negative binding free energy, suggesting it has the potential for the best inhibitory activity within the series. These computational findings provide valuable insights into the potential efficacy of the designed compounds and can guide further experimental investigations to validate their inhibitory activity against tubulin.

**Scheme 8:**
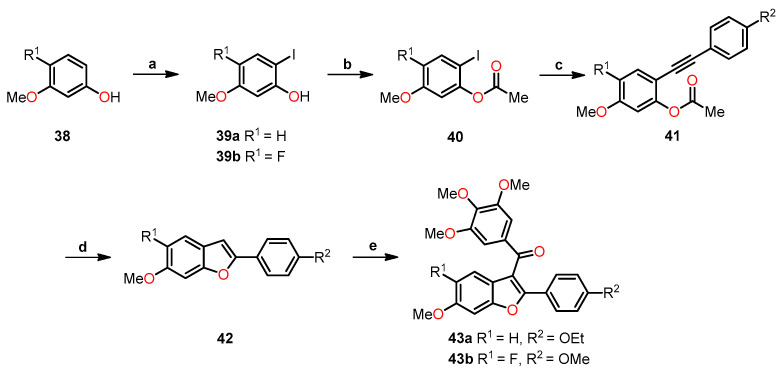
Synthesis of benzo[*b*]furan derivatives **43a** and **43b** and the reaction conditions: (**a**) AgOTFA, I_2_, 24 h, r.t, 52% for **39a** and 39% for **39b**; (**b**) Ac_2_O, pyridine, 4 h, r.t, 78% for **40a** and 81% for **40b**; (**c**) 1-ethynyl-4-ethoxybenzene or 1-ethynyl-4-methoxybenzene, PdCl_2_(PPh_3_)_3_, CuI, Et_3_N, DMF, 100 °C, 6 h, 46% for **41a** and 32% for **41b**; (**d**) K_2_CO_3_, CH_3_OH, 60 °C, 16 h, 40% for **42a** and 25% for **42b**; (**e**) 3,4,5-trimethoxybenzoyl chloride, SnCl_2_, CH_2_Cl_2_, 3 h, r.t, 70% for **43a** and **43b**.

Using the binding free energy calculations, two of the designed compounds, **43a** and **43b**, were selected for synthesis. The synthetic process involved several sequential steps to obtain the desired compounds. Firstly, the synthesis began with the iodination of methoxyphenol **38** using a catalytic amount of AgOTFA in chloroform at room temperature for 24 h to afford the iodinated compound **39** (Scheme 8). Subsequently, the acetylation of the iodine-phenol **39** was carried out in the presence of acetic anhydride in pyridine at room temperature for 4 h to yield the acetate derivative **40**. Next, the Sonogashira coupling of compound **40** with either 1-ethynyl-4-ethoxybenzene or 1-ethynyl-4-methoxybenzene was conducted in the presence of catalytic PdCl_2_(PPh_3_)_3_, leading to the formation of alkyne **41**. The intermediates underwent an intramolecular cyclization reaction mediated by K_2_CO_3_ in methanol at 60 °C for 16 h to produce the benzo[*b*]furan derivative **42**. Finally, a Friedel–Crafts reaction was performed in the presence of 3,4,5-trimethoxybenzoyl chloride, followed by the addition of compound **42** and tin (IV) chloride to deliver compound **43**. By employing this multi-step synthetic approach, the researchers successfully synthesized compounds **43a** and **43b**, paving the way for further evaluation of their potential as benzo[*b*]furan-based anticancer agents.

The in vitro antiproliferative activity of compounds **43a**–**b** was evaluated against six human cancer cell lines, and their tubulin inhibition was assessed, using CA-4 and CA-4P as standard drugs. As shown in Table 12, the compound **43a** (R^1^ = H, R^2^ = Oet) exhibits the highest activity with IC_50_ values of 1.37, 8.99, 1.31, and 0.91 µM against A549, HeLa, HepG2, and MCF-7 cell lines, respectively [43]. Compound **43a** exhibits comparable and even superior activity than CA-4 in the mentioned cell lines. Specifically, it is 4.4-fold more active against A549 cells and 12.2-fold more active against HepG2 cells than CA-4. In addition, compound **43a** showed only comparable activity with compound CA-4P in the MCF-7 cell line. On the other hand, compound **43b** (R^1^ = H, R^2^ = Ome) exhibited remarkable activity against A549 and HepG2 cells, with IC_50_ values of 6.87 and 4.75 µM, respectively, which is comparable to CA-4 (IC_50_ = 5.99 and 16.04 µM, respectively). Finally, in a tubulin polymerization assay, compound **43a** demonstrated potent inhibition of tubulin polymerization, with an IC_50_ value of 0.86 µM, which is comparable to CA-4 (IC_50_ = 0.88 µM) and superior to CA-4P (IC_50_ = 4.79 µM).

**Scheme 9:**
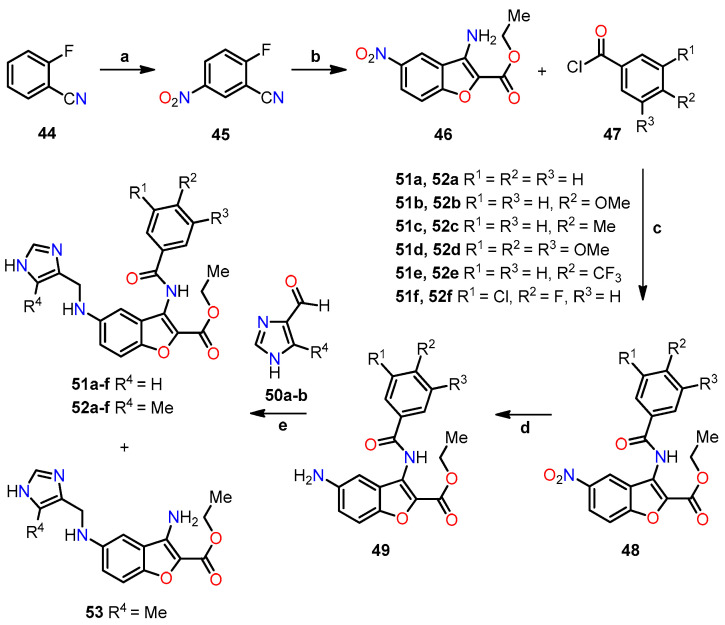
Synthesis of 3-benzoylamino-5-(1*H*-imidazol-4-yl)methylaminobenzo[*b*]furan derivatives **51a**–**f**, **52a**–**f**, and **53**, and the reaction conditions: (**a**) HNO_3_/H_2_SO_4_, N_2_, 0 °C, 2 h; (**b**) ethyl glycolate, K_2_CO_3_, anhydrous DMF, 100 °C, 12 h, 63%; (**c**) pyridine, r.t, 12 h, 62–86%; (**d**) 10% Pd/C, H_2_, ethanol, r.t, 2 h, 43–93%; (**e**) NaBH_3_CN, ethanol, AcOH, r.t, 6–24 h, 23–65% for **51a**–**f**, 15–90% for **52a**–**f**, and 10–23% for **53**.

Similarly, Lauria et al. conducted the synthesis of a novel series of 3-benzoylamino-5-(1*H*-imidazol-4-yl)methylaminobenzo[*b*]furans **51**–**53** and subsequently evaluated their potential as antitumor agents [44]. The synthetic route involved a sequence of steps (Scheme 9). Initially, 2-fluorobenzonitrile **44** underwent a nitration reaction in the presence of a mixture of concentrated nitric and sulfuric acids under a nitrogen atmosphere at 0 °C for 2 h to afford 2-fluoro-5-nitrobenzonitrile **45**. Afterward, ethyl glycolate was utilized for nucleophilic displacement in the presence of K_2_CO_3_ and anhydrous DMF at 100 °C for 12 h, facilitating in situ intramolecular cyclization and giving rise to the 3-amino-benzo[*b*]furan derivative **46**. Following this, intermediate **46** underwent acyl substitution with benzoyl chloride **47** in pyridine, serving as both base and solvent, at room temperature for 12 h, yielding compound **48**, containing amide functionality at the C–3 position. The subsequent reduction of the nitro group in compound **48** was performed through hydrogenation using a Parr hydrogenation apparatus at 500 psi in the presence of Pd/C (10%) as a catalyst in ethanol at room temperature for 2 h, yielding 5-amino-benzo[*b*]furan derivative **49**. Finally, the compounds **51a**–**f** and **52a**–**f** were obtained through a reductive amination with imidazole-4-carbaldehyde **50** using sodium cyanoborohydride as a selective reducing agent in a mixture of ethanol and acetic acid at room temperature for 6–24 h. In this final step, compound **53** was also isolated using carbaldehyde **50b**, wherein evidence of the hydrolysis of the amide functionality was observed. These novel compounds hold promise as potential antitumor agents and warrant further investigation to assess their efficacy in cancer treatment.

**Figure 21:**
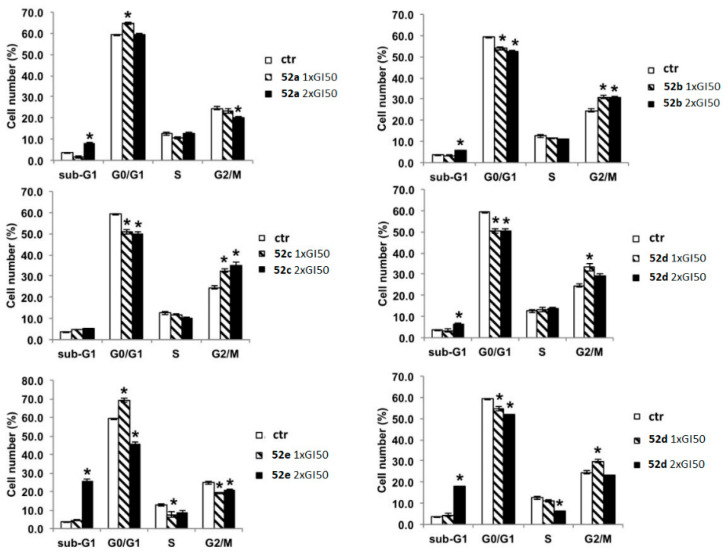
Analysis of the influence of the concentrations of compounds **52a**–**f** on the cell cycle of the HeLa cell line after 24 h using flow cytometry. Data are expressed as the mean of two independent experiments. Statistical analyses were performed using the Student’s *t* test to determine the differences between the datasets. * *p* < 0.05 denote significant differences from untreated control cells. Reproduced with permission from ref. [44]. Copyright John Wiley & Sons Inc., 2023.

The biological studies focused on the antiproliferative activity in HeLa and MCF-7 cell lines using the MTT assay, cell cycle analysis, and in silico assessment [44]. Table 13 shows the GI_50_ values of compounds **51a**–**f** and **52a**–**f**. Notably, the insertion of a methyl group in the imidazole ring increased activity for compounds **52a** (R^1^ = R^2^ = R^3^ = R^4^ = H), **52e** (R^1^ = H, R^2^ = CF_3_, R^3^ = R^4^ = H), and **52f** (R^1^ = Cl, R^2^ = F, R^3^ = R^4^ = H) without substitution on the benzoyl moiety (**52a** vs. **51a**), or functionalized with 4-trifluoromethyl- (**52e** vs. **51e**) and 3-chloro-4-fluoro- (**52f** vs. **51f**) substituents. Compound **52f** exhibited the highest activity against HeLa and MCF-7 cell lines, with GI_50_ values of 2.14 µM and 1.55 µM, respectively. In cell cycle analysis, compounds **52b**, **52c**, **52d**, and **52f** showed significant suppression of the G0/G1 phase and an accumulation of cells in G2/M at 1xGI_50_ concentrations (Figure 21). However, at 2xGI_50_ concentrations, changes in the distribution profile were observed. Albeit compounds **52a** and **52e** did not show a significant impact on the cell cycle at the evaluated concentrations, they induced G0/G1 arrest at 2.5xGI_50_ and 5xGI_50_ concentrations, which correlated with their antiproliferative effects in other phases.

**Figure 22:**
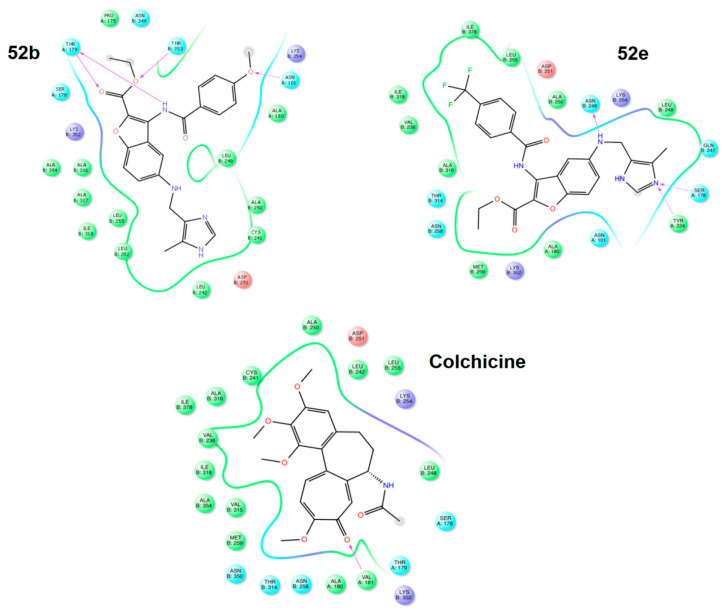
Ligand interaction maps of compounds **52b**, **52e**, and colchicine. Reproduced with permission from ref. [44]. Copyright John Wiley & Sons Inc., 2023.

In the in silico studies, the researchers assessed the binding modes of each derivative at the colchicine binding site on tubulin, considering the involved amino acid residues [44]. To facilitate this analysis, they obtained the crystal structure of tubulin bound to colchicine from the PDB database (PDB ID code: 4O2B) and extracted the tubulin dimer with colchicine (chains A and B) from the protein model. Table 14 shows the favorable induced docking protocol (IFD) scores for all ligand–tubulin complexes, with compounds **51** and **52** showing affinity similar to colchicine and higher than CA-4. Notably, significant differences in the binding complexes were observed among the evaluated amino acids. These benzo[*b*]furans displayed strong interactions with amino acids Alaα180, Serα178, Ileβ318, Alaβ316, Leuβ255, Leuβ248, and Lysβ254, as indicated by the IFD scores. Interestingly, compounds **52b**–**d**,**f** presented an aromatic ring in contact with Cysβ241, a crucial and distinctive feature for identifying new antitubulin molecules. A more detailed illustration of these interactions is found in Figure 22, depicting the ligand interaction maps of compounds **52b** and **52e** with colchicine as a reference.

**Scheme 10:**
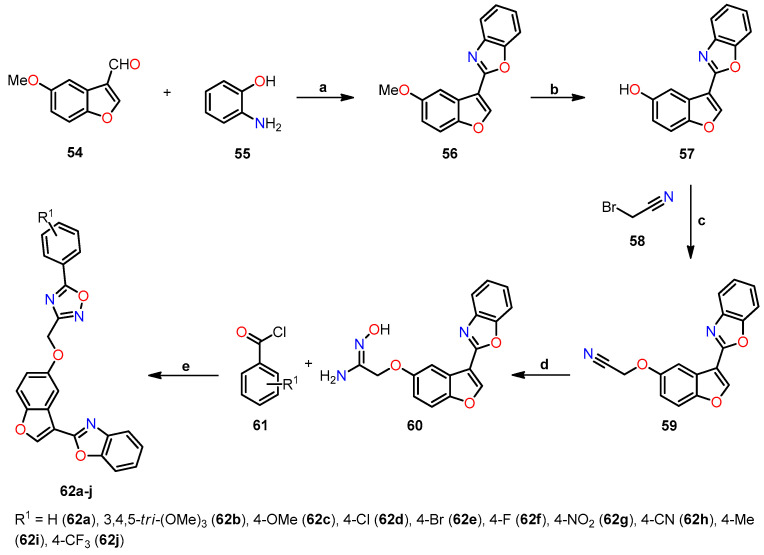
Synthesis of benzo[*b*]furan-3-yl-1,3-benzoxazole derivatives **62a**–**j**, and reaction conditions: (**a**) EtOH, reflux, 4 h, then AcOH, Pb(Oac)_4_, r.t., 1 h, 79%; (**b**) DCM, BBr_3_, r.t., 5 h, 88%; (**c**) acetone, K_2_CO_3_, reflux, 5 h, 94%; (**d**) EtOH, NH_2_OH. HCl, K_2_CO_3_, reflux, 3 h, 92%; (**e**) pyridine, r.t., 4 h, 63–93%.

Afterward, Pervaram et al. synthesized 1,2,4-oxadiazole-fused benzo[*b*]furan derivatives **62a**–**j** and assessed their antiproliferative activity against four human cancer cell lines, including A549 (lung), MCF-7 (breast), A375 (melanoma), and HT-29 (colon), using the MTT method [45]. The synthetic route for compounds **62a**–**j** is shown in Scheme 10. The synthesis began with the reaction of 5-methoxybenzofuran-3-carbaldehyde **54** with 2-aminophenol **55** in refluxing ethanol for 4 h, leading to the 2,3-dihydrobenzo[*d*]oxazole intermediate, which was oxidized to 2-(5-methoxybenzofuran-3-yl)benzo[*d*]oxazole **56** by adding Pb(Oac)_4_ and acetic acid at room temperature for 1 h. Next, compound **56** reacted with BBr_3_ in anhydrous CH_2_Cl_2_ at room temperature for 5 h to give 3-(benzo[*d*]oxazol-2-yl)benzofuran-5-ol **57**, which is *O*-alkylated with 2-bromoacetonitrile **58** and K_2_CO_3_ in refluxing acetone for 5 h to furnish compound **59**. Subsequently, a nucleophilic addition reaction between **59** and hydroxylamine hydrochloride in the presence of K_2_CO_3_ in refluxing ethanol for 3 h yielded acetaminide **60**. Finally, compound **60** was reacted with the different benzoyl chloride **61** using pyridine at room temperature for 4 h to obtain 1,2,4-oxadiazole-fused benzo[*b*]furan derivatives **62a**–**j** in yields ranging from 63% to 93%.

The antiproliferative activity of all synthesized compounds **62a**–**j** was assessed against four cancer cell lines using the MTT method and CA-4 as the standard drug (Table 15) [45]. Notably, compounds **62b** (R^1^ = 3,4,5-*tri*-(Ome)_3_), **62g** (R^1^ = 4-NO_2_), **62h** (R^1^ = 4-CN), and **62j** (R^1^ = 4-CF_3_) exhibited comparable and, in some cases, even higher potency than CA-4, with IC_50_ values ranging from 0.012 to 1.45 µM for these compounds, while CA-4 had IC_50_ values ranging from 0.11 to 0.93 µM.

**Scheme 11:**
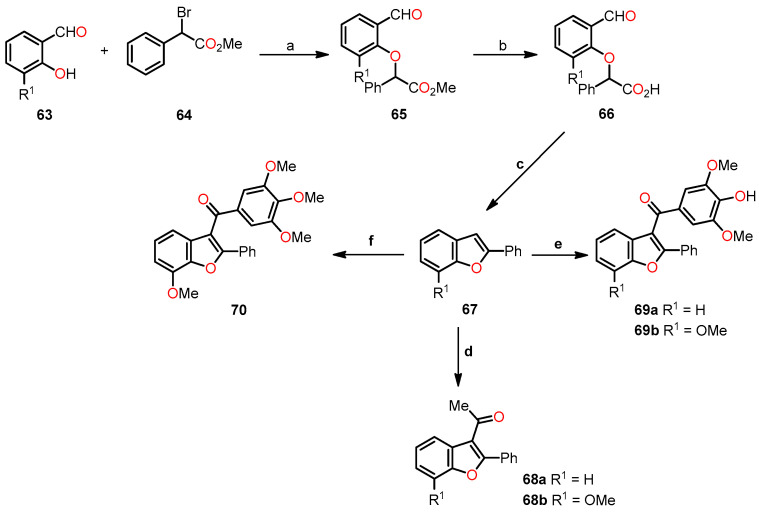
Synthesis of 2-phenylbenzo[*b*]furan derivatives **68a**–**b**, **69a**–**b**, and **70**, and the reaction conditions: (**a**) K_2_CO_3_, DMF, 92–94 °C, 4 h, 61–76%; (**b**) KOH (10%), MeOH, 75–80 °C, 2 h, then HCl (10%), r.t., 70–72%; (**c**) Ac_2_O, AcONa, 125–130 °C, 4.5 h, 90–97%; (**d**) Ac_2_O, Amberlyst-15, 1,2-dichloroethane, reflux, 6 h, 69–72%; (**e**) 3,4,5-trimethoxybenzoyl chloride, AlCl_3_, 1,2-dichloroethane, 45 °C, 3.5 h, 68–76%; (**f**) 3,4,5-trimethoxybenzoyl chloride, Amberlyst-15, 1,2-dichloroethane, reflux, 6.5 h, 69%.

Similarly, Kwiecień et al. conducted a study on the synthesis and evaluation of functionalization at position 3 of 2-phenyl- and 2-alkylbenzo[*b*]furans as potential antitumor agents [46]. The synthesis of 2-phenylbenzo[*b*]furan **67** involved a three-step reaction process (Scheme 11). Firstly, 2-hydroxybenzaldehyde **63** was *O*-alkylated with methyl 2-bromo-2-phenylacetate **64** in the presence of K_2_CO_3_ and DMF at 92–94 °C for 4 h to afford methyl 2-(2-formylphenoxy)-2-phenylacetate derivative **65** with yields in the range of 61–76%. Secondly, basic hydrolysis of ester **65** in methanol refluxing for 2 h, and then protonation yielded 2-(2-formylphenoxy)-2-phenylacetic acid **66** in acceptable yields (70–72%). Lastly, an intramolecular cyclization of compound **66** using a mixture of Ac_2_O and AcONa at 125–130 °C for 4.5 h produced the benzo[*b*]furan **67** in excellent yields (90–97%).

Later, the researchers focused on the acylation of 2-phenylbenzo[*b*]furan **67**. Initially, the acetylation of **67** with Ac_2_O in the presence of Amberlyst-15 in 1,2-dichloroethane refluxing for 6 h afforded 2-phenylbenzo[*b*]furan-3-yl)ethan-1-ones **68a**–**b** in acceptable yields (69–72%). Then, (4-hydroxy-3,5-dimethoxyphenyl)-(2-phenylbenzo[*b*]furan-3-yl)methanones **69a**–**b** were synthesized in 68–76% yields through an AlCl_3_-mediated acylation with 3,4,5-trimethoxybenzoyl chloride in 1,2-dichloroethane at 45 °C for 3.5 h. Finally, the phenylbenzo[*b*]furan-3-yl-(3,4,5-trimethoxyphenyl)methanone **70** was obtained in a 69% yield through an Amberlyst-mediated acylation with 3,4,5-trimethoxybenzoyl chloride in 1,2-dichloroethane refluxing for 6.5 h.

**Scheme 12:**
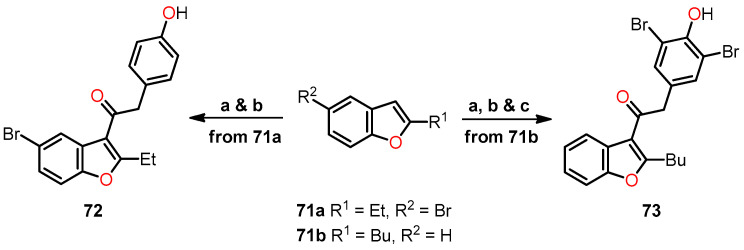
Synthesis of 2-alkylbenzo[*b*]furan derivatives **72** and **73**, and the reaction conditions: (**a**) 2-(4-methoxyphenyl)acetyl chloride, AlCl_3_, 1,2-dichloroethane, 0–15 °C, 6 h; (**b**) pyridine hydrochloride, reflux, 10–12 min; (**c**) HBr (20%), NaClO_3_, r.t., 2 h. The authors have not provided information regarding the yields.

A synthetic route for obtaining 3-phenyl-functionalized 2-alkylbenzo[*b*]furans **72** and **73** was developed (Scheme 12). Firstly, 1-(5-bromo-2-ethylbenzo[*b*]furan-3-yl)-2-(4-hydroxyphenyl)ethanone **72** was synthesized from 5-bromo-2-ethylbenzo[*b*]furan **71a** through an acylation with 4-methoxyphenylacetyl chloride, followed by demethylation to convert the methoxy group into a hydroxy group. Secondly, 2-(3,5-dibromo-4-hydroxyphenyl)-1-(2-butylbenzo[*b*]furan-3-yl)ethanone **73** was prepared from 2-butylbenzo[*b*]furan **71b** by carrying out three sequential reactions: acylation with 4-methoxyphenylacetyl chloride, demethylation of the methoxy group, and bromination.

The biological evaluation encompassed the examination of compounds **68b**, **69a**–**b**, **70**, **72**, and **73**, focusing primarily on antiproliferative studies, flow cytometry, confocal microscopy imaging, and the tubulin polymerization assay, among other complementary analyses [46]. For the in vitro antiproliferative assessment, the benzo[*b*]furan derivatives were tested against the A375 cancer cell line using a cell proliferation reagent WST-1 assay (Table 16). The results revealed that compounds **69a** (R^1^ = H), **69b** (R^1^ = Ome), and **70** exhibited the most potent antiproliferative activity, displaying IC_50_ values of 2.85, 0.86, and 0.09 µM, respectively. Conversely, compounds **68b** (R^1^ = Ome), **72**, and **73** demonstrated low activity, with IC_50_ values exceeding 100 µM.

Flow cytometry analysis employed an apoptosis detection kit FITC Annexin V to evaluate apoptosis and necrosis in cells after 48 h of incubation with benzo[*b*]furan derivatives [46]. The results from the flow cytometry demonstrated minimal cytotoxicity for compounds **68b**, **72**, and **73**, with approximately 90% of live cells, which is similar to the control group. In contrast, compounds **69a**, **69b**, and **70** exhibited a significant increase in late apoptotic cells, with percentages reaching 57.63%, 71.21%, and 58.52%, respectively. These findings highlight the potent anticancer activity of 3-aryl-2-phenylbenzo[*b*]furan derivatives.

In addition, a complementary study on cell cycle distribution was performed, revealing that the 3-aryl-2-phenylbenzo[*b*]furan derivatives induced the accumulation of A375 cells in a tetraploid state (4N), resulting in a decrease in the percentage of cells in the G0/G1 phase. Specifically, compounds **69a**, **69b**, and **70** led to 66.34%, 58.86%, and 63.62% of A375 cells in the G2/M phase, respectively. In contrast, compounds **68b**, **72**, and **73** did not show significant differences in the percentages of A375 cells in the G0/G1 and G2/M phases compared to untreated control cells. These findings provide valuable insights into the mode of action of the 3-aryl-2-phenylbenzo[*b*]furan derivatives and highlight their potential as effective anticancer agents.

**Figure 23:**
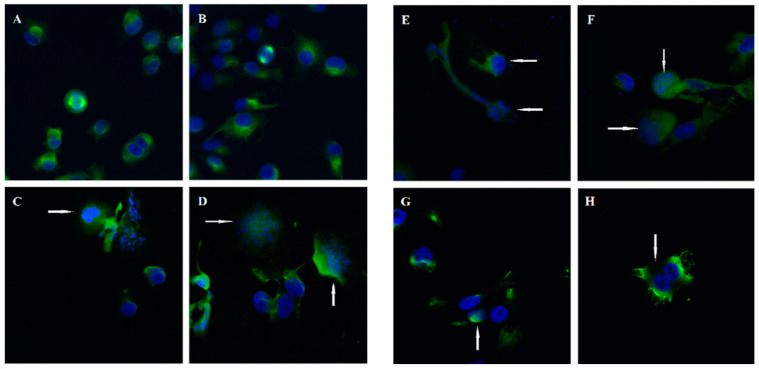
Confocal microscopy images in A375 cells: (**A**,**B**) control cells; (**C**,**D**) compound **69a**; (**E**,**F**) compound **69b** after 7 h of incubation and at a concentration of 100 µM; (**G**,**H**) compound **70** at 10 µM with *α*-tubulin stained (green) and chromosomes (blue). Arrows indicate abnormal spindles. Reproduced with permission from ref. [46]. Copyright Elsevier Inc., 2023.

After analyzing the cell cycle distribution results, Kwiencień et al. conducted a confocal microscopy analysis [46]. Cells were incubated with the tested compounds for 7 h, fixed, and then stained for *α*-tubulin and chromosomes. The control cells displayed well-defined bipolar spindle formation with chromosome alignment at the metaphase central plate or anaphase distribution (Figure 23A,B). However, cells treated with 3-aryl-2-phenylbenzo[*b*]furans exhibited diverse phenotypes, characterized by enlarged nuclei and the absence of a visible mitotic spindle (Figure 23C–H). Notably, compound **70** showed a distinct phenotype with binuclear or enlarged nuclei. These observations suggest that the disparity between the tested compounds and control cells was specifically evident during mitosis, indicating a specific mitotic activity of the compounds. This finding sheds significant light on the potential of these compounds as specific mitosis-targeting agents.

**Figure 24:**
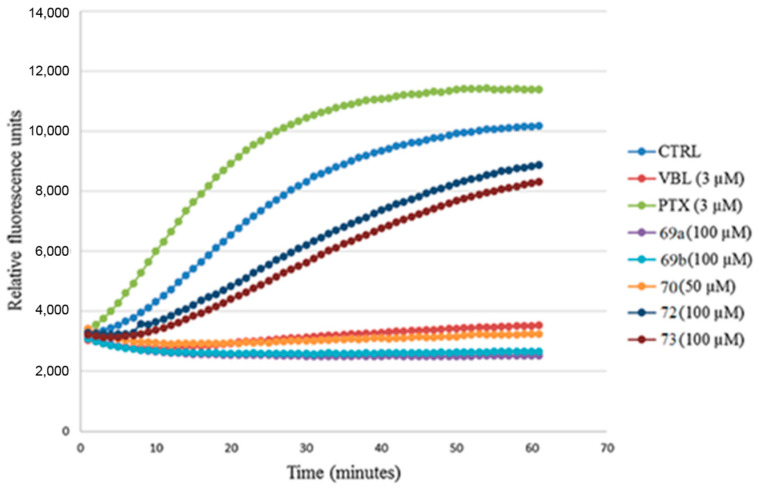
Analysis of the inhibition of tubulin polymerization in benzo[*b*]furan derivatives **69a**, **69b**, **70**, **72**, and **73**. Reproduced with permission from ref. [46]. Copyright Elsevier Inc., 2023.

Finally, the effects of benzo[*b*]furan derivatives on tubulin polymerization were evaluated based on fluorescence [46]. Paclitaxel (PTX), vinblastine (VBL), and DMSO (0.2%) were used as control and reference compounds. As depicted in Figure 24, DMSO had no direct effect on tubulin polymerization. In contrast, the reference compounds (PTX and VBL) interacted with tubulin, resulting in alterations to the normal polymerization curve. Upon comparing the curves of VBL, **69a**, **69b**, and **70**, it became evident that these compounds were the most effective in inhibiting tubulin polymerization, as indicated by a decrease in *V*_max_ (maximum slope values for the growth phase) and a reduction in the final mass of the protein polymer. These findings align with the observations from confocal microscopy and flow cytometry analyses, where the inhibition of tubulin polymerization led to the prevention of mitotic spindle formation, resulting in the presence of polyploid nuclei and cell cycle arrest at 4N. These collective data provide compelling evidence for the significant impact of the tested compounds on tubulin polymerization and their potential as potent agents affecting cell division and proliferation.

**Scheme 13:**
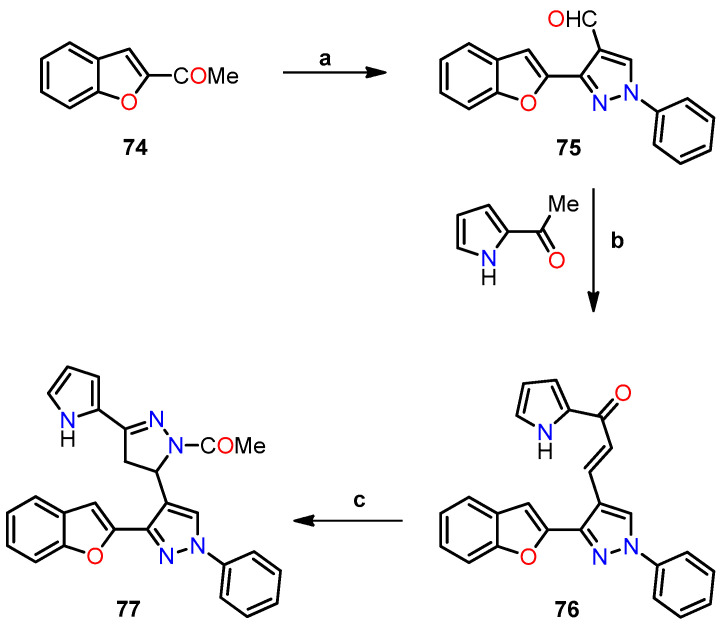
Synthesis of benzofuran–pyrazole hybrid **77**, and reaction conditions: (**a**) PhNHNH_2_, then POCl_3_, DMF; (**b**) EtOH, NaOH, 88%; (**c**) N_2_H_4_/AcOH, 85%.

Anwar et al. pursued a unique approach and conducted preliminary studies, which revealed a promising anticancer activity in a benzofuran–pyrazole hybrid **77** [47]. Encouraged by these favorable findings, the researchers explored the potential benefits of its nanorange form, aiming to investigate the influence of the nanorange and its effect on the cytotoxic potency of the hybrid **77** [48]. The synthesis of hybrid **77** involved a three-step process: (1) the conversion of 1-(benzofuran-2-yl)ethanone **74** into the pyrazole-4-carbaldehyde **75** through the Vilsmeier–Haack reaction; (2) Claisen–Schmidt condensation of compound **75** with 2-acetylpyrrole to give chalcone **76** in an 88% yield; and, finally, (3) the cyclocondensation of compound **76** with hydrazine hydrate in acetic acid to afford benzofuran–pyrazole hybrid **77** in an 85% yield (Scheme 13).

On the other hand, nanoparticles of the benzofuran–pyrazole hybrid **77** were synthesized using the nanoprecipitation method and exhibited sizes ranging from 3.8 to 5.7 nm. The characterization of these nanoparticles involved transmission microscopy (TEM) to confirm spherical shape and average size. Additionally, the surface charge and stability of the nanoparticles were analyzed utilizing the Malvern Zetasizer nano Zs instrument. These results indicated that the nanoparticles had an average size of 3.8–5.7 nm and a zeta potential of −27.3 mV, with a polydispersity index (PDI) of 0.77, confirming their uniformity and stability.

The anticancer activity of the benzofuran–pyrazole hybrid **77** and its nanoparticles was assessed against two breast cancer cell lines, MCF-7 and MDA-MB-231, using an MTT assay and doxorubicin as a standard drug (Table 17) [48]. The results showed that the nanoparticles of hybrid **77** exhibited the highest cytotoxic activity against both MCF-7 and MDA-MB-231 cell lines with IC_50_ values of 1 and 0.6 nM, respectively, outperforming doxorubicin (IC_50_ = 620 nM in both cases). In contrast, hybrid **77** showed lower activity than its nanoparticles against both cell lines, with IC_50_ values of 7 and 10 nM. This difference in activity can be attributed to the high surface area/volume ratio of nanoparticles, which allows for selective targeting of cells and tissues, and more effective interactions compared to hybrid **77** (>100-fold). Finally, IC_50_ values of hybrid **77** and its nanoparticles showed over a 1000-fold difference when targeting normal breast cells MCF-12A compared to cancer cells, indicating their safety profiles in normal cells.

**Figure 25:**
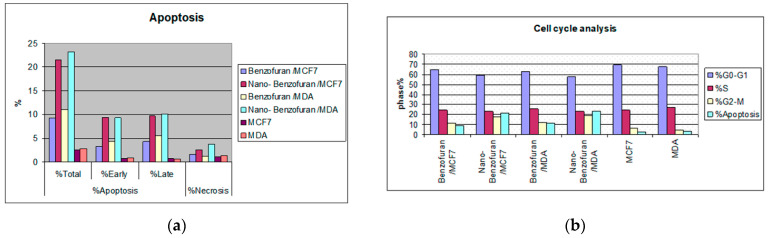
Flow cytometry analysis, showing (**a**) apoptosis and (**b**) cell cycle analysis of the benzofuran–pyrazole hybrid **77** and its nanoparticles in MCF-7 and MDA-MB-231 cell lines. This is an open-access article distributed under the terms of the Creative Commons CC BY license [48].

The researchers performed complementary analyses to explore the cell cycle, the effect on caspase-3/p53/Bax/Bcl-2 levels, and PARP-1 cleavage [48]. For the cell cycle analysis, the effects of hybrid **77** and its nanoparticles were evaluated against MCF-7 and MDA-MB-231 cell lines using flow cytometry, comparing the results with a DMSO control. As shown in Figure 25a, both the hybrid **77** and its nanoparticles induced apoptotic cells, resulting in percentages of 9.18% and 21.54% for the MCF-7 line, and 11.09% and 23.17%, for the MDA-MB-231 line, respectively. These findings underscore the significantly greater potency of the nanoparticles of **77**, being twice as effective as the hybrid **77** against both cell lines tested. Moreover, exposure to the hybrid **77** and its nanoparticles caused a noticeable disruption in cell cycle distribution, with percentages of 11.26% and 17.52% observed in MCF-7, and 12.11% and 19.24% in MDA-MB-231, respectively (Figure 25b). These results suggest that the inhibitory potency was predominantly associated with the nanoparticles of **77**, particularly in the G2/M phase.

In the study focusing on caspase-3/p53/Bax/Bcl-2 levels, the researchers employed an enzyme-linked immunosorbent assay (ELISA) to investigate two cancer cell lines [48]. As shown in Table 18, hybrid **77** exhibited a notable increase in caspase-3 levels (>5-fold) compared to untreated cells. However, the nanoparticles of **77** demonstrated an even more remarkable effect, surpassing the impact of hybrid **77**, with caspase-3 levels elevated by over 17-fold vs. untreated cells. Moreover, p53 levels showed an approximately 7-fold increase for hybrid **77** and a remarkable 14-fold increase for nanoparticles of **77** against both cell lines tested. Additionally, Bax levels displayed a significant increase of 5- to 13-fold, while Bcl-2 levels decreased by 4- to 7-fold in both cell lines compared to the untreated cells.

In the final phase of the study, the researchers conducted a PARP-1 cleavage assay, using staurosporine as the standard drug [48]. As depicted in Table 19, hybrid **77** did not exhibit a considerable inhibitory effect on both cancer cell lines compared to staurosporine. However, the nanoparticles of **77** showed a remarkable inhibitory effect, proving to be 4 and 10 times more potent than the hybrid **77** for the MCF-7 and MDA-MB-231 cell lines, respectively. Notably, the nanoparticles of **77** exhibited an IC_50_ value of 6 nM, whereas staurosporine had an IC_50_ value of 8 nM for the MDA-MB-231 line.

**Scheme 14:**
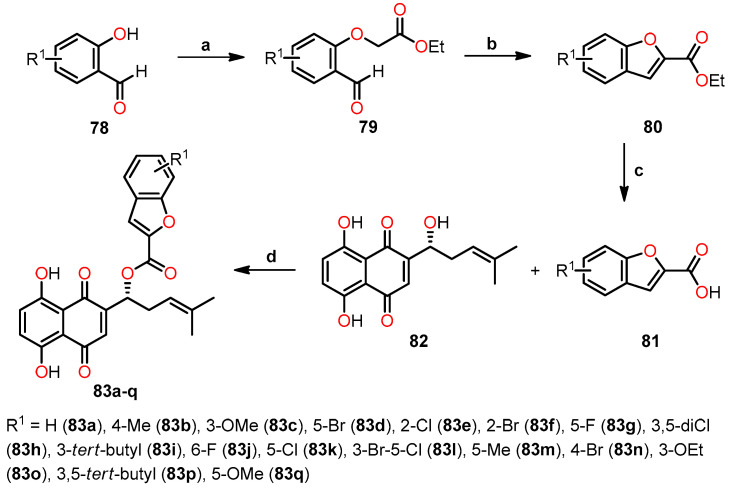
Synthesis of shikonin–benzo[*b*]furan hybrids **83a**–**q**, and reaction conditions: (**a**) KI, DMF, r.t, 30 min, then BrCH_2_CO_2_Et, 2 h, r.t; (**b**) K_2_CO_3_, DMF, 120 °C, 3 h; (**c**) NaOH (20%), DMF, 60 °C, 30 min; (**d**) DCC, DMAP, CH_2_Cl_2_, 0 °C, 8 h, 20–36%. Yields for compounds **79**, **80**, and **81** have not been reported by the authors.

Shikonin–benzo[*b*]furan hybrids have shown potential as inhibitors of tubulin polymerization. To further explore this, Shao et al. synthesized the compound **79** using the Finkelstein reaction of salicylaldehyde **78** with ethyl bromoacetate in the presence of KI as a catalyst under mild reaction conditions (Scheme 14) [49]. Subsequently, compound **79** underwent condensation with K_2_CO_3_ in DMF at 120 °C for 3 h to afford ethyl benzofuran-2-carboxylate **80**, which was then hydrolyzed with sodium hydroxide in DMF at 60 °C for 30 min, resulting in benzo[*b*]furan-2-carboxylic acid **81**. Finally, the esterification reaction of carboxylic acid **81** with shikonin **82** using a mixture of DCC and DMAP in dichloromethane at 0 °C for 8 h afforded shikonin–benzo[*b*]furan hybrids **83a**–**q** in low yields (20–36%).

The antiproliferative activity of the shikonin–benzo[*b*]furan hybrids **83a**–**q** was evaluated against five human cancer cell lines (HepG2, HT29, HCT116, MDA-MB-231, and A549) and two non-cancerous cells (293T and LO2) using the MTT assay, with colchicine, shikonin, and CA-4 as the standard drugs (Table 20). The results demonstrated significant antiproliferative activity for the majority of the hybrids against the tested cancer cell lines. Remarkably, compounds **83c** (R^1^ = 3-OMe), **83o** (R^1^ = 3-OEt), and **83i** (R^1^ = 3-*tert*-butyl) exhibited outstanding activity in the HT29 cell line, with IC_50_ values of 0.18, 0.73, and 0.82 µM, respectively, compared to shikonin and colchicine (IC_50_ = 2.80 and 1.81 µM, respectively). Surprisingly, compound **83c** (IC_50_ = 0.18 µM) demonstrated even higher activity than CA-4 (IC_50_ = 0.31 µM) against the HT29 cell line. The impact of substitutions on antiproliferative activity was evident, as the most active shikonin–benzo[*b*]furan hybrids **83c**, **83i**, and **83o** with a substitution at position 3 of the salicylaldehyde **78** displayed greater potency than shikonin in four of the cancer cell lines tested. However, this trend was observed only in mono-substituted compounds because di-substituted compounds (**83i** and **83p**) exhibited lower activity than them at the same position. On the other hand, compound **83f** (R^1^ = 2-Br) at position 2 demonstrated significant activity comparable to that of shikonin. Furthermore, the cytotoxicity of all compounds was evaluated in two non-cancerous cells (293T and LO2) (Table 20). The results indicated that all shikonin–benzo[*b*]furan hybrids exhibited low cytotoxicity (CC_50_ > 100 µM). These findings suggest their potential as promising candidates for further exploration in cancer treatment research.

Subsequently, the effect of hybrid **83c** on tubulin microtubule dynamics was evaluated in the HT29 cell line, using colchicine, CA-4, and paclitaxel as the standard drugs [49]. The hybrid **83c** exhibited a similar action to colchicine, indicating its potential as a microtubulin destabilizing agent with a more potent inhibitory effect on tubulin polymerization than both colchicine and CA-4, as evidenced by their respective IC_50_ values of 0.98, 2.11, and 1.12 µM (Table 21). In addition, compound **83c** displayed a competitive trend at the tubulin binding site, demonstrating a remarkable 92.42% inhibition at 4 µM, comparable to colchicine.

**Figure 26:**
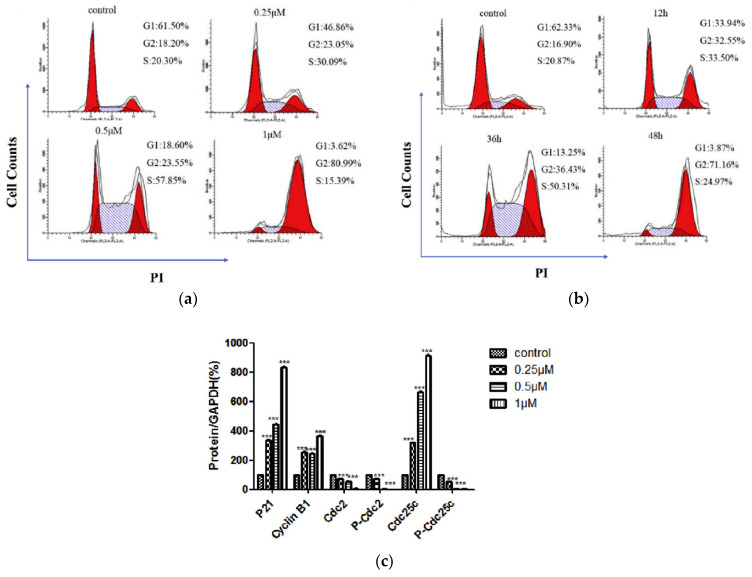
The cell cycle analysis of hybrid **83c**: (**a**) using HT29 cells at concentrations of 0.25, 0.5, and 1 µM; (**b**) with HT29 cells at time points of 12, 36, and 48 h; and (**c**) Western blot showing the expression of cell-cycle-related proteins. Data are means ± SD of three independent experiments. *** *p* < 0.05 vs. control. Reproduced with permission from ref. [49]. Copyright Elsevier Inc., 2023.

The results led to additional biological investigations on hybrid **83c**, including cell cycle and apoptosis assays, tubulin antiangiogenesis, and antivascular assays [49]. Flow cytometry was employed to analyze the cell cycle using the HT29 cell line at varying concentrations of hybrid **83c**. The results revealed cell cycle arrest at the G2/M phase persisting over time (Figure 26a,b). Moreover, a Western blot study was performed to assess the impact of hybrid **83c** on cell-cycle-related proteins. The study revealed that hybrid **83c** increased the expression of P21 and cyclin B1, alongside reducing the expression of Cdc2, p-Cdc2, and p-Cdc25c, corroborating the findings from the cell cycle analysis (Figure 26c).

**Figure 27:**
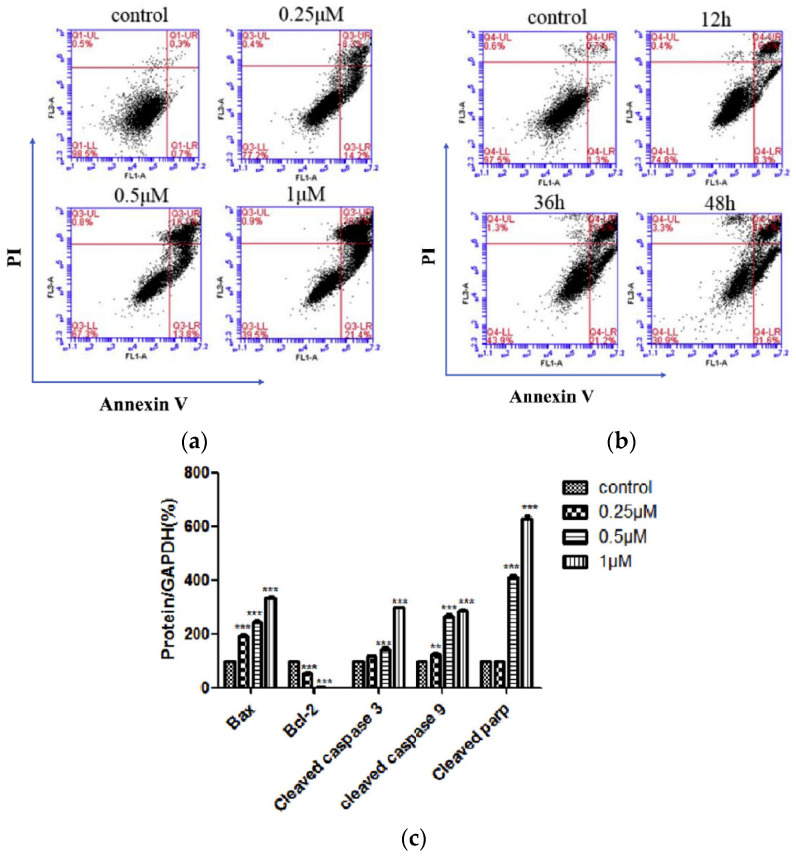
Apoptosis analysis of hybrid **83c**: (**a**) using HT29 cells at concentrations of 0.25, 0.5, and 1 µM; (**b**) with HT29 cells at time points of 12, 36, and 48 h; (**c**) Western blot showing the expression of apoptosis-related proteins. Data are means ± SD of three independent experiments. ** *p* < 0.01; *** *p* < 0.05 vs. control. Reproduced with permission from ref. [49]. Copyright Elsevier Inc., 2023.

Cell apoptosis analysis was performed using the Annexin V-FITC/PI assay and confirmed by the Western blot in HT29 cells. Hybrid **83c** induced cell apoptosis in a concentration- and time-dependent manner (Figure 27a,b) [49]. Moreover, the expression of proteins associated with cell apoptosis, including Bax, PARP, caspase-3, and caspase-9, increased, while Bcl-2 expression decreased, aligning with the observed trend in cell apoptosis (Figure 27c).

**Figure 28:**
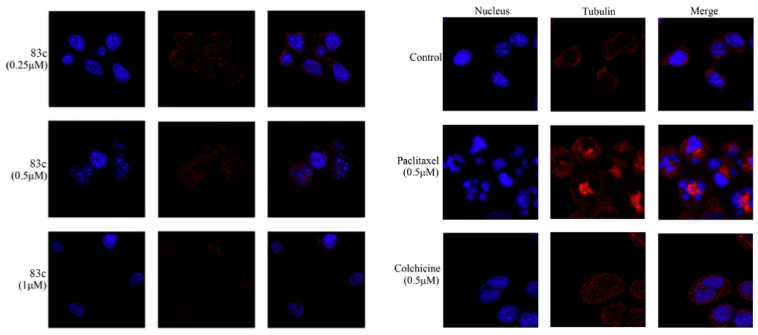
Evaluation of antimicrotubule effects against HT29 cells using confocal fluorescence microscopy for hybrid **83c** (0.25, 0.5, and 1 mM), colchicine (0.5 mM), paclitaxel (0.5 mM), and DMSO. The nucleus was stained with DAPI (blue), and tubulins were labeled with Alexa Fluor 594 (red), captured with a confocal fluorescence microscope. Reproduced with permission from ref. [49]. Copyright Elsevier Inc., 2023.

A complementary study was conducted to explore the effect of hybrid **83c** on inhibiting tubulin polymerization and determine its potential to regulate microtubule dynamics in living cells [49]. The immunofluorescence assay was performed using the HT29 cell line, with colchicine and paclitaxel as positive control drugs (Figure 28). The results demonstrated that cells treated with paclitaxel showed more stable microtubules, whereas those treated with colchicine disintegrated and became soluble. Similarly, cells treated with hybrid **83c** showed a similar response to colchicine, inducing the collapse of microtubules in a dose-dependent manner. On the other hand, the potential antivascular activity of hybrid **83c** was investigated using endothelial cells (HUVECs), which were treated with different concentrations of **83c** and then seeded in Matrigel. After 6 h of treatment, cells treated with hybrid **83c** exhibited a dose-dependent inhibition in the formation of HUVEC cords, suggesting that hybrid **83c** can impede the formation of HUVEC tubes.

**Scheme 15:**
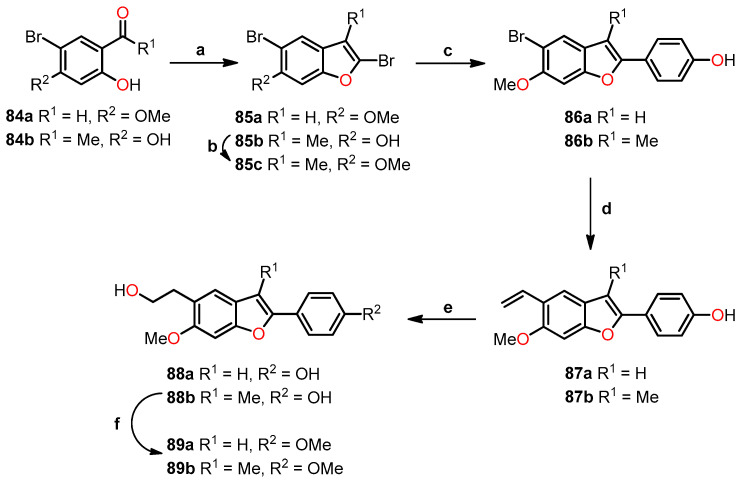
Synthesis of 2-arylbenzo[*b*]furan derivatives **88a**–**b** and **89a**–**b**, and their reaction conditions: (**a**) CBr_4_, TPP, Zn, NH_4_Cl, Cs_2_CO_3_, CuI, CH_3_CN/DCM (4:1), 85 °C, 2 h, 40–60%; (**b**) K_2_CO_3_, MeI, acetone, 60 °C, 1 h, 64%; (**c**) K_2_CO_3,_ 4-(4,4,5,5-tetramethyl-1,3,2-dioxaborolan-2-yl)phenol, Pd(PPh_3_)_4_, THF/EtOH/H_2_O (20:1:1), 80 °C, 16 h, 50–60%; (**d**) K_3_PO_4_, vinylboronic acid, Pd(dppf)Cl_2_.CH_2_Cl_2_, DMF/H_2_O (4:1), 90 °C, 2 h, 81–88%; (**e**) BH_3_/THF, 2M NaOH–H_2_O_2_, 0 °C, r.t, 2 h, 53–68%; (**f**) K_2_CO_3_, MeI, acetone, 60 °C, 1 h, 72%.

**Scheme 16:**
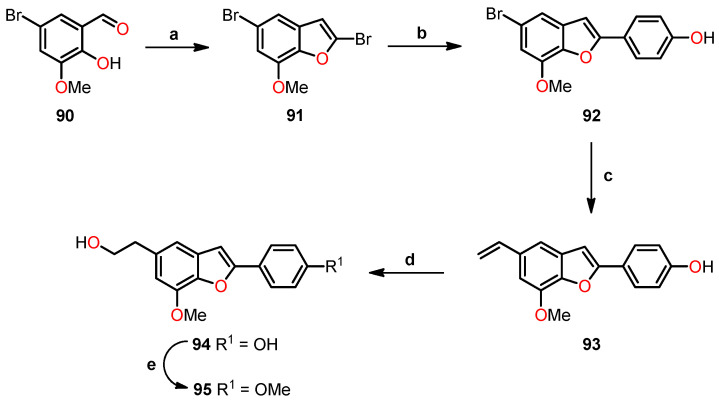
Synthesis of 2-arylbenzo[*b*]furan derivatives **94** and **95** and their reaction conditions: (**a**) CBr_4_, TPP, Zn, NH_4_Cl, Cs_2_CO_3_, CuI, CH_3_CN/DCM (4:1), 85 °C, 72%; (**b**) K_2_CO_3_, 4-(4,4,5,5-tetramethyl-1,3,2-dioxaborolan-2-yl)phenol, Pd(PPh_3_)_4_, THF/EtOH/H_2_O (20:1:1), 80 °C, 16 h, 57%; (**c**) K_3_PO_4_, vinylboronic acid, Pd(dppf)Cl_2_.CH_2_Cl_2_, DMF/H_2_O (4:1), 90 °C, 2 h, 90%; (**d**) BH_3_/THF, 2 M NaOH–H_2_O_2_, 0 °C, r.t, 2 h, 60%; (**e**) K_2_CO_3_, MeI, acetone, 60 °C, 1 h, 70%.

**Scheme 17:**
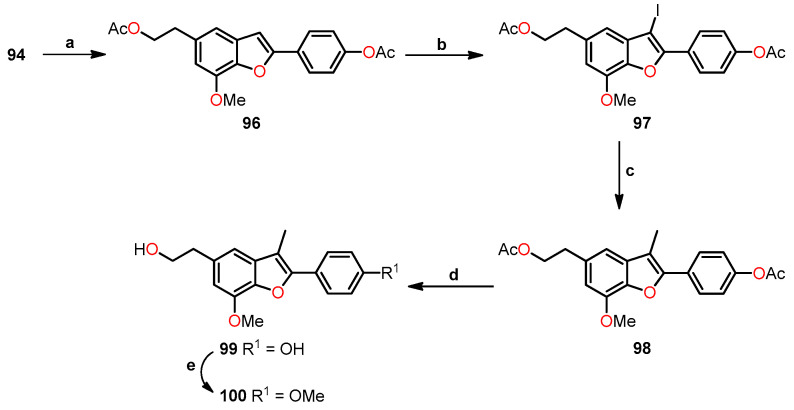
Synthesis of 2-arylbenzo[*b*]furan derivatives **99** and **100** and their reaction conditions: (**a**) AcCl, DMAP, Et_3_N, DCM, 0 °C, r.t, 2 h, 88%; (**b**) NIS, PTSA, CH_3_CN, 0 °C, r.t, 2 h, 80%; (**c**) K_3_PO_4_, boronic acid, Pd(dppf)Cl_2_.CH_2_Cl_2_, 1,4-dioxane/H_2_O (100:1), 110 °C, 2 h, MWI, 73%; (**d**) LiOH/H_2_O, THF/EtO/H_2_O (4:4:1), 0 °C, r.t, 2 h, 89%; (**e**) K_2_CO_3_, MeI, acetone, 60 °C, 1 h, 76%.

As mentioned earlier, aryl- and alkylbenzo[*b*]furan groups have exhibited significant biological activity against various types of human cancer. Building upon these promising findings, Sivaraman et al. undertook the synthesis of a series of 2-aryl[*b*]benzofurans, classified as lignane and neolignane, through a one-pot reaction using 2-bromobenzo[*b*]furans as crucial intermediates [50]. *Lavandula agustifola* served as the source for both natural (**89a**, **99**, and **100**) and non-natural (**88a**–**b**, **89b**, **94**, and **95**) compounds in the process (Scheme 15, Scheme 16 and Scheme 17).

In the initial synthesis, 2,5-dibromo-6-methoxybenzo[*b*]furan **85a** was obtained in a 60% yield through a *gem*-dibromo olefination/cyclization sequence (Scheme 15). Subsequently, a selective C–2 acylation of the Suzuki-type reaction resulted in the formation of 4-(5-bromo-6-methoxybenzofuran-2-yl)phenol **86a**. Further vinylation of **86a**, followed by a hydroboration/oxidation sequence, led to the compound **88a**, which was then subjected to methylation to afford compound **89a**. For the tetrasubstituted benzo[*b*]furans **88b** and **89b**, a similar reaction pathway was employed, utilizing 1-(5-bromo-2,4-dihydroxyphenyl)ethanone **84b**, leading to the formation of 2,5-dibromo-3-methylbenzo[*b*]furan-6-ol **85b**. Subsequent methylation of **85b** gave 2,5-dibromo-6-methoxy-3-methylbenzo[*b*]furan **85c**. By employing the identical reaction steps as previously described, compounds **88b** and **89b** were obtained with overall yields of 6% and 5%, respectively.

The second reaction was performed following a similar procedure as described in Scheme 15. Initially, a one-pot cyclization of 5-bromo-2-hydroxy-3-methoxybenzaldehyde **90** yielded benzo[*b*]furan **91**. Subsequently, a selective C–2 arylation gave **92**, and vinylation led to the formation of 4-(7-methoxy-5-vinyl-benzofuran-2-yl)phenol **93** (Scheme 16). Afterward, a hydroboration/oxidation sequence afforded compound **94**, and its methylation yielded compound **95**, with overall yields of 22% and 16%, respectively.

Benzo[*b*]furan derivatives **99** and **100** were synthesized by a multi-step process starting from **94**. Initially, compound **94** underwent diacetylation to afford **96** (Scheme 17). Subsequently, a regioselective iodination reaction of **96** with NIS afforded iodinated compound **97**, which underwent a Suzuki reaction to give compound **98**. Finally, benzofuran **98** suffered deprotection and methylation to obtain compounds **99** and **100** with overall yields of 10% and 8%, respectively.

**Figure 29:**
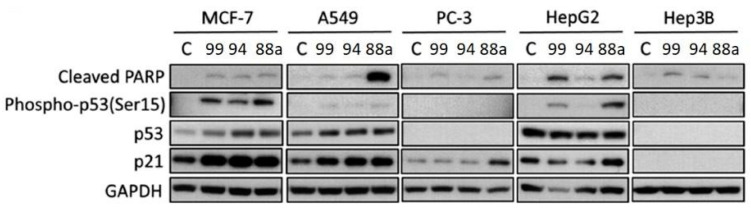
Western blot analysis for PARP, Phospho-p53, p53, p21, and GAPDH as the loading control for compounds **88a**, **94**, and **99**. Reprinted (adapted) with permission from ref. [50]. Copyright American Chemical Society, 2023.

The previously synthesized compounds were evaluated for their cytotoxic effects on five human cancer cell lines, including MCF-7, A549, PC3, HepG2, and Hep3B, utilizing the MTT assay [50]. Notably, compounds **88a**, **94**, and **99** exhibited significant reductions in cell counts, indicating promising cytotoxic activity. Subsequently, comprehensive biological studies were conducted using Western blot analysis to assess the effects of compounds **88a**, **94**, and **99** on proteins associated in cellular processes (Figure 29). As expected, all compounds induced the cleavage of PARP, indicative of activation of the apoptosis pathway. Additionally, MCF-7, A549, and HepG2 cells showed phosphorylation and stabilization of p53 in response to the compounds, while PC3 and Hep3B cells did not exhibit this response. Furthermore, the compounds similarly induced the p21 target gene across all cancer cell lines, regardless of the sensitivity of each cell line. This suggests that these compounds affect cancer cell survival through a combination of both p53-dependent and p53-independent mechanisms.

**Scheme 18:**
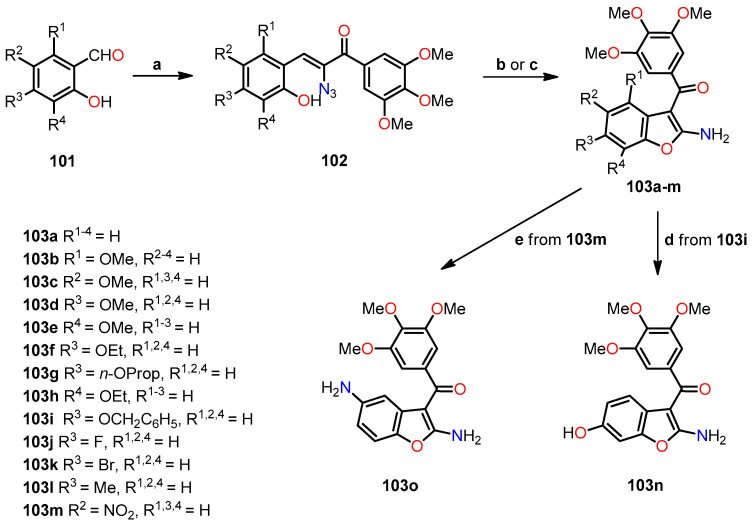
Synthesis of 2-amino-3-(3′,4′,5′-trimethoxybenzoyl)benzo[*b*]furan derivatives **103a**–**o**, and the reaction conditions: (**a**) 2-azido-1-(3,4,5-trimethoxyphenyl)ethanone, piperidinium acetate, MeOH, r.t, 24 h; (**b**) PTSA (20 mol%), CH_3_CN, reflux, 12 h; (**c**) white CFL (25 W), PTSA (20 mol%), CH_3_CN, r.t, 24 h; (**d**) Pd/C (10%), EtOH, 40 bar, 60 °C, 30 min; (**e**) Fe, NH_4_Cl, EtOH/H_2_O, reflux, 3 h.

Similarly, Oliva et al. synthesized a novel series of 2-amino-3-(3′,4′,5′-trimethoxybenzoyl)benzo[*b*]furan derivatives **103a**–**o** and evaluated their in vivo and in vitro anticancer activity [51]. The synthesis involved two distinct reaction steps, as illustrated in Scheme 18. Initially, the Knoevenagel condensation reaction of salicylaldehyde **101** with 2-azido-1-(3,4,5-trimethoxyphenyl)ethanone was conducted in methanol at room temperature for 24 h, utilizing piperidinium acetate to afford *α*-azido chalcone **102**. Subsequently, the chalcones were treated with PTSA (20 mol%) in refluxing acetonitrile for 12 h, resulting in the formation of 2-amino-3-(3′,4′,5′-trimethoxybenzoyl)benzo[*b*]furan derivatives **103a**–**n** in 60–80% yields. An alternative photochemical process was also employed, where thermal heating was replaced with irradiation at room temperature using a 25 W compact fluorescent lamp (CFL) to obtain compounds **103a**–**n**, albeit in reduced yields. Moreover, compound **103n** was obtained from **103i** through hydrogenation using Pd/C (10%) as the catalyst, while compound **103o** was derived from **103m** through reduction using iron and ammonium chloride. 

The antiproliferative activity of the compounds **103a**–**l** and **103n**–**o** was evaluated against six human cancer cell lines (HeLa, HT-29, Daoy, HL-60, SEM, and Jurkat) using the MTT assay and CA-4 as the standard drug (Table 22). The results revealed the remarkable activity of four of the compounds (**103d**, **103f**, **103k**, and **103l**), with IC_50_ values below 5 nM. Specifically, compound **103f** (R^1,2,4^ = H, R^3^ = OEt) displayed the highest potency with an IC_50_ value of 5 pM against the Daoy cell line, outperforming the other cell lines evaluated. Both compounds **103f** and **103l** (R^1,2,4^ = H, R^3^ = Me) exhibited greater potency than CA-4 in all cancer lines and demonstrated significantly higher sensitivity in Daoy, HL-60, and Jurkat cell lines, with IC_50_ values ranging from 0.005 to 0.38 nM. Furthermore, a clear and consistent trend in the antiproliferative activity was observed among the methoxy-substituted compounds (**103b**–**e**). The compounds bearing a methoxy group at either the C–5 or C–6 position exhibited the highest activity, while those with the methoxy group at the C–4 or C–7 position displayed the lowest activity. Specifically, the order of potency was as follows: 6-OMe (**103d**) > 5-OMe (**103c**) > 7-OMe (**103e**) > 4-OMe (**103b**). In particular, compound **103b** exhibited a remarkable IC_50_ value ranging from 2.8 to 8.5 nM. Moreover, the substitution of the methoxy group with a methyl group at C–6 (**103d** and **103l**, respectively) resulted in a 3–14-fold increase in activity against HeLa, HT-9, SEM, and Jurkat cell lines for **103l** compared to **103d**. However, the substitution of the methoxy group with a hydroxy group at C–6 (**103n**) did not enhance its activity in any significant manner. Finally, in the halide compounds, increasing the size from fluorine to bromine (**103k**) led to a 71–338-fold increase in activity across all six cell lines, with particularly pronounced effects observed in the Daoy line.

The antiproliferative studies facilitated the identification of the most promising compounds from the synthesized series of 2-amino-3-(3′,4′,5′-trimethoxybenzoyl)benzo[*b*]furans [51]. Subsequently, compounds **103c**–**d**, **103f**–**g**, and **103k**–**l** were selected for further evaluation to assess their inhibitory effects on tubulin polymerization and [^3^H]colchicine binding to tubulin (Table 23). The results demonstrated that all compounds exhibited comparable tubulin polymerization inhibition as compared to CA-4. Specifically, compounds **103f** and **103l** displayed greater potency than CA-4 (IC_50_ = 0.54 nM), with IC_50_ values of 0.37 and 0.39 nM, respectively. Moreover, in the colchicine binding studies to tubulin, compounds **103d**, **103f**, and **103l** showed results similar to CA-4 at concentrations of 5 and 0.5 µM. These findings suggest that the compounds in this assay are robust antiproliferative and antitubulin agents.

**Figure 30:**
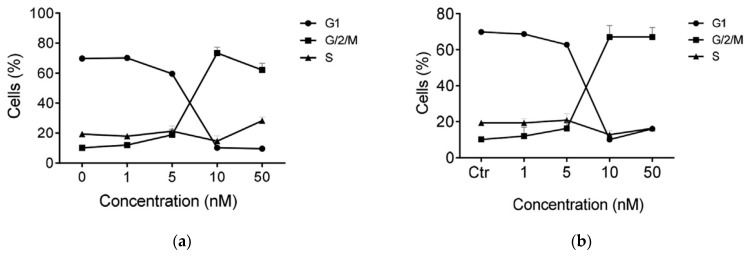
The cell cycle distribution in the HeLa cell line using flow cytometry analysis of compounds (**a**) **103f** and (**b**) **103l**. Reproduced with permission from ref. [51]. Copyright Elsevier Inc., 2023.

The potent inhibition of tubulin polymerization displayed by compounds **103f** and **103l** prompted the evaluation of their effects on cell cycle progression using flow cytometry with the HeLa cell line. As shown in Figure 30a,b, both compounds caused a remarkable cell cycle arrest at the G2/M phase after 24 h of treatment at a concentration of 10 nM. Furthermore, there was a notable reduction in the number of cells in the G1 phase, while no significant effect was observed on the S phase for both compounds. These findings indicate that compounds **103f** and **103l** exhibited a strong influence on cell cycle dynamics, which may play a crucial role in their antiproliferative activity.

**Figure 31:**
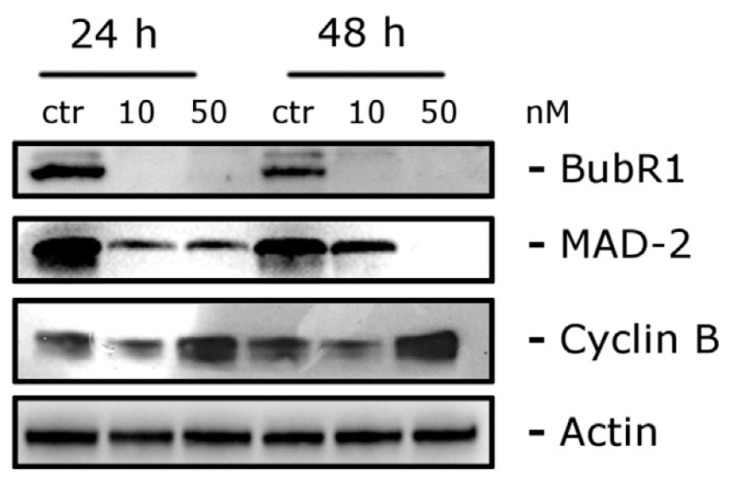
Analysis of the effect of compound **103f** on the regulation of cell-cycle-related protein levels. Reproduced with permission from ref. [51]. Copyright Elsevier Inc., 2023.

Furthermore, a study was conducted to investigate the effects of compound **103f** on two proteins, Bubr1 and Mad-2, which play essential roles in the spindle assembly checkpoint (SAC), and are associated with apoptotic cell death (Figure 31). The treatment with compound **103f** resulted in a significant reduction in the expression of both Bubr1 and Mad-2 proteins, even at low concentrations as low as 10 nM, indicating a potential arrest of the mitotic checkpoint. Moreover, the study examined cyclin B, a key regulator in the G2/M phase of the cell cycle, which showed a dose-dependent increase in expression in response to compound **103f**. This result aligns with the rapid accumulation of cells in the G2/M phase induced by the compound.

Considering the well-established association of tubulin-targeting agents with antivascular effects against tumor endothelium, the response of compound **103f** was evaluated to assess its antivascular activity in angiogenesis in vivo using HUVEC endothelial cells [51]. For this purpose, HUVECs were seeded on Matrigel to analyze the impact of compound **103f** on the formation of “tubule-like” structures in these cells. The results indicated that after 1 h of treatment, compound **103f** effectively disrupted the HUVEC network at both concentrations tested (10 and 100 nM) compared to the control cells. To quantitatively evaluate these effects, image analysis was conducted to measure parameters such as the tubule segment length, meshwork area, and number of branches. Notably, the results showed a statistically significant effect at a concentration of 10 nM on segment length and mesh area, underscoring the strong potential of compound **103f** to induce vascular disruption. These findings strongly suggest that compound **103f** holds promise as a potential antivascular agent for inhibiting angiogenesis.

**Figure 32:**
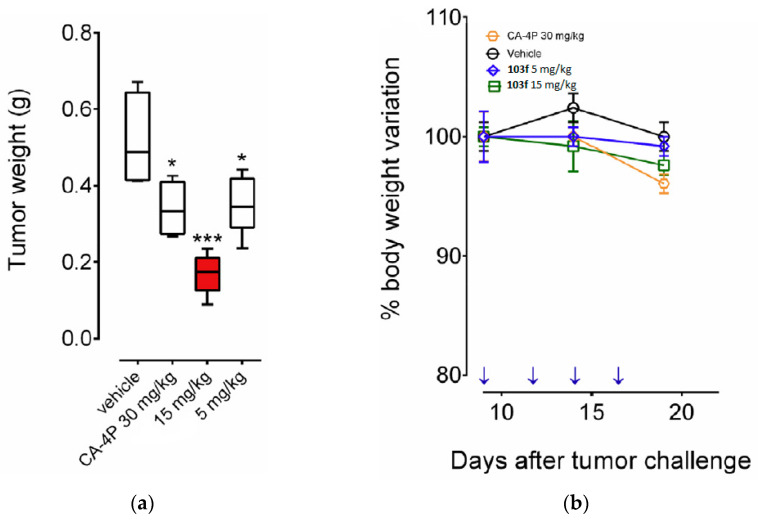
Suppression of mammary tumor growth in female C57BL/6 mice by compound **103f**: (**a**) tumor weight measurement at the end of the procedure (*n* = 5 mice/group) and (**b**) changes in mouse body weight during the procedure. Asterisks indicate significant difference between the treated and the control group. * *p* < 0.05, *** *p* < 0.0001. Reproduced with permission from ref. [51]. Copyright Elsevier Inc., 2023.

Finally, compound **103f** underwent in vivo tests to evaluate its antitumor and cytotoxic effects in syngeneic mice. The method involved injecting E0771 murine breast cancer cells into the mammary fat pads of female C57BL/6 mice. Simultaneously, compound **103f** was administered intraperitoneally on alternate days at two doses (5 or 15 mg/kg). The results demonstrated a dose-dependent reduction in tumor growth upon treatment with compound **103f**, achieving a decrease of 45.7% and 16.9% at 15 and 5 mg/kg, respectively (Figure 32a). Compound **103f** exhibited higher potency than the reference drug (CA-4P), which reduced tumor growth by 26.5% at 30 mg/kg. Importantly, cytotoxicity tests revealed no apparent signs of toxicity at the 15 mg/kg doses of compound **103f** (Figure 32b). These findings highlight the potential of compound **103f** as a promising antitumor agent with limited toxicity in vivo.

**Scheme 19:**
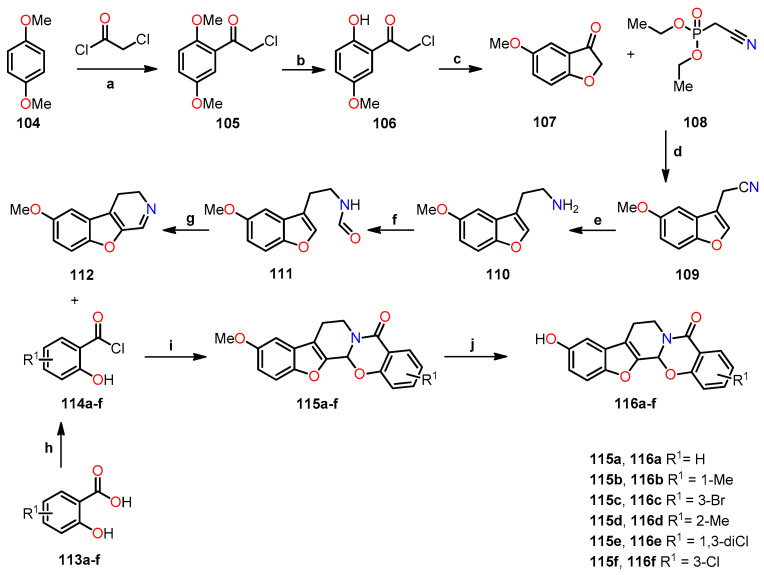
Synthesis of evodiamine derivatives **115a**–**f** and **116a**–**f**, and reagents and reaction conditions: (**a**) AlCl_3_, DCM, r.t, 24 h, 90%; (**b**) AlCl_3_, DCM, r.t, 12 h, 88%; (**c**) AcONa, EtOH, reflux, 2 h, 91%; (**d**) THF, NaH, r.t, 12 h, 75%; (**e**) BH_3_·THF, reflux, 3 h; (**f**) Ethyl formate, reflux, 12 h, 72%; (**g**) POCl_3_, DCM, r.t, 12 h, 76%; (**h**) SOCl_2_, reflux, 2 h; (**i**) DCM, r.t, 12 h, 78–82%; (**j**) BBr_3_, DCM, −78 °C, 6 h, 82–91%.

In 2021, Xu et al. accomplished the successful synthesis of novel polycyclic heterocycles derived from Evodiamine, a quinazolinocarboline alkaloid naturally occurring in the *Evodia rutaecarpa* plant native to China [52]. This research primarily aimed to evaluate these compounds as potential inhibitors of topoisomerase I (Top 1) for treating triple-negative breast cancer (TNBC), an aggressive subtype of breast cancer. The key focus of their synthetic efforts was the preparation of the 2-(5-methoxybenzofuran-3-yl)ethanamine **110**, which involved a five-step reaction sequence using 1,4-dimethoxybenzene **104** as the starting reagent (Scheme 19). Subsequently, intermediate **110** underwent an amidation reaction with ethyl formate in reflux conditions for 12 h to afford amide **111** in a 72% yield. The following step involved an intramolecular cyclization of compound **111** using POCl_3_ in dichloromethane at room temperature for 12 h to give compound **112**, which was then reacted with substituted salicylic acid chlorides **114a**–**f** in dichloromethane at room temperature for 12 h to obtain the compounds **115a**–**f** in good yields. Finally, the intermediates **115a**–**f** underwent *O*-demethylation using BBr_3_ in dichloromethane at −78 °C for 6 h to furnish evodiamine derivatives **116a**–**f** in good yields.

The antiproliferative evaluation of evodiamine analogs **115a**–**f** and **116a**–**f** was performed on MDA-MB-435 human breast carcinoma cells using the MTT method, with evodiamine as the standard drug (Table 24) [52]. Notably, the results significantly favored compounds **116a**–**f**, exhibiting a higher percentage of inhibition at 10 µM compared to evodiamine. Building upon these promising findings, compounds **116a**–**f** underwent further evaluation against four human cancer cell lines: MDA-MB-435, MDA-MB-231, HCT116, and A549, using the MTT method in the presence of evodiamine and camptothecin as the standard drugs (Table 25). The breast cancer lines showed the highest sensitivity to these compounds. For instance, compounds **116a** (R^1^ = H) and **116f** (R^1^ = 3-Cl) showed the best activity against the MDA-MB-435 cell line, with IC_50_ values of 0.47 and 0.42 µM, respectively, which were comparable to the camptothecin (IC_50_ = 0.31 µM). The introduction of a halogen at position 3 significantly enhanced the antiproliferative activity of the analogs, as demonstrated by **116f**, which was the most active in the series with IC_50_ values of 0.36, 0.42, and 0.76 µM against the MDA-MB-231, MDA-MB-435, and HCT116 cell lines, respectively.

**Figure 33:**
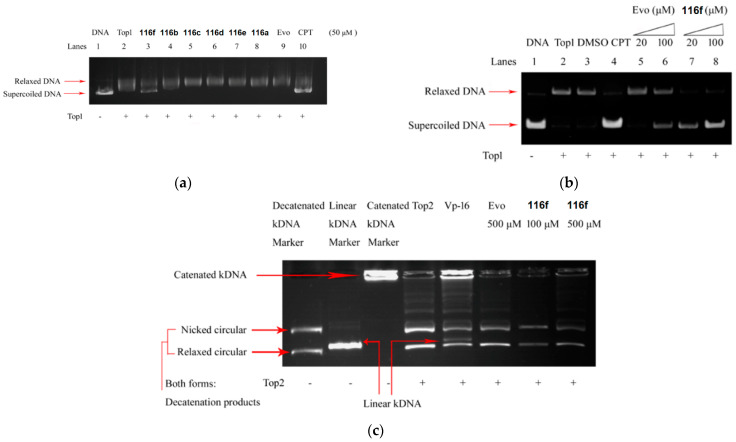
(**a**) Inhibition of Top 1 DNA relaxation activity in compounds **116a**–**f**, (**b**) inhibition of Top 1 relaxation in evodiamine (evo) and compound **116f**, (**c**) catalytic inhibition of Top 2 or a Top 2 poison using a kDNA substrate in compound **116f**. Reprinted (adapted) with permission from ref. [52]. Copyright American Chemical Society, 2023.

The data presented above shed light on the main objective of this study, which involves evaluating the topoisomerase inhibitory activity of compounds **116a**–**f** [52]. To achieve this, a Top-1-mediated DNA cleavage assay was performed using purified Top 1 on compounds **116a**–**f**. During the assay, DNA and Top 1 were incubated with or without these compounds to observe their effect on the appearance of relaxed DNA fragments. Remarkably, among the tested compounds, only **116f** and camptothecin (CPT) exhibited significant inhibition of Top-1-mediated relaxation of supercoiled DNA at a concentration of 50 µM (Figure 33a). Furthermore, in a Top 1 inhibition study, compound **116f** showed activity at 20 µM, while evodiamine only displayed moderate activity up to 500 µM (Figure 33b,c). These findings strongly suggest that **116f** specifically targets Top 1 and holds great potential as a promising candidate for further investigation in cancer drug development.

**Figure 34:**
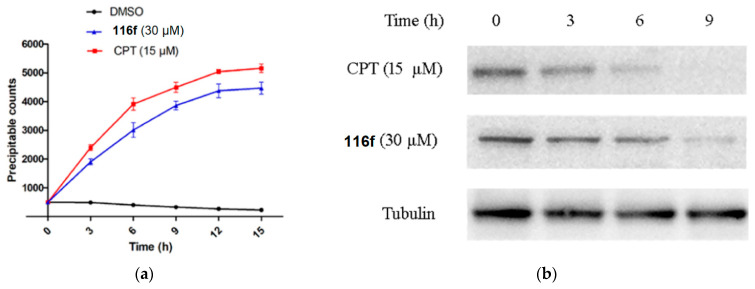
Formation of cleavable complexes of Top 1–DNA induced by compound **116f**: (**a**) [^3^H]thymidine labeling of the MDA-MB-231 cell line over different time intervals, (**b**) immunological band assay for Top 1 in MDA-MB-231 across varied time periods. Reprinted (adapted) with permission from ref. [52]. Copyright American Chemical Society, 2023.

Complementary biochemical studies were performed to explore the ability of the compound **116f** to effectively trap Top 1–DNA cleavable complexes within cancer cells, potentially leading to cell death. To assess this, the researchers quantified the number of trapped cleavable complexes using [^3^H]thymidine incorporation and SDS-K^+^ precipitation methods. As shown in Figure 34a, there was a significant increase in the formation of the cleavable complex over prolonged periods in cells treated with both camptothecin and the compound **116f**. Furthermore, to confirm the formation of the cleavable complex, an immunological band depletion assay was performed to verify the presence of Top 1 in the precipitated complex, demonstrating that the complex could not migrate through the gel. Conversely, in the absence of the complex, Top 1 would have migrated through the gel. The results revealed a proportional decrease in the amount of free Top 1 with increasing time in cells treated with 15 µM of CPT and 30 µM of **116f**, particularly evident after 9 h, where almost no free Top 1 was detected (Figure 34b). These compelling findings strongly support the notion that the **116f** analog effectively interacts with Top 1, leading to the formation of the cleavable complex, underscoring its potential as a promising candidate for anti-cancer drug development.

**Figure 35:**
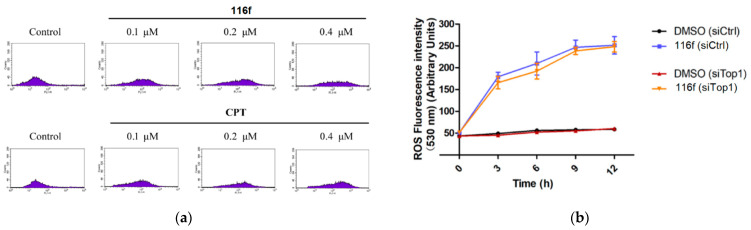
Generation of ROS by compound **116f** independent of Top 1: (**a**) flow cytometry analysis of ROS generation in MDA-MB-231 cells treated with **116f**, and (**b**) comparison of ROS generation in parental MDA-MB-231 cells, MDA-MB-231 cells with control siRNA (siRNA-Ctrl), and Top-1-deficient MDA-MB-231 cells (siRNA-Top 1). Reprinted (adapted) with permission from ref. [52]. Copyright American Chemical Society, 2023.

Furthermore, a comprehensive study was performed to investigate the potential of stabilizing Top 1–DNA covalent complexes through an indirect process involving ROS generation and subsequent oxidative DNA damage, as observed in other studies with staurosporine. This investigation employed a fluorogenic ROS probe in combination with flow cytometry analysis. As demonstrated in Figure 35a, the treatment of MDA-MB-231 cells with the compound **116f** resulted in a compelling, dose-dependent increase in ROS levels. Notably, even in the absence of Top 1 in MDA-MB-231 cells (siRNA-Top 1), the **116f** analog induced ROS generation, indicating that the compound has the ability to generate ROS independently of Top 1 (Figure 35b).

**Figure 36:**
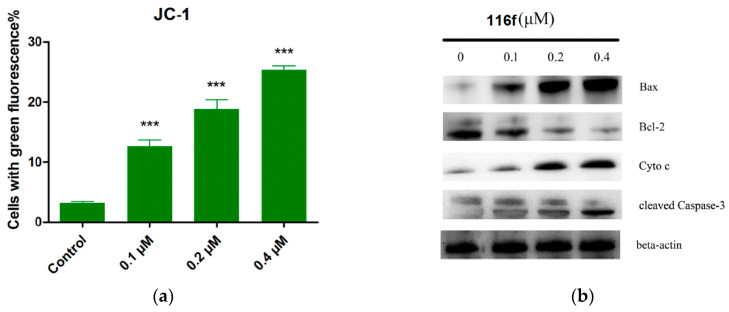
Effect of compound **116f** on (**a**) mitochondrial dysfunction using JC-1 staining and (**b**) expression levels of related proteins analyzed by Western blot in the MDA-MB-231 cell line. *β*-Actin was used as an internal control. *** *p* < 0.001. Reprinted (adapted) with permission from ref. [52]. Copyright American Chemical Society, 2023.

The cell cycle study focused on evaluating the mitochondrial dysfunction involved in the apoptosis process. To achieve this, a JC-1 fluorescent probe was employed to measure the mitochondrial membrane potential, incubated with MDA-MB-231 cells at different concentrations, and quantified by flow cytometry analysis. Mitochondrial dysfunction was found at 3.0% with 0 µM, 12.2% with 0.1 µM, 18.5% with 0.2 µM, and 25.0% with 0.4 µM of cells after treatment with the compound **116f** (Figure 36a). These data suggest a direct relationship with the mitochondrial pathway. Therefore, it was also necessary to evaluate the expression of apoptotic proteins, including Bax, Bcl-2, cytochrome C, and caspase-3, using a Western blot assay. Figure 36b shows that following 48 h of treatment with **116f**, the levels of Bax, cytochrome C, and caspase-3 proteins noticeably increased, while Bcl-2 expression significantly decreased.

**Figure 37:**
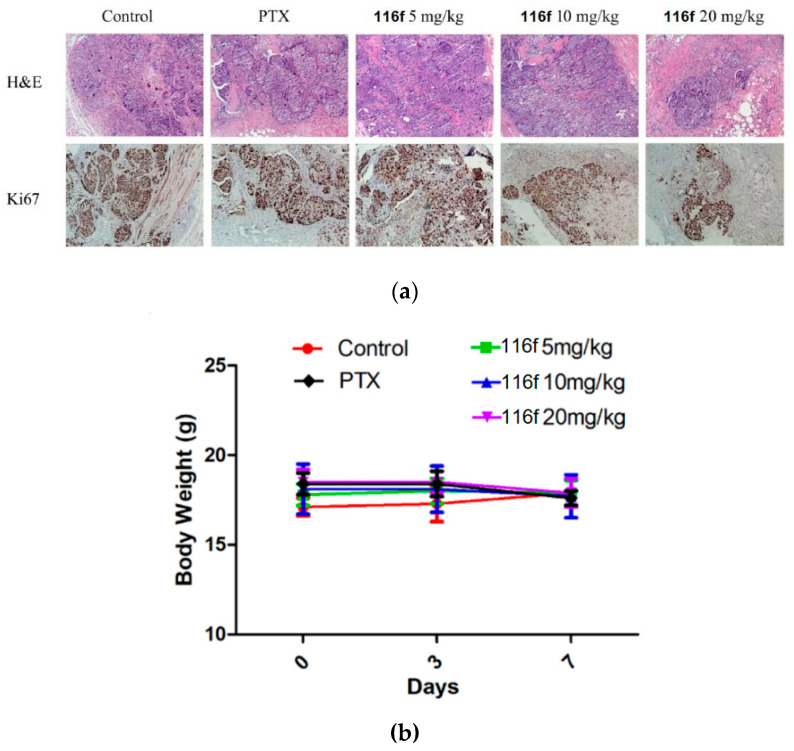
In vivo study of **116f** in a PDTX model: (**a**) immunohistochemical analysis with H&E staining and Ki67 expression in TNBC cells, (**b**) effects of **116f** on mice body weight during treatment. Reprinted (adapted) with permission from ref. [52]. Copyright American Chemical Society, 2023.

Finally, in vivo studies were conducted using a mouse xenograft model with surgical residual tumor samples from a patient with TNBC to assess the anticancer potency of compound **116f** in TNBC, with paclitaxel (PTX) used as the positive control. Different concentrations of **116f** were administered to the mice, followed by histological analysis of tumor tissue sections using H&E staining (Figure 37a). The results showed that the tumors from mice treated with **116f** exhibited reduced cell density and increased necrosis rates compared to the untreated mice. Furthermore, immunofluorescence labeling of cell proliferation marker Ki67 showed a significant decrease in proliferating Ki67 cells in tumors treated with **116f** compared to untreated tumors. This result convincingly demonstrates the potent inhibitory effect of **116f** on tumor growth. Additionally, the body weight of the evaluated mice treated with compound **116f** showed no significant changes, indicating the absence of apparent toxicity (Figure 37b). These promising findings highlight the potential of compound **116f** for future TNBC treatments, even at doses as low as 20 mg/kg.

Using a similar approach, Zhang et al. successfully identified potential drugs for triple-negative breast cancer (TNBC) [53]. They focused on the derivatives of ZINCO3830212, which was selected through molecular docking studies involving pocket U of SIRT3, encompassing crucial amino acids, such as Phe157, Arg158, Ser159, Pro176, Glu177, and Glu323. This particular pocket is known for its significant role in modulating autophagy and its associations with various human cancers. To synthesize these derivatives, the researchers utilized alkanes as linkers and introduced halogen substituents to obtain compounds **117a**–**r** through an *O*-alkylation reaction. Subsequently, all synthesized compounds **117a**–**r** were evaluated against four human cancer cell lines: HL60, U937, MCF-7, and MDA-MB-231, using the MTT method and ZINCO3830212 as the reference compound (Table 26). Among these derivatives, compounds **117c**–**e** exhibited the most promising results in activating SIRT3. The researchers also investigated the impact of alkane length on SIRT3 activation by varying the linker length. Interestingly, they observed a decrease in activity for compound **117o** (*n* = 0, R^1^ = I, R^2^ = 1-pyrrolidinyl) with a shorter linker. Moreover, when substituents other than iodine (i.e., H, Cl, Br, and Me) were used, the activity was not favored in compounds **117n** (*n* = 0, R^1^ = H, R^2^ = 1-pyrrolidinyl) and **117p**–**r** (*n* = 1, R^1^ = Cl, Br, Me, R^2^ = 1-pyrrolidinyl). However, in the case of compound **117c** (*n* = 1, R^1^ = I, R^2^ = 1-pyrrolidinyl) containing iodine, its activity surpassed that of compounds **117p**–**r**. Remarkably, compound **117c** showed the highest antiproliferative activity against the breast carcinoma line MDA-MB-231, with an IC_50_ value of 2.19 µM, compared to ZINCO3830212 (IC_50_ = 33.43 µM). This significant finding highlights its potential as a promising SIRT3 activator for TNBC treatment (Table 26).

**Figure 38:**
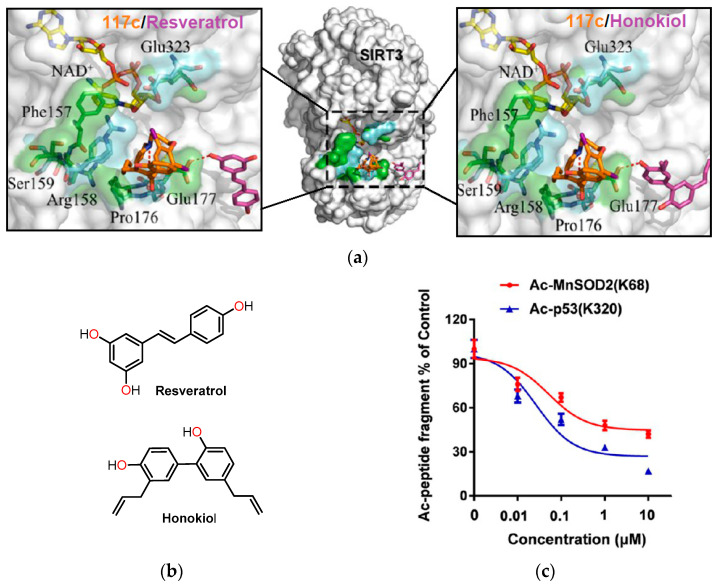
Effect of compound **117c** on SIRT3 activation. (**a**) The molecular coupling of compound **117c** with resveratrol and honokiol in SIRT3. (**b**) The chemical structures of resveratrol and honokiol. (**c**) SIRT3-dependent substrate deacetylation with compound **117c**. Reprinted (adapted) with permission from ref. [53]. Copyright American Chemical Society, 2023.

This study compared the deacetylation activity of the compound **117c** with resveratrol and honokiol, known SIRT3 activators, using molecular coupling and evaluating their antiproliferative effects [53]. Molecular docking revealed that, unlike the compound **117c**, resveratrol and honokiol tended to bind to the acetylated substrate recognition site, indicating no allosteric effect (Figure 38a). Also, compound **117c** showed significantly higher potency than the reference activators, with an IC_50_ value of 2.19 µM against the MDA-MB-231 cell line. In contrast, resveratrol and honokiol showed IC_50_ values of 98.89 µM and 44.89 µM, respectively, with normalized E_max_ values of 0.25 for resveratrol, 0.91 for honokiol, and 1.00 for compound **117c** (Table 27). Additionally, compound **117c** induced the deacetylation of two tested SIRT3 substrates, MnSOD2 and p53 (Figure 38b), further confirming its potent deacetylation and antiproliferative effects.

**Figure 39:**
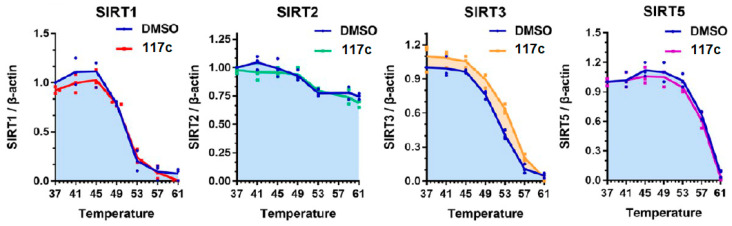
Selectivity of compound **117c** binding to SIRT3 in MDA-MB-231 cell line. Reprinted (adapted) with permission from ref. [53]. Copyright American Chemical Society, 2023.

**Figure 40:**
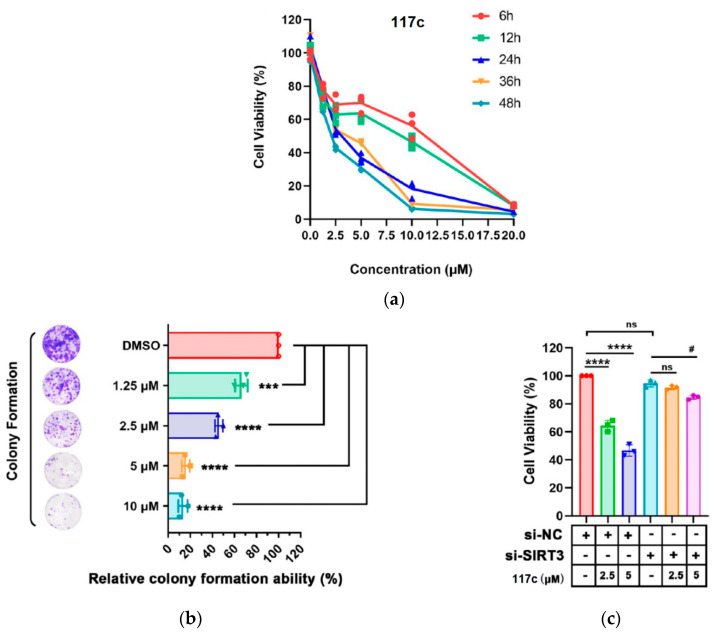
Inhibition of MDA-MB-231 tumor cell proliferation after treatment with compound **117c**. (**a**) MTT cell viability assay for 6–48 h with **117c**. (**b**) Colony formation assay with concentrations ranging from 1.25 to 10 µM. *** *p* < 0.001 and **** *p* < 0.0001 compared with the control group. (**c**) Transfection of MDA-MB-231 cells with siRNA SIRT3 and treated with **117c** at 2.5 and 5 µM. ns means no significance, **** *p* < 0.0001 compared with the si-NC group, and ^#^ *p* < 0.05 compared with the si-SIRT3 group. Reprinted (adapted) with permission from ref. [53]. Copyright American Chemical Society, 2023.

The conducted studies provided significant insights, demonstrating the activation capability of compound **117c** on SIRT3 [53]. To assess its selectivity toward SIRT3, a CETSA cellular thermal shift assay was performed on all SIRTs (SIRT1, SIRT2, SIRT3, and SIRT5). The collected data clearly revealed a direct interaction between compound **117c** and SIRT3, while SIRT1, SIRT2, and SIRT5 remained unaffected in their thermal stability. This distinct result confirmed the specific binding affinity of compound **117c** for SIRT3 in the MDA-MB-231 cell line (Figure 39). With its selectivity established, the researchers explored the influence of SIRT3 on both short- and long-term effects of compound **117c** in the MDA-MB-231 cell line, confirming a concentration- and time-dependent inhibition of tumor cell proliferation (Figure 40a,b). Next, the antiproliferative activity was examined in the presence of SIRT3, and the findings indicated a significant impairment in the inhibitory effect of compound **117c** when SIRT3 was absent. As a result, the antiproliferative effect displayed a marked reduction after SIRT3 knockdown, firmly establishing the dependence of compound **117c** on SIRT3 for its activity (Figure 40c).

**Figure 41:**
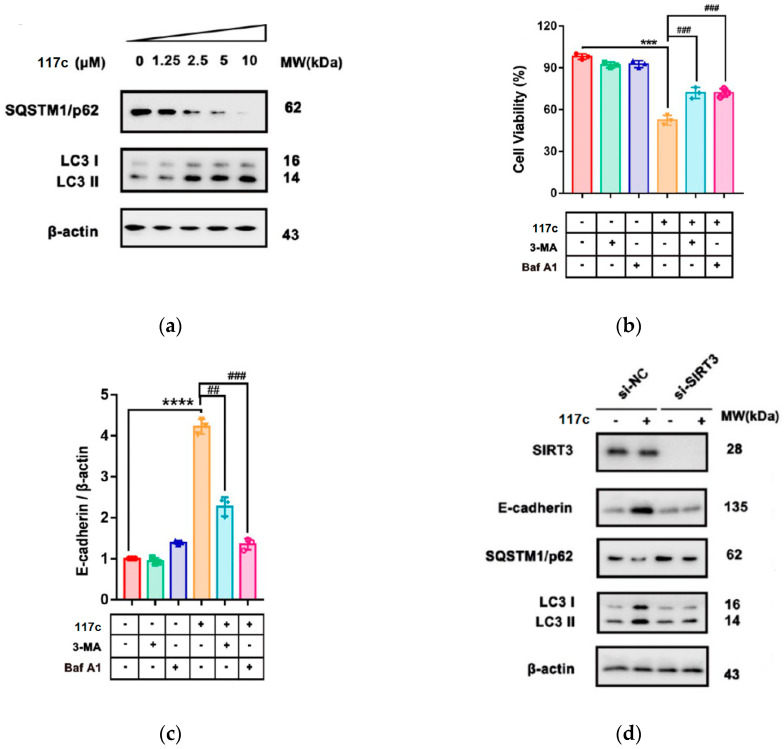
Effect of compound **117c** on autophagy-induced cell death in MDA-MB-231 cells by activation of SIRT3. (**a**) Western blot analysis of MDA-MB-231 cells treated with **117c**, showing expression levels of p62 and LC3. (**b**) MTT assay evaluating cellular capacity with **117c** in the presence or absence of autophagy inhibitors. (**c**) Cell migration study with **117c** in the presence or absence of autophagy inhibitors. (**d**) Western blot analysis of MDA-MB-231 cells treated with **117c**, measuring expression levels of SIRT3, E-cadherin, MMP-2, and LC3. NS means no significance, *** *p* < 0.001, **** *p* < 0.0001, ^##^ *p* < 0.01, and ^###^ *p* < 0.001. Reprinted (adapted) with permission from ref. [53]. Copyright American Chemical Society, 2023.

On the other hand, the regulation of autophagy by SIRT3 could have a suppressive effect on tumor growth and elimination [53]. As depicted in Figure 41a, compound **117c** inhibits autophagy, as evidenced by the increase in LC3-II expression and the decrease in p56 expression following treatment. Moreover, the inhibition of autophagy resulted in a significant increase in cell viability and a notable attenuation of tumor migration (Figure 41b,c). This indicates that compound **117c** exerts its antiproliferative effect on MDA-MB-231 cells by inducing autophagy. To delve into the role of SIRT3 in this process, the researchers employed SIRT3-specific siRNA to attenuate SIRT3 expression. Intriguingly, the attenuation of SIRT3 resulted in a significant decrease in the induction of autophagy, which is evident from the reduced levels of LC3-II and p62, along with the down-regulation of E-cadherin (Figure 41d). These findings suggest that compound **117c** regulates autophagy and tumor migration by activating SIRT3.

**Figure 42:**
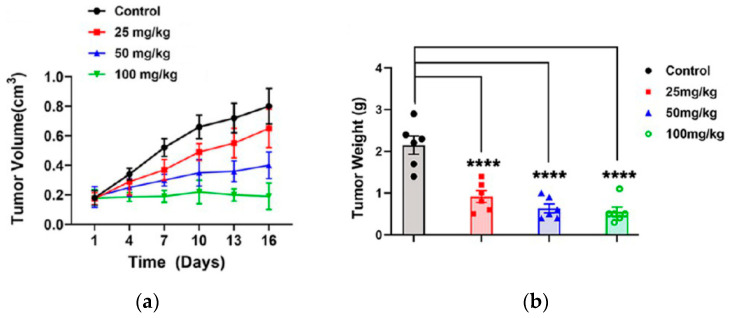
In vivo study conducted in a xenograft model with TNBC mice treated with compound **117c**. (**a**) Tumor volume and (**b**) tumor weight with compound **117c** treatment (*n* = 10 per group). Tumor volume data were collected every 3 days and presented as mean ± SD. Tumor weight data were collected on the final day and presented as mean ± SD. **** *p* < 0.001 compared with control group. Reprinted (adapted) with permission from ref. [53]. Copyright American Chemical Society, 2023.

Finally, in vivo studies were carried out to evaluate the antitumor activity of compound **117c** in TNBC mouse xenograft models at three different concentrations (25, 50, and 100 mg/kg) [53]. After 16 days of treatment, compound **117c** exhibited significant antiproliferative activity in a dose-dependent manner. Additionally, the administration of **117c** resulted in a notable decrease in tumor growth, tumor volume, and tumor weight compared to the control group (Figure 42a,b). However, when evaluating the toxicity of **117c**, certain degrees of toxicity were observed in vivo at high concentrations, leading to pulmonary septum widening and other cellular abnormalities.

**Figure 43:**
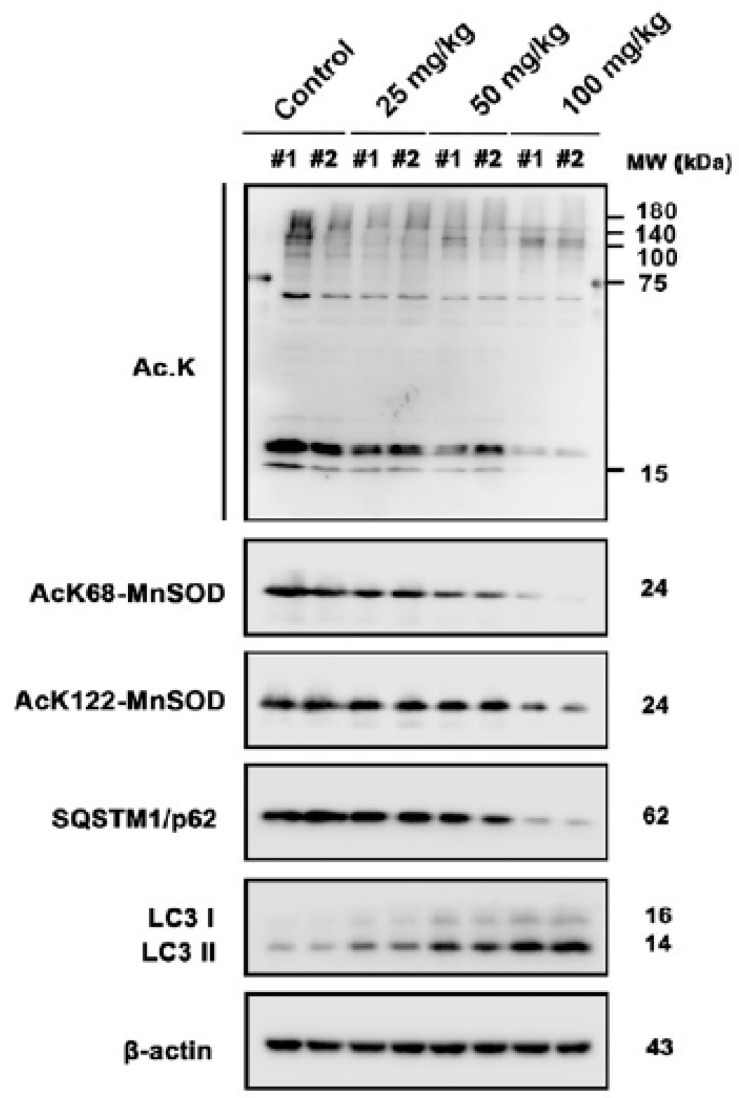
Western blot analysis of the expression levels of Ac. K, AcK68-MnSOD2, AcK122-MnSOD2, SQSTM1/p62, and LC3 in the MDA-MB-231 TNBC mouse xenograft model. *β*-Actin was used as the positive control. Reprinted (adapted) with permission from ref. [53]. Copyright American Chemical Society, 2023.

In addition to the previous findings, complementary in vivo studies were conducted to assess the ability of compound **117c** to activate SIRT3 and induce autophagy in a mouse xenograft model using MDA-MB-231 TNBC cells [53]. The research team measured acetyl-lysine (Ac. K) acetylation levels, specifically AcK68-MnSOD2 and AcK122-MnSOD2, and examined the expression levels of SQSTM1/p62 and LC3 (Figure 43). The results provided compelling evidence that the acetylation levels of K68-MnSOD2 and K122-MnSOD2 precisely matched the deacetylation sites of SIRT3. Moreover, a noticeable decrease in the expression of p62 was observed, while LC3-II levels were significantly up-regulated compared to the positive control (*β*-actin). These findings convincingly demonstrate the activation of autophagy by compound **117c**. In conclusion, these promising in vivo results underscore the potential of compound **117c**, in combination with SIRT3, as a promising alternative for the treatment of TNBC-type cancer.

In their pursuit of employing eco-friendly synthetic strategies and achieving favorable yields, Irfan et al. conducted the synthesis of benzo[*b*]furan-based oxadiazole/triazole derivatives **120a**–**g** and **121a**–**h** using ultrasound and microwave irradiation, respectively (Table 28) [54]. In method A, benzo[*b*]furan–oxadiazole derivatives **120a**–**g** were obtained in 60–90% yields through *S*-nucleophilic substitution of 5-(benzofuran-2-yl)-1,3,4-oxadiazole-2-thiol **118a** with bromoacetanilides **119a**–**g** utilizing pyridine in acetonitrile under ultrasound irradiation at 40 °C for 30 min. Similarly, method B was employed to synthesize benzo[*b*]furan–triazole derivatives **121a**–**h** in 68–96% yields through a *S*-nucleophilic substitution of 5-(benzofuran-2-yl)-4-phenyl-4*H*-1,2,4-triazole-3-thiol **118b** with bromoacetanilides **119a**–**h**, utilizing pyridine in DMF under microwave irradiation for 60–70 s. In summary, the microwave-assisted method B led to better yields and shorter reaction times for benzo[*b*]furan derivatives than the ultrasound-assisted method A.

The hemolytic, thrombolytic, and anticancer activities of previously synthesized benzo[*b*]furan-based oxadiazole/triazole derivatives were assessed (Table 29) [54]. Among these compounds, **121b** (X = NPh, R^1^ = *N*-morpholinyl) exhibited the lowest cytotoxicity (0.1%), while **121g** (X = NPh, R^1^ = 4-chloro-*N*-anilinyl) and **120b** (X = O, R^1^ = *N*-morpholinyl) showed the highest toxicity (23.4% and 22.12%, respectively) compared to ABTS (95.9%). In the thrombolysis assay, the majority of compounds demonstrated moderate activity when compared to the positive control (ABTS). Notably, compound **121f** (X = NPh, R^1^ = 2,4-dimethyl-*N*-anilinyl) exhibited the highest thrombolytic potential with a value of 61.4% compared to the positive control ABTS (86%).

The IC_50_ value and cell viability percentage against the A549 lung cancer cell line were determined using an MTT assay, with crizotinib and cisplatin as the standard drugs (Table 29). In summary, compound **120d** (X = O, R^1^ = 2-methoxy-*N*-anilinyl) exhibited the highest potency with a cell viability of 27.49% and an IC_50_ value of 6.3 µM, demonstrating greater activity than crizotinib (28.22% and 8.54 µM, respectively) and lower cytotoxicity than cisplatin (15.34% and 3.88 µM, respectively). In addition, compound **121h** (X = NPh, R^1^ = 2,4-dichloro-*N*-anilinyl) showed slightly lower activity with a cell viability of 29.29% and an IC_50_ value of 10.9 µM. Other oxadiazole/triazole derivatives, such as **120b**–**c**, **120e**–**g**, **121a**, **121c**–**e**, and **121g**, exhibited moderate anticancer activity, with cell viability ranging from 34.47% to 49.8%. Although compound **121f** (X = NPh, R^1^ = 2,4-dimethyl-*N*-anilinyl) had the highest cell viability (99.1%), it did not demonstrate any activity against the A549 cell line.

**Figure 44:**
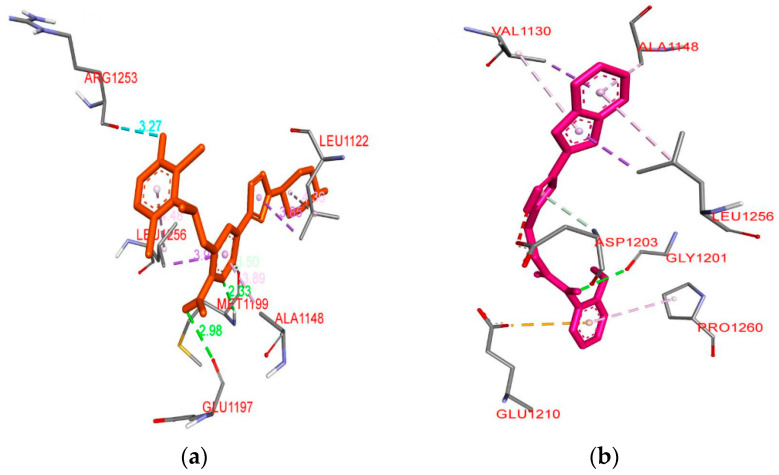
(**a**) Molecular docking of crizotinib in the ALK protein binding pocket (PDB ID code: 2XP2), and (**b**) 3D model of compound **120d** with its interacting amino acid residues. This is an open-access article distributed under the terms of the Creative Commons CC BY license [54].

A molecular docking simulation was performed to investigate the interactions of compound **120d** with anaplastic lymphoma kinase (ALK) in conjunction with crizotinib (PDB ID code: 2XP2) [54]. In summary, crizotinib displayed direct contact with ALK residues in the active site, while compound **120d** exhibited even more effective binding to these ALK residues (Figure 44a,b). Notably, the phenyl and heterocyclic rings of **120d** engaged in π-sigma interactions with Leu1256 and Val1130, while the NH group formed hydrogen bonds with Gly1201 and π-anion bonds with Glu1210.

**Scheme 20:**
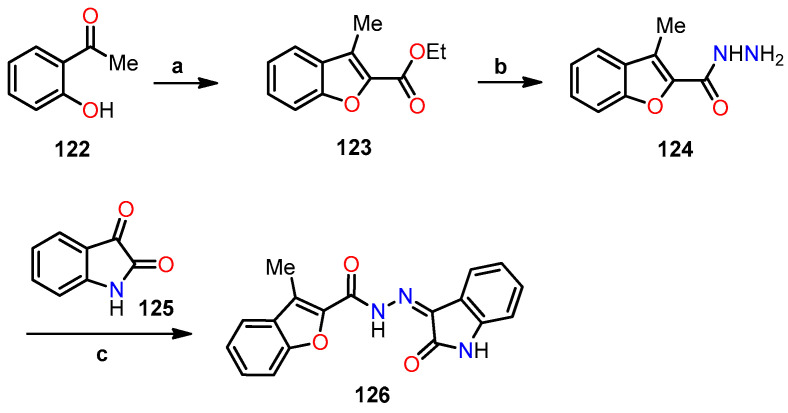
Synthesis of benzofuran–isatin hybrid **126**, and reagents and conditions: (**a**) ethyl 2-bromoacetate, CH_3_CN, K_2_CO_3_, reflux, 8 h; (**b**) hydrazine hydrate, MeOH, reflux, 4 h; (**c**) ethanol, AcOH, reflux, 5 h, 80%.

Isatin, also known as 1*H*-indole-2,3-dione, is an important *N*-heterocycle in medicinal chemistry and drug discovery [55]. In their study, Mohammed et al. synthesized an isatin–benzofuran hybrid **126** and investigated its antiproliferative activity against HT29 and SW620 cancer cell lines, along with its impact on tumor and metastatic development involved in primary cellular processes [56]. Scheme 20 shows the three-step synthesis of the isatin–benzofuran hybrid **126**. Firstly, 2-hydroxyacetophenone **122** underwent cyclization with ethyl bromoacetate utilizing K_2_CO_3_ as a base in refluxing acetonitrile for 8 h to afford ethyl 3-methylbenzofuran-2-carboxylate **123**, which then reacted with hydrazine hydrate in refluxing methanol for 4 h to give 3-methylbenzofuran-2-carbohydrazide **124**. Finally, a condensation reaction between **124** and isatin **125** catalyzed by acetic acid in refluxing ethanol for 5 h afforded isatin–benzofuran hybrid **126** with an 80% yield. 

**Figure 45:**
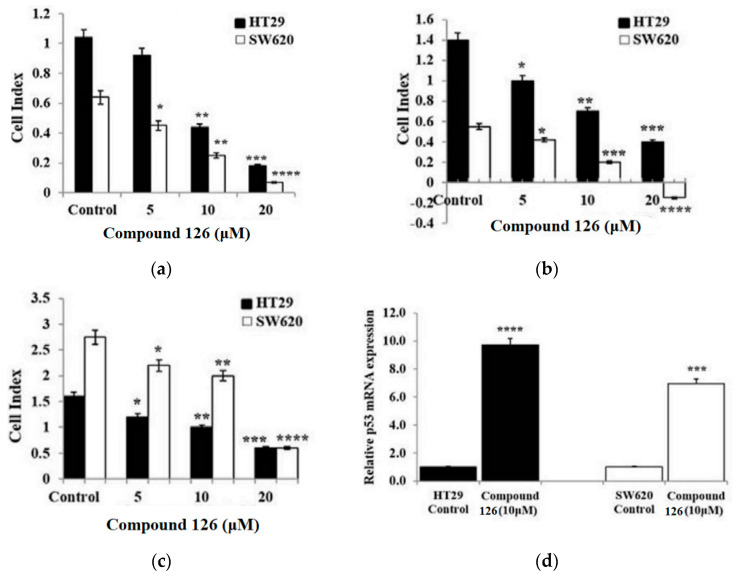
Inhibition of benzofuran–isatin hybrid **126** on (**a**) cell proliferation, (**b**) migration, (**c**) invasion in the HT29 and SW620 cancer cell lines, and (**d**) its influence on gene expression in the p53 protein. Data are shown as mean ± SD (*n* = 3). The data were considered significant when reporting * *p* < 0.05, ** *p* < 0.01, *** *p* < 0.001, and **** *p* < 0.0001 vs. control. This is an open-access article distributed under the terms of the Creative Commons CC BY license [56].

Next, comprehensive biological tests and analyses were carried out, specifically targeting cell viability, real-time migration, invasion studies, cell cycle assays related to apoptosis, and cytotoxicity evaluations [56]. In the initial phase, a cell viability, migration, and invasion assays were carried out using an xCELLigence Automated Dual Layer Real-time Cell Analyzer (RTCA-DP) with various concentrations of isatin–benzofuran hybrid **126**. The results revealed a noteworthy dose-dependent reduction in cell proliferation, migration, and invasion in both cancer cell lines compared to untreated cells (Figure 45a–c). Moreover, notable variations were observed in the inhibitory effects of hybrid **126** on the proliferation and migration of the SW620 line compared to the HT29 line, while in invasion, the HT29 line exhibited a more pronounced inhibitory effect. In addition, the impact of hybrid **126** on tumor suppression, based on the p53 protein, was evaluated. The results indicated a significant increase in p53 expression levels, 2.46-fold in HT29 cells, and 4.81-fold in SW260 cells at a concentration of 10 μM, demonstrating the potent inhibitory effect on tumor cell proliferation when utilizing hybrid **126** (Figure 45d).

**Figure 46:**
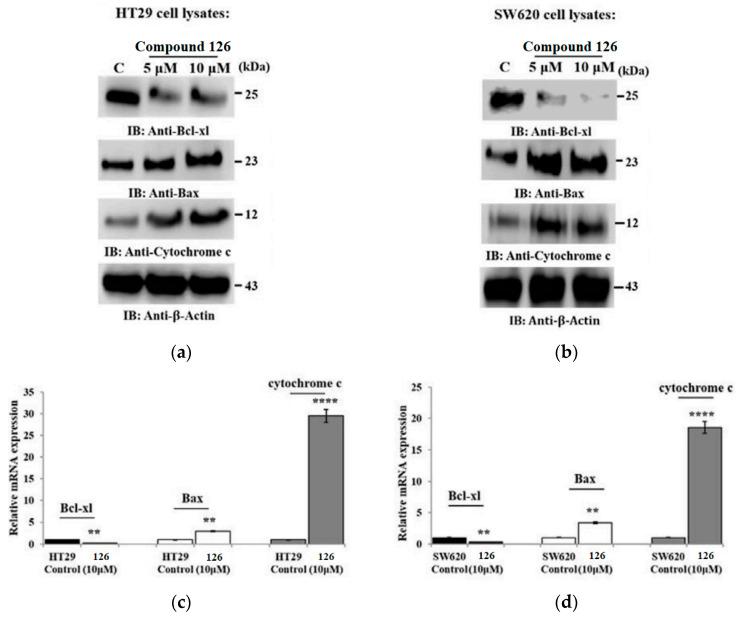
Flow cytometry analysis of hybrid **126** against cell lines (**a**) HT29 and (**b**) SW620 at Bcl-x, Bax, and cytochrome C expression levels with *β*-actin as the control. Additionally, bar graphs show the gene expression of these mitochondrial proteins for (**c**) HT29 and (**d**) SW620. Data are shown as mean ± SD (*n* = 3). ** *p* < 0.01 and **** *p* < 0.0001 vs. control. This is an open-access article distributed under the terms of the Creative Commons CC BY license [56].

Apoptotic studies were performed to evaluate the pro-apoptotic effects of hybrid **126**, involving its role in suppressing the expression levels of mitochondrial proteins Bcl-x, Bax, and cytochrome C utilizing flow cytometry [56]. The results showed a significant suppression of the Bcl-x protein expression by 50% in the HT29 cells, and by 75% and 90% in the SW620 cells at concentrations of 5 and 10 μM, respectively, in comparison to untreated cells (Figure 46a,b). On the other hand, a significant increase in the expression of Bax and cytochrome C was observed in both HT29 and SW620 cell lines, showing an approximately two-fold increase compared to the basal expression of untreated cells (Figure 46a,b). Moreover, the down-regulation of Bcl-x and the up-regulation of Bax and cytochrome C in both cancer cell lines exposed to hybrid **126** were further confirmed in gene expression levels, when compared to untreated cells. These findings support the apoptotic effects of hybrid **126** on cell cycle disruption in both HT29 and SW620 cell lines (Figure 46c,d).

**Figure 47:**
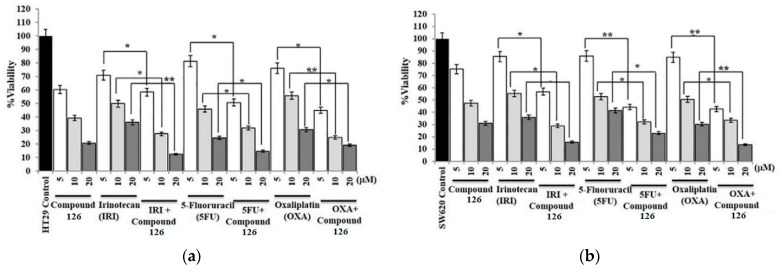
Effects of hybrid **126** on the cellular capacity of (**a**) HT29 and (**b**) SW620 cell lines in combination with three chemotherapeutic drugs: irinotecan (IRI), 5-fluorouracil (5-FU), and oxaliplatin (OXA) at concentrations of 5, 10, and 20 μM. Data are expressed as mean ± SD (*n* = 3). * *p* < 0.001 and ** *p* < 0.0001 vs. control. This is an open-access article distributed under the terms of the Creative Commons CC BY license [56].

In this particular study, they assessed the cytotoxic effect of hybrid **126** both alone and in combination with three anticancer drugs: irinotecan (IRI), 5-fluorouracil (5-FU), and oxaliplatin (OXA) in the HT29 and SW620 cancer cell lines at different concentrations (Figure 47a,b) [56]. Notably, when combining hybrid **126** with IRI in the HT29 cell line (Figure 47a), there was a significant inhibition of cell proliferation at 10 μM (−75% vs. −50%) and 20 μM (−90% vs. −65%) compared to the single treatment with IRI alone. When combining 5-FU with hybrid **126**, cell proliferation was significantly inhibited at 5 μM (−50% vs. −20%) and 10 μM (−67% vs. −55%) compared to the single drug treatment. Similarly, OXA inhibited cell proliferation at 5 μM (−55% vs. −25%) and 10 μM (−75% vs. −45%) when used in conjunction with hybrid **126**. On the other hand, in the SW620 cell line (Figure 47b), the combined treatment of IRI with **126** resulted in a substantial inhibition of cell proliferation at 5 μM (−45% vs. −15%) and at 10 μM (−75% vs. −45%). Similarly, 5-FU combined with **126** showed comparable inhibition of cell proliferation to IRI at 5 μM (−55% vs. −15%) and at 10 μM (−70% vs. −50%). Moreover, when utilizing the treatment in conjunction with OXA, a more pronounced inhibition of cell proliferation was observed at 5 μM (−55% vs. −15%), 10 μM (−65% vs. −50%), and 20 μM (−90% vs. −75%) compared to the single drug treatment. These findings demonstrate a significant enhancement in the effectiveness of the anticancer drugs employed in this study when combined with hybrid **126**.

### 2.2. Antibacterial Activity

Antibiotic resistance presents a worldwide concern, steadily escalating in severity, and posing substantial challenges to healthcare systems globally. Novel approaches are urgently needed to address this critical problem. Recently, benzo[*b*]furans and their derivatives have shown remarkable inhibitory potential against various Gram-positive bacteria, including *Staphylococcus aureus* (*S. aureus*), *Bacillus subtilis* (*B. subtilis*), and *Enterococcus* spp. (*E.* spp.), as well as Gram-negative bacteria, such as *Pseudomonas syringae* (*P. syringae*), *Klebsiella pneumoniae* (*K. pneumoniae*), *Salmonella typhi* (*S. typhi*), *Pseudomonas aeruginosa* (*P. aeruginosa*), and *Escherichia coli* (*E. coli*) [57]. In the province of Lampang, Thailand, a noteworthy medicinal discovery was made from the root extracts of *Stemona aphylla*, resulting in the isolation of alkaloids **127a**–**i** (Table 30) [58]. The extraction process involved drying 15.14 kg of ground root material, which was then extracted with 95% ethanol for 16 days at room temperature. After evaporating the extract, a portion of the residue was partitioned between 50% aqueous methanol and dichloromethane to give 8.86 g of the dichloromethane extract. The alkaloids were further isolated using column chromatography or preparative thin-layer chromatography through successive separations. The identified alkaloids and their corresponding masses are listed in Table 30.

Antimicrobial studies were conducted on alkaloids **127a**, **127c**–**d**, **127g**, and **127i**, evaluating their MIC values against two Gram-negative bacteria (*Escherichia coli* and *Klebsiella pneumoniae*) and three Gram-positive bacteria (*Staphylococcus aureus*, *methicillin-resistant Staphylococcus aureus* (*MRSA*), and *Streptococcus pyogenes*), with gentamicin used as the standard drug (Table 31) [58]. The results indicated minimal activity against Gram-negative bacteria for all tested compounds. However, alkaloids **127a** (R^1^ = H, R^2^ = H, R^3^ = Me, R^4^ = H, R^5^ = Me, R^6^ = H), **127c** (R^1^ = H, R^2^ = H, R^3^ = Me, R^4^ = Me, R^5^ = Me, R^6^ = H), and **127i** (R^1^ = H, R^2^ = OMe, R^3^ = Me, R^4^ = Me, R^5^ = Me, R^6^ = H) showed significant activity against *MRSA*, exhibiting MIC values of 15.6 µg/mL, outperforming the control antibiotic (MIC = 45.0 µg/mL). Compounds **127d**, **127g**, and **127i** showed moderate activity against *S. aureus*, with MIC values of 31.3 µg/mL, in comparison to gentamicin (MIC = 22.5 µg/mL).

Next, Ashok et al. synthesized a series of *E*-(1)-(6-benzoyl-3,5-dimethylfuro [3′,2′: 4,5]benzo[*b*]furan-2-yl)-3-(aryl)-2-propen-1-ones **130a**–**g** using both conventional and microwave heating protocols (Table 32) [59]. Firstly, they obtained 2-acetyl-3,5-dimethyl-6-benzoylbenzodifuran **129** by cyclizing 5-acetyl-2-benzoyl-6-hydroxy-3-methylbenzo[*b*]furan **128** with 2-chloroacetone employing K_2_CO_3_ as a base in refluxing acetone for 8 h (Method A), and microwave heating at 120 °C for 4 min under solvent-free conditions (Method B). Subsequently, *bis*-chalcones **130a**–**g** were synthesized through a Claisen–Schmidt condensation reaction of **129** with (hetero)aromatic aldehydes using NaOH as a base in refluxing ethanol for 6–8 h (Method A, 53–68%) and microwave-assisted aldol condensation at 90 °C for 4–5 min under solvent-free conditions (Method B, 87–94%). In summary, method B led to *bis*-chalcones in higher yields and shorter reaction times compared to method A, which utilized conventional heating.

The antimicrobial activities of *bis*-chalcones **130a**–**g** were evaluated using the plate count method with nutrient agar as the culture medium. The bacterial strains tested included two Gram-negative bacteria, *Escherichia coli* and *Pseudomonas aeruginosa*, and two Gram-positive bacteria, *Bacillus subtilis* and *Staphylococcus aureus*, using chloramphenicol, carbenicillin, streptomycin, and tetracycline as the standard drugs [59]. The inhibition zones (in mm) were measured after 24 h of incubation at 37 °C (Table 33). In summary, *bis*-chalcones **130a**–**g** showed inhibition zones ranging from 6 to 11 mm and 7 to 17 mm against Gram-negative and Gram-positive bacterial strains, respectively. In particular, compounds **130a** (Ar = Ph), **130b** (Ar = 2-ClPh), **130f** (Ar = 1,3-diphenyl-1*H*-pyrazol-4-yl), and **130g** (Ar = 1-phenyl-3-(4-bromophenyl)-1*H*-pyrazol-4-yl) showed good activity against all bacterial strains, with inhibition zones in a range of 9 to 17 mm. However, none of these *bis*-chalcones demonstrated higher activity than the control drugs for each bacterial strain (13–22 mm).

**Scheme 21:**
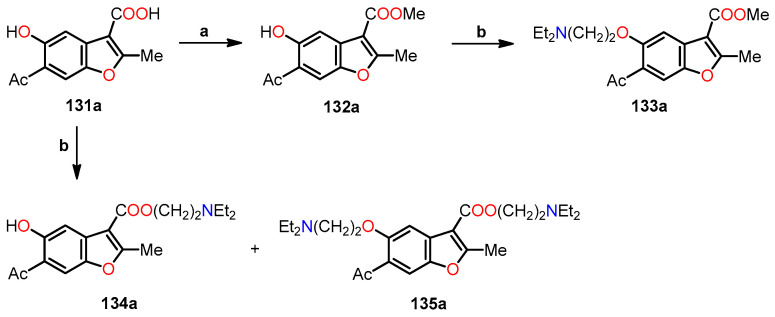
Synthesis of *O*-alkylamino benzo[*b*]furancarboxylates **132**–**135**, and reagents and conditions: (**a**) MeOH, H_2_SO_4_; (**b**) Cl(CH_2_)_2_NEt_2_.HCl, K_2_CO_3_, Aliquat 336 (*N*-methyl-*N,N*-dioctyloctan-1-ammonium chloride), acetone, MWI (4–8 cycles: heating 6 min and cooling 2 min).

In a separate study, Ostrowska et al. utilized microwave irradiation to synthesize a collection of *O*-alkylamino benzo[*b*]furancarboxylates with good yields [60,61]. Firstly, the esterification reaction of 6-acetyl-5-hydroxy-2-methyl-3-benzo[*b*]furancarboxylic acid **131a** with methanol catalyzed by sulfuric acid afforded benzo[*b*]furancarboxylate **132a** (Scheme 21). Subsequently, microwave-assisted *O*-alkylation reaction of **132a** with 2-chloroethyl-*N,N*-diethylamine in the presence of K_2_CO_3_ and Aliquat 336 in acetone gave *O*-alkylated benzofuran-3-carboxylate **133a**. In an alternative approach, compounds **134a** and **135a** were synthesized under analogous conditions to the previous *O*-alkylation protocol, using carboxylic acid **131a** instead of ester **132a**. Notably, compounds **133b**–**g** and **134b**–**g** were obtained using the corresponding benzo[*b*]furancarboxylic acids **131b**–**g** as the initial reactants (Table 34). Finally, the benzo[*b*]furancarboxylate derivatives **133a**–**d**, **133f**, **134a**–**d**, **134f**, and **135a** were transformed into their respective hydrochloride salts.

The antimicrobial activity of hydrochlorides of benzo[*b*]furancarboxylates **133a**–**d**, **133f**, **134a**–**d**, **134f**–**g**, and **135a** was screened against six Gram-positive bacterial strains, including *Micrococcus luteus*, *Bacillus cereus*, *Bacillus subtilis*, *Staphylococcus epidermidis*, *Staphylococcus aureus*, and *Enterococcus hirae*, along with two Gram-negative bacterial strains, such as *Escherichia coli* and *Pseudomonas aeruginosa* (Table 35). The results unveiled that the *O*-alkyl-benzo[*b*]furancarboxylate **133f.HCl** (R^1^ = COOMe, R^2^ = H, R^3^ = Br, R^4^ = OMe, R^5^ = O(CH_2_)_2_NEt_2_) showed the highest potency, with MIC values ranging from 0.003 to 0.012 µmol/cm^3^ against all Gram-positive bacterial strains. Conversely, the hydroxy-benzo[*b*]furancarboxylate **134g.HCl** (R^1^ = COO(CH_2_)_2_NEt_2_, R^2^ = H, R^3^ = H, R^4^ = H, R^5^ = OMe) showed the lowest potency, with an MIC value of 15.28 µmol/cm^3^ in all Gram-positive bacterial strains. Additionally, it becomes evident that within the Gram-positive strains, compounds **135a.HCl** and **133f.HCl** exhibited the most remarkable activity against *E. coli* and *P. aeruginosa* with MIC values of 0.59 and 3.12 µmol/cm^3^, respectively. Notably, the observed structure–activity relationships are as follows: (i) compound **133c.HCl** exhibited higher activity than **133b.HCl** due to the introduction of a methoxy group at the C–5 position, (ii) compound **133d.HCl** displayed higher activity than **133b.HCl** due to the presence of the 7-(4-methoxycinnamoyl) group, and (iii) 2-(*N,N*-diethylamino)ethyl esters **134a.HCl**, **134c.HCl**, and **134d.HCl** exhibited superior activity against Gram-positive strains compared to compounds **133a.HCl**, **133c.HCl**, and **133d.HCl**. These variations underscore the influential role of substituents in the benzo[*b*]furan moiety, as evidenced by their distinct activities against diverse bacterial strains.

**Scheme 22:**
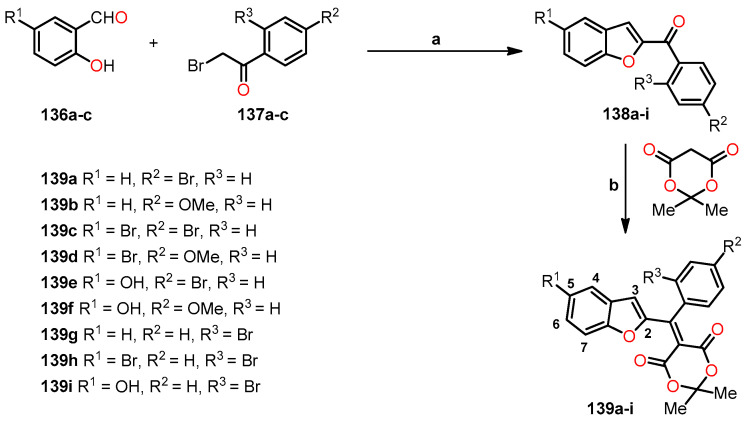
Synthesis of benzo[*b*]furan derivatives **139a**–**i**, and reagents and conditions: (**a**) K_2_CO_3_, CH_3_CN, reflux; (**b**) AcOH, reflux, 8–10 h, 110–115 °C, 75–91%.

Concurrently, Kenchappa et al. synthesized a series of (5-substituted-1-benzofuran-2-yl)(2,4-phenyl-substituted)methanones **139a**–**i** by incorporating a pharmacophore group at the 2 position of the benzo[*b*]furan ring, a response to the consistent trend of enhanced antimicrobial activity observed across various studies [62]. Initially, the synthesis commenced with the cyclization reaction of salicylaldehyde derivatives **136a**–**c** with *α*-bromoacetophenones **137a**–**c** utilizing potassium carbonate as a base in refluxing acetonitrile to afford benzo[*b*]furan derivatives **138a**–**i** (Scheme 22). Subsequently, the Knoevenage condensation of compounds **138a**–**i** with Meldrum’s acid catalyzed by acetic acid at temperatures of 110–115 °C for a duration of 8–10 h resulted in the formation of (5-substituted-1-benzofuran-2-yl)(2,4-phenyl)methanones **139a**–**i** in 75–91% yields. It is important to emphasize that the presence of acetic acid facilitated the generation of a carbanion in Meldrum’s acid, thereby enhancing the nucleophilic addition and subsequent dehydration processes.

The antimicrobial activity of benzo[*b*]furan derivatives **139a**–**i** was screened against one Gram-positive bacterial strain, including *Bacillus subtilis*, as well as four Gram-negative bacterial strains, including *Pseudomonas syringae*, *Salmonella typhi*, *Klebsiella pneumoniae*, and *Escherichia coli*, using the agar well diffusion method [62]. Streptomycin was employed as the standard reference. The minimum inhibitory concentration (MIC) studies were performed using a serial broth-dilution method at different concentrations, including 1, 10, 25, 50, and 100 mol/L [62]. Based on the findings presented in Table 36, compound **139c** (R^1^ = Br, R^2^ = Br, R^3^ = H) showed the most potent activity against the Gram-positive strain with an inhibition zone of 13 mm, closely approximating the effectiveness of streptomycin (16 mm) at a concentration of 0.5 mg/mL. Interestingly, compound **139c** also emerged as the most effective against all Gram-negative strains with inhibition zones ranging from 10 to 14 mm, akin to the performance of streptomycin (13–17 mm), at a concentration of 0.5 mg/mL. As shown in Table 37, compounds **139c** (R^1^ = Br, R^2^ = Br, R^3^ = H) and **139a** (R^1^ = H, R^2^ = Br, R^3^ = H) demonstrated remarkable activity across all bacterial strains, displaying MIC values in ranges of 14.80–16.00 µg/mL and 15.50–16.50 µg/mL, aligning closely with the efficacy of streptomycin (MIC = 14.8–16.0 µg/mL). In contrast, compounds **134b** (R^1^ = H, R^2^ = OMe, R^3^ = H) and **139f** (R^1^ = OH, R^2^ = OMe, R^3^ = H) showed reduced activity, possibly attributed to the presence of electron-donating groups at the C–5 position of the benzo[*b*]furan ring. Furthermore, the introduction of a bromine group at C–4 of the benzo[*b*]furan ring did not increase the activity of compound **139i** (R^1^ = OH, R^2^ = H, R^3^ = Br) (Table 37).

**Scheme 23:**
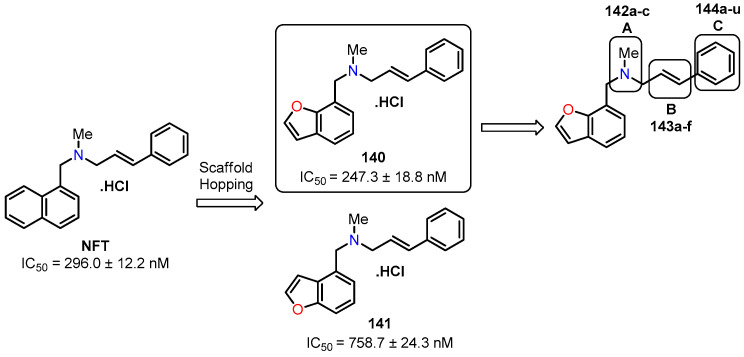
Scaffold hopping from the active compound NTF, featuring a central nucleotide modification (**140**–**141**) along with three structural modifications (**142**–**144**).

**Scheme 24:**
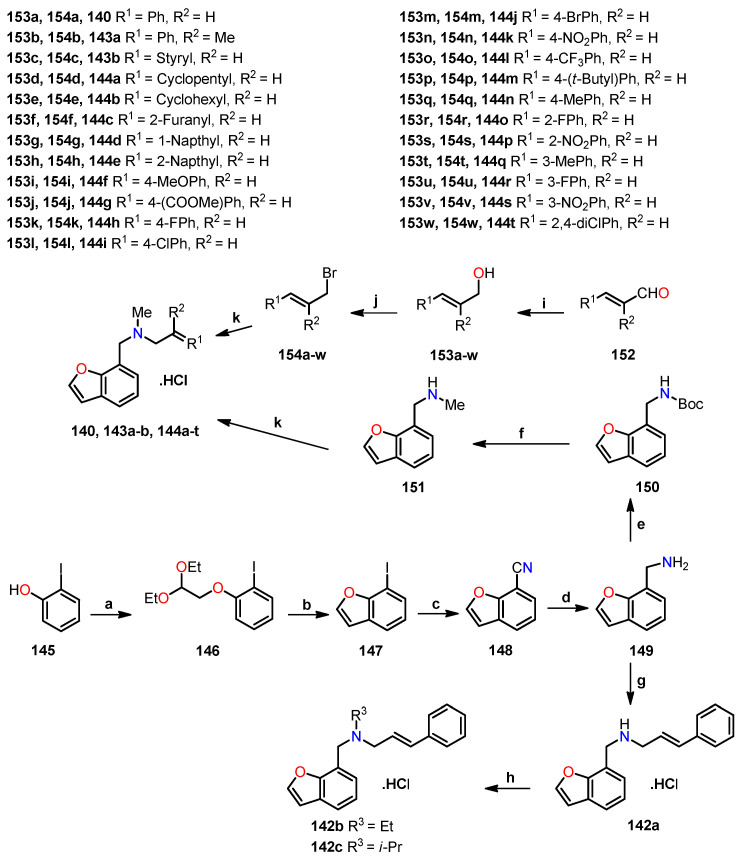
Synthesis of benzo[*b*]furan derivatives **140**, **142a**–**c**, **143a**–**b**, and **144a**–**t**, and reagents and conditions: (**a**) 1-bromo-2,2-diethoxyethane, NaH, DMF, 90 °C, overnight, 93%; (**b**) polyphosphoric acids, toluene, reflux, overnight, 55%; (**c**) CuCN, DMF, 130 °C, 4 h, 90%; (**d**) LiAlH_4_, THF, −78 °C to r.t, overnight, N_2_, 95%; (**e**) di-*tert*-butyl dicarbonate, NaOH, THF, 0 °C to r.t, 1 h, 85%; (**f**) LiAlH_4_, THF, 0 °C to r.t, overnight, N_2_, 95%; (**g**) (*i*) *trans*-cinnamaldehyde, molecular sieves, CH_2_Cl_2_, reflux, 17 h; (*ii*) NaBH_4_, methanol, 0 °C, 30 min, 95% (2 steps); (**h**) iodoethane or 2-iodopropane, NaH, DMF 0 °C to r.t, overnight, N_2_, 50%; (**i**) NaBH_4_, methanol, 0 °C, 30 min; (**j**) PBr_3_, Et_2_O, 0 °C to r.t, overnight, N_2_, 57–84% (2 steps); (**k**) (*i*) K_2_CO_3_, DMF, r.t, overnight, 43–85%, (*ii*) bubbled into hydrogen chloride gas.

Naftifine, a topical allylamine, exhibits effectiveness across an extensive spectrum of dermatophytic fungi, including *Trichophyton* and *Microsporum* spp., and has also shown significant efficacy against *Candida* and *Aspergillus* spp. [63]. In 2016, Wang et al. undertook the synthesis of naphthalene hydrochloride (NFT) derivatives, previously recognized as potent inhibitors of the diapophytoene desaturase (CrtN) enzyme, which is a crucial molecular target against infections caused by pigmented *Staphylococcus aureus* [64]. The process of molecular design comprised several sequential stages. It commenced with an analysis of the naphthalene moiety of NFT, which served as a potential pharmacophore group. Subsequently, modifications were introduced in the *N*-methyl group, involving various steric groups (region A). Concurrently, the synthesis of specific analogs was undertaken to explore the impact of different linker types within the allyl portion on inhibitory activity (region B). Finally, a meticulous design approach led to the synthesis of 21 analogs, each featuring distinct substituents (region C), as illustrated in Scheme 23. In pursuit of this goal, several syntheses were undertaken to generate the varied analogs portrayed in Scheme 24.

Scheme 24 sowed synthetic procedures to synthesize a series of naftifine analogs—**140**, **142a**–**c**, **143a**–**b**, and **144a**–**t** [64]. The synthesis began with the nucleophilic substitution of 2-iodophenol **145** utilizing 1-bromo-2,2-diethoxyethane and NaH in DMF at 90 °C to give compound **146**, which was cyclized in the presence of polyphosphoric acids under refluxing toluene to yield 7-iodobenzofuran **147**. Further progression involved the substitution of the iodine atom in **147** with a cyano group in DMF at 130 °C for 4 h to furnish benzofuran-7-carbonitrile **148**. The subsequent reduction of the cyano group in **148** was performed with LiAlH_4_ under mild reaction conditions to obtain benzofuran-7-ylmethanamine **149**. Later, allylamine **142a** was synthesized with an overall yield of 95% through a reductive amination reaction of compound **149** with *trans*-cinnamaldehyde utilizing NaBH_4_ as the reducing agent. Subsequently, the *N*-alkylation of allylamine **142a** was conducted using iodoethane or 2-iodopropane in the presence of NaH as a base in DMF at ambient temperature to deliver compounds **142b**–**c** with a purity ≥ 95%. In another synthetic strategy, amine **149** was initially protected with di-*tert*-butyl dicarbonate, followed by reduction using LiAlH_4_ to afford 1-(benzofuran-7-yl)-*N*-methylmethanamine **151** (Scheme 24). Simultaneously, the *α*,*β*-unsaturated aldehyde **152** were reduced using NaBH_4_ to afford allylic alcohol **153**, which was subjected to an Appel reaction utilizing PBr_3_ in Et_2_O at ambient temperature to afford allylic bromide **154**. Finally, aliphatic nucleophilic substitution between compounds **151** and **154** gave a series of naftifine analogs, which were converted into their corresponding hydrochloride salts **140**, **143a**–**b**, and **144a**–**t**.

**Scheme 25:**
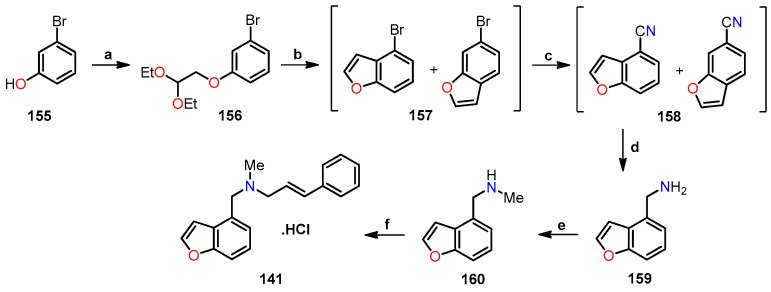
Synthesis of benzo[*b*]furan derivative **141**, and reagents and reaction conditions: (**a**) 1-bromo-2,2-diethoxyethane, NaH, DMF, 90 °C, overnight, 95%; (**b**) polyphosphoric acids, toluene, reflux, overnight; (**c**) CuCN, DMF, 130 °C, 4 h, 30% (2 steps); (**d**) LiAlH_4_, THF, −78 °C to r.t, overnight, under N_2_, 94%; (**e**) (*i*) di-*tert*-butyl dicarbonate, NaOH, THF, 0 °C, r.t, 1 h; (*ii*) LiAlH_4_, THF, 0 °C to r.t, overnight, N_2_, 70% (2 steps); (**f**) (*i*) cinnamyl bromide, K_2_CO_3_, DMF, r.t, overnight, 44%, (*ii*) bubbled into hydrogen chloride gas.

In a similar manner, the synthesis of naftifine analog **141** involved an *O*-alkylation reaction of 3-bromophenol **155** with 1-bromo-2,2-diethoxyethane to give compound **156**, which was then cyclized using polyphosphoric acids, leading to the formation of two isomeric products **157**, namely, 4-bromobenzo[*b*]furan and 6-bromobenzo[*b*]furan (Scheme 25). Subsequently, the bromine atom within isomers **157** was substituted with a cyano group to afford isomers **158**. Finally, through a series of consecutive reactions involving reduction and Boc_2_O protection, reduction, and nucleophilic substitution, the naftifine analog **141** was successfully synthesized.

After obtaining the desired analogs, their inhibitory potential against *S. aureus* Newman was systematically evaluated using naftifine (NFT) as the reference drug [64]. The results indicated that compound **140** (R^3^ = Me, R^5^ = 7-benzofuranyl) showed the highest potency with an IC_50_ value of 247.3 nM, in comparison to NFT as the standard drug (IC_50_ = 296.0 nM). In contrast, the isomeric compound **141** demonstrated low activity with an IC_50_ value of 758.7 nM. In the case of analogs featuring *N*-methyl substitutions **142a**–**c**, the incorporation of an ethyl or *iso*-propyl group markedly diminished activity (IC_50_ > 1000 nm), as shown in Table 38. While the incorporation of cycloalkyl substituents **144a**–**b** and the 2-furanyl group **144c** did not improve activity (IC_50_ > 1000 nM), the incorporation of 1- and 2-naphthalenyl groups **144d** and **144e** led to better activity (IC_50_ = 887.7 and 17.1 nM, respectively). It is worth noting that the presence of electron-donating and electron-withdrawing groups attached to the aromatic ring significantly influenced the activity profile. Also, the position of the substituent on the aromatic ring affected the activity, such as compounds **144h** (R^1^ = 4-FPh, IC_50_ = 31.2 nM) vs. **144o** (R^1^ = 2-FPh, IC_50_ = 288.3 nM) vs. **144r** (R^1^ = 3-FPh, IC_50_ = 513.0 nM). The same behavior was observed for compounds **144k** (R^1^ = 4-NO_2_Ph, IC_50_ = 71.1 nM) vs. **144p** (R^1^ = 2-NO_2_Ph, IC_50_ > 1000 nM) vs. **144s** (R^1^ = 3-NO_2_Ph, IC_50_ > 1000 nM). A key highlight is the exceptional activity displayed by compound **144l** (R^1^ = 4-CF_3_Ph), showcasing an impressive IC_50_ value of 4.0 nM, which is 74 times lower than NFT (IC_50_ = 296.0 nM), as shown in Table 39.

Initial investigations unveiled the most prospective analogs with the potential to inhibit diapophytoene desaturase (CrtN) enzyme in the Staphyloxanthin (STX) biosynthesis pathway. STX is a notable golden carotenoid pigment synthesized by *S. aureus*, which opens up a novel avenue for treating *S. aureus* or *methicillin-resistant S. aureus* (MRSA) infections [64]. As shown in Table 40, a selection process identified five analogs (**144f**, **144i**, **144j**, **144l**, and **144t**) with the highest activity against *S. aureus* Newman, which were subjected to an evaluation of their capacity to inhibit the CrtN enzyme. The results revealed that five analogs displayed a remarkable 40-fold increase in inhibitory potency against CrtN compared to NFT. Interestingly, this potent inhibition of CrtN stands in contrast to their comparatively weaker impact on the enzymatic activity of pigmented *S. aureus* Newman. Furthermore, an assessment was conducted on the water solubility of the five analogs, revealing NFT to possess low solubility (6.2 mg/mL). This investigation facilitated the clarification of the interplay between solubility and chemical structure. Indeed, the replacement of the naphthalene ring with a benzo[*b*]furan ring generated an elevation in solubility, particularly evident in the cases of analogs **144f** and **144l**, showcasing solubilities 2–3 times greater than that of NFT, measuring 19.7 and 10.0 mg/mL, respectively. These findings served as the basis for advancing the assessment of the compound **144l**, both in vitro and in vivo.

**Figure 48:**
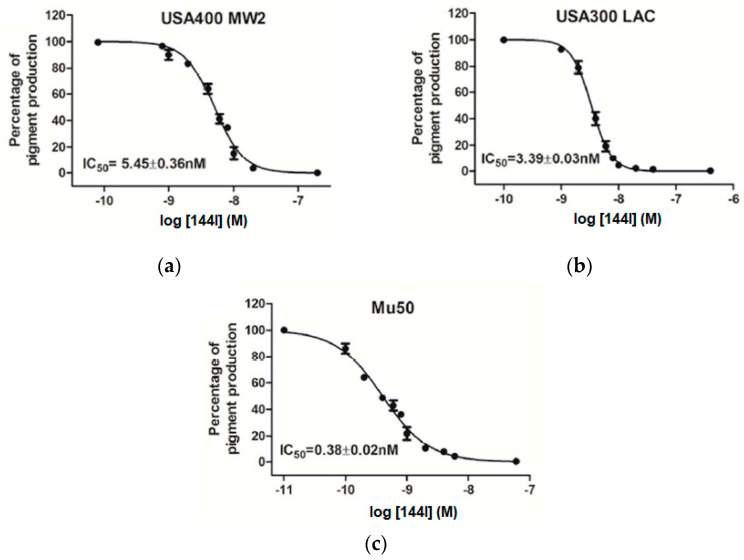
Dose–response curves in pigment formation across three MRSA strains: (**a**) USA400 MW2, (**b**) USA300 LAC, and (**c**) Mu50, with their corresponding IC_50_ values using the compound **144l**. This is an open-access article distributed under the terms of the Creative Commons CC BY license [64].

In their in vitro investigations, Wang et al. examined the impact of compound **144l** on three MRSA strains: USA400 MW2, USA300 LAC, and Mu50 [64]. The results revealed a reduction in color due to the inhibitory effects of **144l**, evidenced by IC_50_ values of 5.45, 3.39, and 0.38 nM. These findings mirrored the observations made with the *S. aureus* Newman strain (Figure 48).

**Figure 49:**
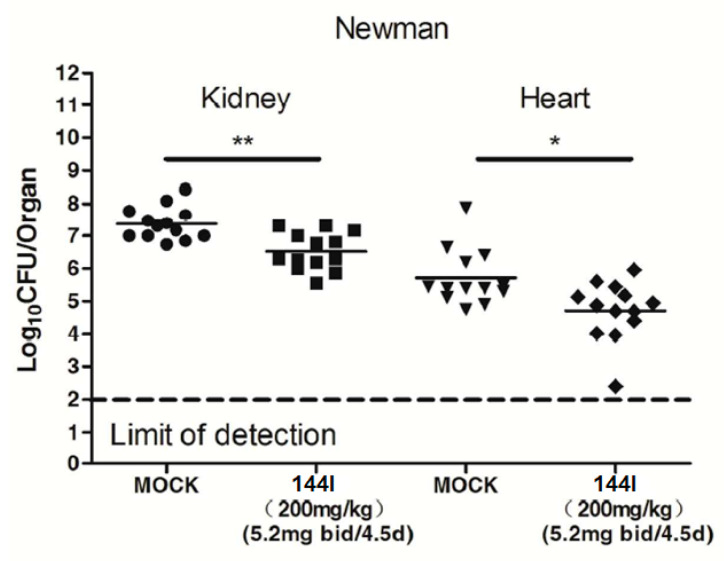
Effect of the compound **144l** on the virulence of *S. aureus* Newman in mouse kidneys and hearts (*n* = 13), quantified in terms of colony-forming units (Log_10_CFUs/organ). Statistical significance determined by the Mann−Whitney test (two-tailed): * *p* < 0.05 and ** *p* < 0.01. This is an open-access article distributed under the terms of the Creative Commons CC BY license [64].

In vivo studies allowed the evaluation of virulence reduction in three of four colonies. Mice were infected with mock or treated with the compound **144l** with *S. aureus* Newman, USA400 MW2, and Mu50 strains by retro-orbital injection [64]. Bacterial survival within host organs was then measured. Notably, in the *S aureus* Newman strain, the group treated with compound **144l** displayed a reduction in bacterial survival. Kidneys and hearts showed decreases of 0.85 and 1.01 log_10_CFU/organ, respectively (Figure 49).

**Figure 50:**
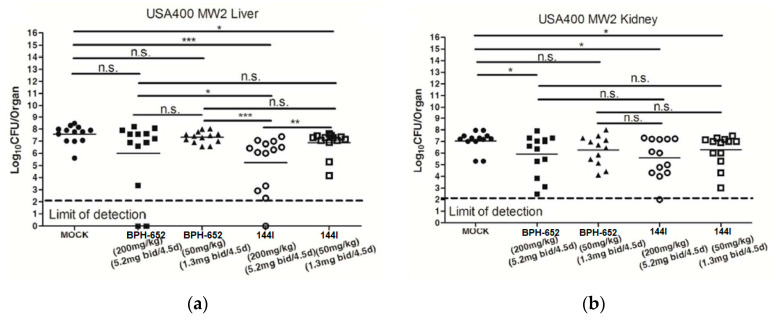
Effect of BPH-652 and compound **144l** on the virulence of USA400 MW2 in the livers (**a**) and kidneys (**b**) of mice (*n* = 13) as colony-forming units (Log_10_CFUs/organ). Statistical significance determined by the Mann−Whitney test (two-tailed): * *p* < 0.05, ** *p* < 0.01, *** *p* < 0.001, and n.s. indicates no significant difference. This is an open-access article distributed under the terms of the Creative Commons CC BY license [64].

Regarding the MRSA strains, BPH-652 served as a reference CrtN inhibitor [64]. For the USA400 MW2 strain, the administration of a 200 mg/kg dose of compound **144l** to mice resulted in a remarkable 99.6% reduction in survival rates within hepatic organs (2.35 log_10_CFU). Impressively, this outcome surpassed that of the BPH-652-treated group (1.58 log_10_CFU). Furthermore, with the dosage scaled down to 50 mg/kg, the bacterial survival rate showed only a marginal increase in both scenarios while still maintaining superiority over BPH-652 by 0.71 log_10_CFU in the **144l**-treated group and by 0.25 log10CFU in the BPH-652-treated group (Figure 50a). Within the renal organs, the administration of the 200 mg/kg dose resulted in a notable 96.6% decrease in staphylococcal loads of the **144l**-treated group (1.47 log_10_CFU), surpassing the BPH-652-treated group outcome of 1.14 log_10_CFU. Upon reducing the dose to 50 mg/kg, slight increases in bacterial survival rates were noted compared to the high-dose treatment groups (Figure 50b).

**Figure 51:**
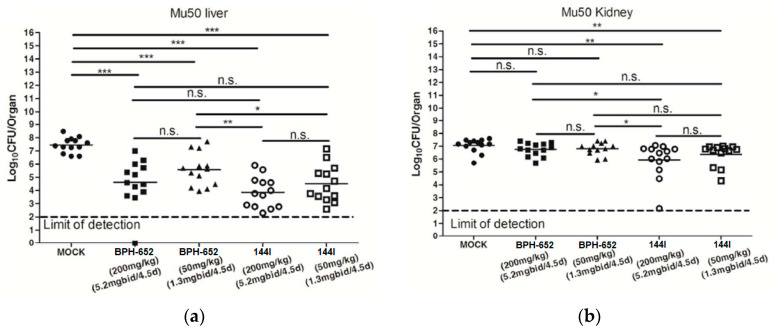
Effect of BPH-652 and compound **144l** on Mu50 virulence in the livers (**a**) and kidneys (**b**) of mice (*n* = 13) as colony-forming units (Log_10_CFUs/organ). Statistical significance determined by the Mann−Whitney test (two-tailed): * *p* < 0.05, ** *p* < 0.01, *** *p* < 0.001, and n.s. indicates no significant difference. This is an open-access article distributed under the terms of the Creative Commons CC BY license [64].

Employing the Mu50 strain, a parallel pattern emerged. Within liver organs, a dose of 200 mg/kg led to a reduction in survival rates by 3.58 log_10_CFU (**144l**-treated) and 2.84 log_10_CFU (BPH-652-treated). Similarly, at 50 mg/kg, the survival rate diminished by 2.94 log_10_CCFU (**144l**-treated) and 1.87 log_10_CFU (BPH-652-treated), as depicted in Figure 51a. However, outcomes in renal organs yielded inconclusive results. With a dosage of 200 mg/kg, the decrease amounted to 1.11 log_10_CFU (**144l**-treated) and merely 0.30 log_10_CFU (BPH-652-treated). At 50 mg/kg, survival was reduced to 0.68 log_10_CFU (**144l**-treated) and 0.25 log_10_CFU (BPH-652-treated), as shown in Figure 51b.

**Scheme 26:**
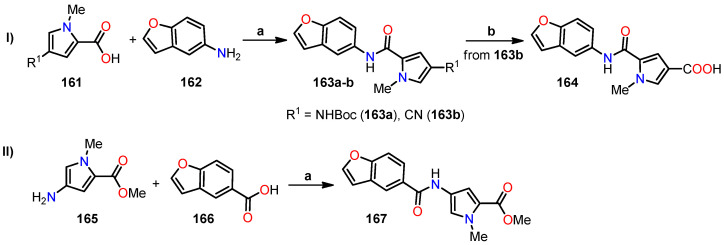
Synthesis of benzo[*b*]furan-based pyrroles, and reagents and reaction conditions: (**I**) (**a**) EDCI, DMAP, DMF, 67–79%; (**b**) dioxane, H_2_SO_4_, reflux, 9%; (**II**) (**a**) EDCI, DMAP, DMF, 20%.

**Scheme 27:**
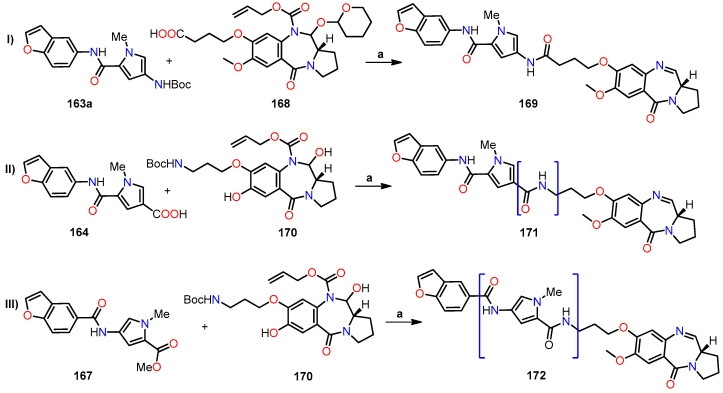
Synthesis of benzo[*b*]furan-based pyrrolobenzodiazepines, and reagents and reaction conditions: (**I**) and (**II**) (**a**) (*i*) TFA, DCM, (*ii*) EDCI, DMAP, DMF, (*iii*) pyrrolidine, Pd(PPh_3_)_4_, PPh_3_, DCM, 14–38%; (**III**) (**a**) (*i*) NaOH, H_2_O, dioxane, (*ii*) TFA, DCM, (*iii*) EDCI, DMAP, DMF, (*iv*) pyrrolidine, Pd(PPh_3_)_4_, PPh_3_, DCM, 22%. Bracketed structures denote compounds with an inverted orientation.

On the other hand, pyrrolobenzodiazepines (PBDs) have garnered considerable attention as promising antibacterial agents derived from natural sources. In line with this, Andriollo et al. undertook the synthesis of a series of PBDs incorporating C–8 linkers (Scheme 26), with the primary objective of evaluating their bioactivity and elucidating the structure–activity relationship (SAR) [65]. The synthetic methodology entails the synthesis of benzo[*b*]furan-based pyrroles **163a**–**b** and **167**, achieved through an amidation reaction between *N*-methylpyrrole derivatives and benzo[*b*]furans in DMF, utilizing the EDCI/DMAP coupling system. Subsequently, nitrile **163b** underwent hydrolysis under reflux conditions using dioxane and H_2_SO_4_ to afford carboxylic acid **164** in a modest 9% yield (Scheme 26). After acquiring these intermediate components, the synthesis of PBD derivatives **169**, **171**, and **172** with C–8 linkers was undertaken (Scheme 27). In this stage, the deprotection of BOC-protected amines **163a** and **170** was achieved by treating them with an acidic solution (TFA in DCM). Additionally, derivatives containing methyl esters, identified as **167**, underwent hydrolysis using an aqueous NaOH solution. Subsequent to this, an amide coupling reaction was facilitated utilizing the EDCI/DMAP coupling system, effectively linking the PBD core and side chains. Lastly, employing pyrrolidine and Pd(PPh_3_)_4_ in DCM, the conjugates underwent deprotection, leading to the generation of PDB derivatives **169**, **171**, and **172**, each exhibiting standard and reverse orientations of the amide bond (Scheme 27).

The previously obtained PBD derivatives **169**, **171**, and **172** underwent an assessment to determine their capacity to bind to DNA and impart stability using a Förster resonance energy transfer (FRET)-based DNA fusion assay, utilizing netropsin as the positive control [65]. To achieve this objective, two oligonucleotide sequences labeled with distinct fluorophores were utilized: sequence F1′-FAM-TAT-ATA-TAG-ATA-TTT-TTT-TAT-CTA-TAT-ATA-3′-TAMRA and sequence F2′-FAM-TAT-AGA-TAT-AGA-TAT-TAT-TTT-ATA-TCT-ATA-TCT-ATA-TCT-ATA-3′-TAMRA. Here, FAM corresponds to 6-carboxyfluorescein, and TAMRA represents 5-carboxytetramethylrhodamine. The results revealed that compound **169**, featuring a conventional orientation, adeptly conferred substantial stability to both DNA sequences, akin to the notable effect seen with netropsin. In contrast, compounds **171** and **172**, characterized by reversed orientations, displayed a clear inability to confer stability upon either DNA sequence, as evidenced by ΔT_m_ values below 1 °C. This compelling observation strongly suggests that the inversion of one or more amide bonds within these compounds markedly curtailed their DNA stabilizing efficacy, as firmly corroborated by the comprehensive data outlined in Table 41.

Subsequently, PBD derivatives **169**, **171**, and **172** were subjected to antimicrobial testing against diverse Gram-positive bacterial strains, including methicillin-sensitive *S. aureus* (MSSA) strain ATCC 9144, as well as two methicillin-resistant *S. aureus* (MRSA) strains, namely, EMRSA-15 (strain HO 5096 0412) and EMRSA-16 (strain MRSA 252) [65]. Additionally, the study incorporated vancomycin-sensitive *Enterococcus faecalis* (VSE) strain NCTC 755, vancomycin-resistant *E. faecalis* (VRE) strain NCTC 12201, and vancomycin-resistant *Enterococcus faecium* (VRE) strain NCTC 12204. The results are presented in Table 42. Compound **169**, characterized by its standard orientation, exhibited remarkable antibacterial efficacy, with MIC values of 0.125 µg/mL against all assessed Gram-positive strains. In contrast, compound **171**, characterized by its inverted amide bond orientation, exhibited a significant reduction in activity across all bacterial strains, particularly evident in MRSA strains, where MIC values exceeded 32 µg/mL. Notably, compound **172**, characterized by its inversion of both amide bonds linked to the *N*-methylpyrrole ring, exhibited enhanced antibacterial activity compared to compound **171**. However, its activity was inferior to that of compound **169**. It is worth considering that the assessment of the orientation’s impact on antibacterial activity is at the forefront of our analysis.

**Figure 52:**
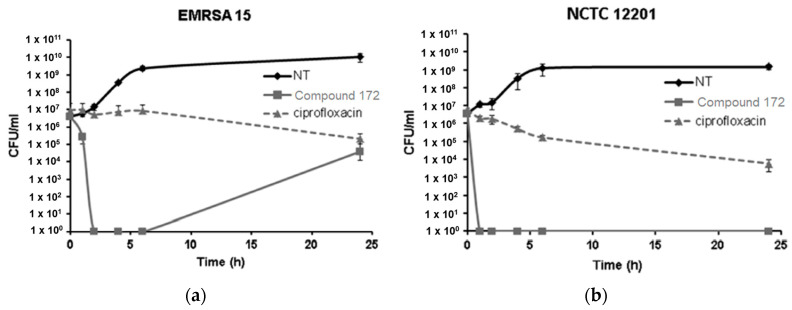
Mechanism of action of compound **172** against two bacterial strains: (**a**) EMRSA-15 and (**b**) NCTC 12201. This is an open-access article distributed under the terms of the Creative Commons CC BY license [65].

The mechanism of action of compound **172** was investigated via time–kill assays conducted on MRSA (EMRSA-15) and VRE (NCTC 12201) strains. These strains were exposed to compound **172** at a concentration of 4 × MIC for 24 h (Figure 52). Compound **172** demonstrated a rapid and robust bactericidal effect, leading to a reduction in cell counts below the detectable limit within a 2 h period. In contrast, ciprofloxacin exhibited bacteriostatic activity (Figure 52). Despite a modest cell population persisting in EMRSA-15 after 24 h of exposure to compound **172**, it exhibited no resistance to the compound, suggesting the presence of a potential persister population. This trend was more pronounced in EMRSA-15 than in NCTC 12201, underscoring the potential role of fluoroquinolone resistance.

**Figure 53:**
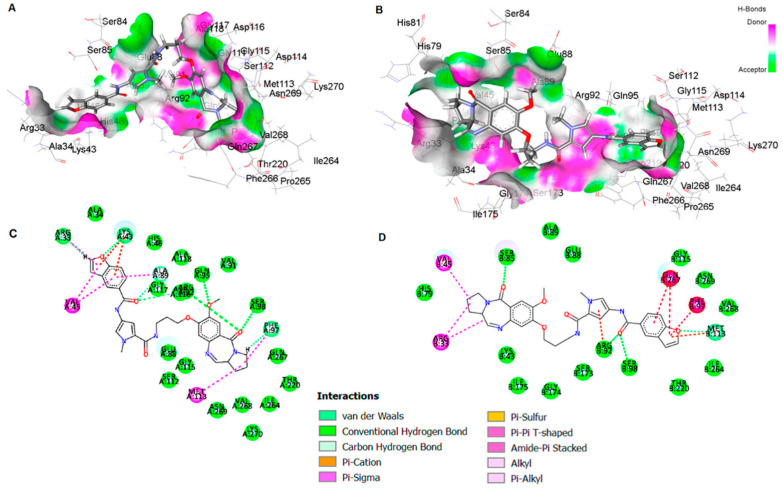
Molecular model depicting the interaction between compound **172** and (**A**) subunit 1 and (**B**) subunit 2 of the gyrase A in *Staphylococcus aureus* (PDB ID code: 2XCT). 2D model of the interaction of compound **172** with (**C**) subunit 1 and (**D**) subunit 2 of gyrase A in *Staphylococcus aureus*. This is an open-access article distributed under the terms of the Creative Commons CC BY license [65].

Moreover, an extensive in silico analysis was performed, employing ESI mutagenesis to unravel the mechanism of action inherent in the PBD derivatives. The gathered data unveiled that compound **172** exhibited a distinct interaction pattern with the ligand-binding domain of DNA gyrase, showcasing a notably robust binding affinity for both subunits of the bacterial DNA gyrase complex. The visualization of these interactions involving the bacterial gyrase from *Staphylococcus aureus* (PDB ID code: 2XCT) is visually presented in Figure 53A,B. As delineated in the 2D patterns depicted in Figure 53C, it becomes apparent that compound **172** forms three conventional hydrogen bonds, establishing connections with serine 98, arginine 92, and glutamine 95 within DNA gyrase A subunit 1. Analogously, the interaction extends to subunit 2 of DNA gyrase A, where it interacts with serine 85, arginine 92, and serine 98 (Figure 53D). The implications of the interaction underscore that the antibacterial activity attributed to compound **172** stems from its direct modulation of gyrase A via enzyme interaction, as opposed to its ability to stabilize DNA.

**Scheme 28:**
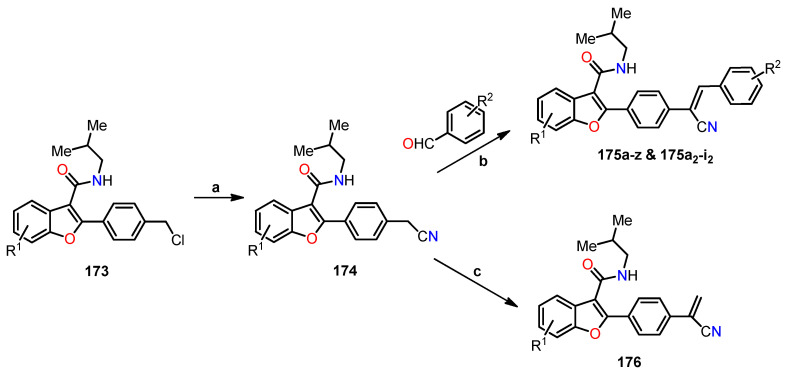
Synthesis of benzo[*b*]furan cyanide derivatives **175a**–**z**, **175a_2_**_–_**i_2_**, and **176**. Reagents and conditions: (**a**) (Me)_3_Si-CN, TBAF, MeCN, reflux, 2 h, 79–85%; (**b**) EtONa, EtOH, reflux, 1 h, 40–87%; (**c**) formaldehyde, EtONa, EtOH, reflux, 1 h.

Considering the significance of Sortase A (SrtA) as a cysteine transpeptidase prevalent in most Gram-positive bacteria, its pivotal role in the infection process of these organisms is well-established. Inhibition of this enzyme has been shown to exert a discernible impact on the virulence of Gram-positive bacteria, thereby rendering them more resistant to antibiotics. Acknowledging this premise, Lei et al. embarked upon extending these insights to *Staphylococcus aureus* (*S. aureus*). Given its susceptibility to detection and elimination by the immune system due to its lower viscosity, inhibiting SrtA emerged as a pertinent strategy [66]. To this end, a series of derivatives of 2-(4-(1-cyano-2-phenylvinyl)phenyl)-*N*-isobutylbenzofuran-3-carboxamide **175a**–**z**, **175a_2_**–**i_2_**, and **176** were synthesized from intermediates **173** [67], through substitution reactions involving cyanide, the compounds **174** were obtained (Scheme 28). Ultimately, via condensation reactions with diverse aldehydes, the cyano derivatives of benzo[*b*]furan **175a**–**z**, **175a_2_**–**i_2_**, and **176** were successfully generated.

**Scheme 29:**
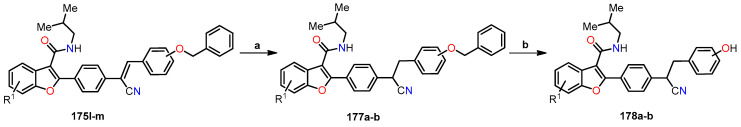
Synthesis of benzo[*b*]furan cyanide derivatives **177a**–**b** and **178a**–**b**. Reagents and conditions: (**a**) Pd/C, H_2_, MeOH, r.t, 1 h, 41–80%; (**b**) Pd/C, H_2_, MeOH, r.t, 12 h, 37–40%.

Additionally, three benzofuran-3-carboxamide derivatives were synthesized to evaluate the effect of the olefin cyanide group in inhibiting SrtA activity (Scheme 29). For this, a double reduction was carried out using Pd/C under a hydrogen atmosphere to reduce the olefinic double bond adjacent to the cyanide, followed by the removal of the benzyl group, thus obtaining the compounds **177a**–**b** and **178a**–**b**, respectively.

A comprehensive synthesis of 39 benzofuran cyanide derivatives was conducted, followed by their rigorous evaluation for in vitro inhibitory potential against SrtA in *S. aureus*, with pHMB serving as a positive control. The findings, detailed in Table 43, underscored the noteworthy activity exhibited by most of the synthesized analogs, with IC_50_ values spanning the range of 3–100 μM. Among these, compounds **175a**, **175e**, **175g**, **175i**, **175m**–**o**, **175w**, and **175h_2_** emerged as particularly significant performers. This performance differential might be attributed to the intricate interplay of structure–activity relationships, favoring the potency of analog **175a** (R^1^ = H, R^2^ = H, IC_50_ = 8.8 μM) over counterparts **175p** (R^1^ = 7-OMe, R^2^ = H, IC_50_ = 11.9 μM) and **175z** (R^1^ = 5-Cl, R^2^ = H, IC_50_ = 29.9 μM), primarily due to the strategic introduction of a substituent within the benzo[*b*]furan ring. The influence of R^2^ substitution on inhibitory activity was distinctly pronounced, as evidenced by the superiority of electron-withdrawing groups over donor groups, **175b** (R^1^ = H, R^2^ = 4-Me) vs. **175i** (R^1^ = H, R^2^ = 4-Cl), **175q** (R^1^ = 7-OMe, R^2^ = 4-Me) vs. **175w** (R^1^ = 7-OMe, R^2^ = 4-Cl), and **175b_2_** (R^1^ = 5-Cl, R^2^ = 3,4-Me) vs. **175h_2_** (R^1^ = 5-Cl, R^2^ = 4-Cl). Furthermore, the size of substituents emerged as another pivotal determinant, favoring the chlorine group in compounds **175i**, **175w**, and **175h_2_** (IC_50_ = 9.8, 5.9, and 6.8 μM, respectively), in contrast to the bromide group in compounds **175j** (R^1^ = H, R^2^ = 4-Br), **175x** (R^1^ = 7-OMe, R^2^ = 4-Br), and **175i_2_** (R^1^ = 5-Cl, R^2^ = 4-Br) with IC_50_ values of 19.1, 15.5, and 16.4 μM, respectively.

**Figure 54:**
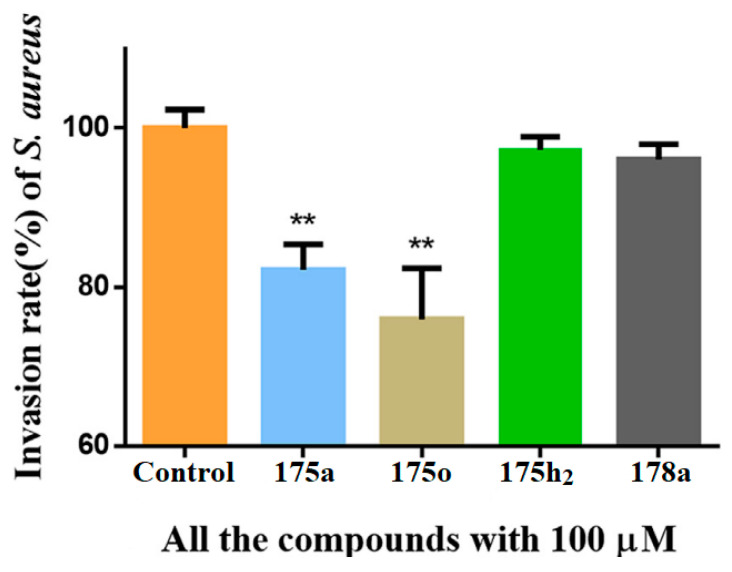
Quantitative analysis of *Staphylococcus aureus* invasion in 293T cells using flow cytometry. The evaluation encompasses the effect of compounds **175a**, **175o**, **175h_2_**, and **178a** on the invasion process. Data are reported as mean ± SD of at least three independent experiments performed in duplicate. ** *p* < 0.05 vs. control. Reproduced with permission from ref. [66]. Copyright Elsevier Inc., 2023.

Furthermore, the selection process extended to the identification of four compounds **175a**, **175o**, **175h_2_**, and **178a** boasting the most noteworthy SrtA inhibition activity. Subsequently, the impact of these compounds on impeding the formation of bacterial biofilms, a significant contributor to drug resistance, was diligently evaluated. To facilitate this assessment, SPSS Software was harnessed to compute the inhibitory potential of the chosen compounds, as outlined in Table 44. The findings underscored the inhibitory efficacy of all four compounds against the development of *S. aureus* biofilms, with IC_50_ values spanning a range of 2.1–54.2 μM. Remarkably, compound **175o** emerged as the most potent inhibitor, displaying an impressive IC_50_ of 2.1 μM. These results harmoniously align with the outcomes derived from the SrtA inhibition study. In addition, a further assay was performed to investigate the potential of the four compounds to disrupt the invasion of 293T cells (human embryonic kidney cells) by *S. aureus*, utilizing a group of drug-free FITC-labeled bacteria as the control reference. Intriguingly, all four compounds demonstrated a pronounced reduction in the invasiveness of *S. aureus* strains within 293T cells. Notably, compound **175o** exhibited the most substantial interference, diminishing bacterial invasion by a noteworthy 24.0% at 100 μM, as contrasted with the control blank (Table 44 and Figure 54).

### 2.3. Antifungal Activity

Furthermore, benzo[*b*]furans have demonstrated pronounced antifungal efficacy against a diverse spectrum of fungal strains, encompassing *Candida albicans* (*C. albicans*), *Candida neoformans* (*C. neoformans*), *Candida parapsilosis* (*C. parapsilosis*), *Aspergillus flavus* (*A. flavus*), and *Microspora griseus* (*M. griseus*)*,* among others. A seminal study by Sastraruji et al. has yielded a pivotal series of alkaloids **127a**–**i** extracted from *Stemona aphylla*, meticulously documented in Table 30 [58]. Within this set of nine isolated alkaloid derivatives, a judicious selection of five underwent rigorous examination to assess their antifungal prowess, with Amphotericin B employed as a comparative benchmark. Strikingly, the results unveiled that compounds **127g** (R^1^ = H, R^2^ = OMe, R^3^ = Me, R^4^ = Me, R^5^ = H, R^6^ = H) and **127i** (R^1^ = H, R^2^ = OMe, R^3^ = Me, R^4^ = Me, R^5^ = Me, R^6^ = H) exhibited performance tantamount to Amphotericin B against *C. albicans*, each recording an MIC value of 15.6 µg/mL. In contrast, with regard to *C. neoformans*, none of the evaluated alkaloids surpassed the efficacy of the control drug. Noteworthily, compounds **127a**, **127c**, and **127g** emerged as the exclusive contributors to antifungal activity against the *C. neoformans* strain, with recorded MIC values of 7.8 µg/mL (Table 45).

In a subsequent study conducted in 2013, Ostrowska et al. synthesized a series of chlorinated 2- and 3-benzo[*b*]furan carboxylates (Scheme 21 and Table 34). These derivatives underwent meticulous in vitro assessments to gauge their antimicrobial efficacy against both bacterial strains, as previously discussed, and fungal strains [60,61]. In terms of antifungal activity, they were scrutinized across strains encompassing *Aspergillus brasiliensis*, *Candida albicans, Candida parapsilosis, Saccharomyces cerevisiae*, and *Zygosaccharomyces rouxii*. The reference benchmark was fluconazole, spanning a range of MIC values from 3.9 × 10^−4^ to 8.4 × 10^−1^ µmol/cm^3^. The outcomes, as presented in Table 46, align with the antibacterial findings (Table 35), underscoring that the most potent derivative across all tested fungal strains was the compound **133f.HCl** (R^1^ = COOMe, R^2^ = H, R^3^ = Br, R^4^ = OMe, R^5^ = O(CH_2_)_2_NEt_2_).

In a similar vein, Kenchappa et al. embarked on the synthesis of a series of (5-substituted-1-benzofuran-2-yl)(2,4-phenyl-substituted)methanone derivatives **139a**–**i** (Scheme 22), which were subsequently subjected to comprehensive evaluation for their antifungal potential [62]. The outcomes are meticulously presented in Table 47, revealing noteworthy observations. Specifically, compounds **139a** (R^1^ = H, R^2^ = Br, R^3^ = H) and **139c** (R^1^ = Br, R^2^ = Br, R^3^ = H) exhibited a comparably potent activity to fluconazole (control drug) across all tested fungal strains, displaying MIC values ranging from 6.30 to 12.75 µg/mL. Similarly, compounds **139e** (R^1^ = OH, R^2^ = Br, R^3^ = H) and **139h** (R^1^ = Br, R^2^ = H, R^3^ = Br) demonstrated robust antifungal efficacy, recording MIC values spanning from 6.40 to 13.70 µg/mL. In contrast, compounds **139d** and **139g** showcased minimal activity against the tested fungal strains.

In the same year, Wang et al. undertook the synthesis of a series of analogs derived from naftifine hydrochloride (NFT), specifically designed to target the CrtN enzyme for the treatment of MRSA infections [64]. Among this array, compound **144l** emerged as the most promising analog (Table 39). Recognizing NFT’s established antifungal properties, a comparative analysis was conducted, gauging whether compound **144l** exhibited analogous effects. The outcomes, as presented in Table 48, unveiled that the compound **144l** analog displayed modest antifungal activity across all tested fungal strains, including *Trichophyton rubrum*, with MIC values reaching 16 µg/mL (128 times less potent than NFT). This variance in efficacy might be attributed to the structural difference between the benzofuran core of **144l** and the naphthalene moiety of NFT.

### 2.4. Analysis of the Structure–Activity Relationship in Anticancer Results

**Figure 55:**
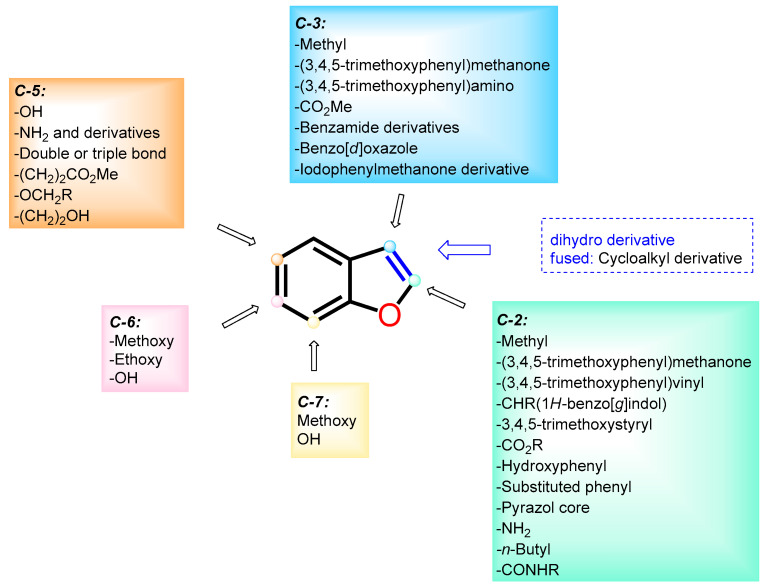
Analysis of the structure–activity relationship in anticancer results.

Interactions within the *α*- and *β*-tubulin structure have surfaced as a remarkable topic for exploring novel chemotherapeutic agents. Among the key domains, the colchicine binding site has emerged as a pivotal target for potential inducers of destabilization in tubulin polymerization. Colchicine binding site inhibitors (CBSIs) exert their biological impact by impeding tubulin assembly and limiting microtubule formation. In this context, benzo[*b*]furans have taken center stage, being designed and synthesized into a wide range of molecules interacting with the colchicine binding site, imparting marked structural diversity. Many compounds in this review demonstrated anticancer properties due to their inhibition of tubulin polymerization, achieved primarily through binding to the colchicine binding site on tubulin [32,34,37,39,40,43,46,49,51] (Figure 55). This is mainly achieved by the incorporation of the characteristic recognition fragment analogous to colchicine at positions C–2 or C–3 of benzo[*b*]furan, (3,4,5-trimethoxyphenyl)methanone [32,34,43,46,51], and their corresponding analogs [37,39,40], which interact through hydrogen bonds between methoxy groups and Cys241 as well as van der Waals interactions between phenyl ring and aromatic residues at the colchicine binding site on tubulin [40]. On the other hand, the methyl group located at positions C–2 or C–3 occupies hydrophobic pockets, while the necessity of hydrogen bonding interactions of methoxy or hydroxyl groups at positions C–6 and C–7 with the tubulin structure is evident. In particular, the hydrogen bonding interaction of the methoxy group at C–6 with Asnβ258 has remarkable significance [32,39]. This interaction not only enhances the antiproliferative activity in breast, renal, and melanoma cancer cell lines [36,46] but also contributes to the suppression of breast cancer metastasis [42]. Furthermore, additional hydrogen bonding interactions with donating groups like amino, hydroxyl, and unsaturations at C–5, along with alkyl ester derivatives [42] and alkoxy derivatives [45], play a role in stabilizing the active site of *α*- and *β*-tubulin [32], leading to the inhibition of different cancer cell lines [36]. Regarding substitution at position C–2, the diversity of functional groups allows the formation of hydrogen bonding interactions at active sites, and they also encompass aromatic groups that possess the ability to engage in π–π stacking with aromatic residues [34,37,38,39,42,43,46,48]. Notably, modifications such as the loss of aromaticity in the furan ring and the fusion of aliphatic chains on the *d*-side of benzo[*b*]furan enable the maintenance of activity against diverse cancer cell lines. 

Some benzo[*b*]furan derivatives discussed in this review have undergone comprehensive studies analyzing their impact on the apoptotic pathway of cell death across various cell lines, including nuclear condensation [37], increased activation of caspase-3 [37,48,49,52], down-regulation of procaspase-9 [41,49], and negative regulation of the Bcl-2 protein family [37,40,41,48,49,52], as well as Bcl-x expression [54], PARP-1 cleavage [48,49], phosphorylation and stabilization of p53 [37,48,50], and a reduction in the expression of Bubr1 and Mad-2 proteins [51], among other effects. Furthermore, some benzo[*b*]furan derivatives demonstrated anti-vascular properties [40,51], a significant aspect in tumoral angiogenesis. Several researchers have confirmed the predominant cell cycle arrest in the G2/M phase [37,41,44,46,48,49,51]. Notably, effects on the PI3K/Akt/mTOR signaling pathway have been observed [41], along with the inhibition of Top 1 DNA relaxation activity [52], suppression of SIRT3 histone deacetylase [53], mitigation of tumor cell metastasis as demonstrated in in vivo mouse assays [42], facilitation of the tumor resection process via bioluminescence monitoring [42,51,52,53], and anti-metastatic effects underscored by interleukin-25 secretion from tumor-associated fibroblasts (TAF) [42]. In terms of innovation, this review highlights the advancement of novel compounds through the utilization of comparative molecular field analysis (CoMFA) and comparative molecular similarity indices analysis (CoMSIA) methodologies [43]. What is particularly remarkable is the versatility exhibited by benzo[*b*]furans, which extends to the exploration of triple-negative breast cancer [52,53], an exceptionally aggressive cancer type known for its rapid recurrence and challenging prognosis.

### 2.5. Analysis of the Structure–Activity Relationship in Antibacterial and Antifungal Results

To date the capacity to regulate the proliferation of bacteria and fungi holds considerable significance. The minimum inhibitory concentration (MIC) enables the identification of chemical structures capable of effectively managing visible growth inhibition in vitro against bacterial and fungal strains. Notably, the investigation into novel compounds possessing antibacterial activity is one of the most significant focuses within the microbial field, owing to the emergence of extensive resistance to conventionally employed antibacterial drugs.

**Figure 56:**
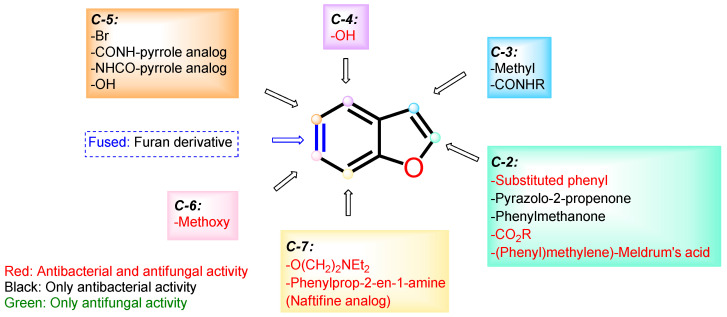
Analysis of the structure–activity relationship in antibacterial and antifungal results.

In this review, the adaptability and versatility of benzo[*b*]furans toward both Gram-positive and Gram-negative bacteria are evident. This adaptability is apparent in the various structural modifications involving the incorporation of different functional groups at the six carbons of the structure (Figure 56). Beginning with the former, significant activity was observed against Gram-positive bacteria, including *methicillin-resistant Staphylococcus aureus* (MRSA) [58,64,65], *Staphylococcus aureus Newman* [64], *Staphylococcus aureus* [59,61], *Bacillus subtilis* [59,61,62], *Micrococcus luteus* [61], *Bacillus cereus* [61], *Staphylococcus epidermidis* [61], *Enterococcus hirae* [61], *vancomycin-sensitive Enterococcus faecalis* (VSE) [65], *vancomycin-resistant Enterococcus faecalis* (VRE) [65], and *vancomycin-resistant Enterococcus faecium* (VRE). Additionally, Gram-negative bacteria were targeted, including *Escherichia coli* [59,61,62], *Pseudomonas aeruginosa* [59,61], *Pseudomonas syringae* [62], *Salmonella typhi* [62], and *Klebsiella pneumoniae* [62]. Furthermore, enzymatic assays were performed against typical *Staphylococcus aureus* enzymes, specifically 4,4′-diapophytoene desaturase (CrtN) [64] and cysteine transpeptidase Sortase A (SrtA) [66]. Moreover, dose–response analyses of benzo[*b*]furans against pigment formation in various bacterial strains were performed [64]. In addition, in vivo assays were conducted to evaluate virulence in mice through retro-orbital injection [64], while incidence measurements were taken in organs such as the kidney and heart [64]. The potential of benzo[*b*]furans to bind to DNA and provide stability was investigated using the Förster resonance energy transfer (FRET) technique [65]. Additionally, in silico analyses were performed utilizing ESI mutagenesis in gyrase A in *Staphylococcus aureus* [65]. Among other tests, the disruption of invasion in 293T cells (human embryonic kidney cells) was also examined [66]. It is crucial to emphasize that, in some cases, the benzo[*b*]furans compiled in this review exhibit superior activity when compared to reference antibacterial drugs, such as Chloramphenicol [59], Carbenicillin [59], Streptomycin [59,62], Tetracycline [59], Netropsin [65], Ciprofloxacin [65], and polyhexamethylene biguanide (pHMB) [66].

Conversely, a significant number of antibacterial compounds also demonstrate antifungal properties. Just like their antibacterial counterparts, the demand for novel compounds is pressing due to the emergence of multiple resistances. This review reveals that at a structural level, numerous benzo[*b*]furan derivatives exhibit both antibacterial and antifungal properties, as illustrated in Figure 56. The fungal strains examined within the studies discussed in this review displayed significant efficacy against *Candida albicans* (*C. albicans*) [58,61], *Candida neoformans* (*C. neoformans*) [58], *Candida parapsilosis* (*C. parapsilosis*) [61], *Saccharomyces cerevisiae* (*S. cerevisiae*) [61], *Zygosaccharomyces rouxii* (*Z. rouxii*) [61], *Aspergillus flavus* (*A. flavus*), *Microspora griseus* (*M. griseus*), and *Trichophyton rubrum* (*T. rubrum*) [64]. Notably, in some cases, their antifungal activity is comparable to reference drugs, such as Amphotericin B [58], Fluconazole [62,64], Voriconazole [64], and Ketoconazole [64].

## 3. Conclusions

Benzo[*b*]furans play a significant role in drug discovery and medicinal chemistry due to their unique structural features and wide range of biological and pharmaceutical properties. Most of the synthetic approaches for the synthesis and functionalization of these bioactive compounds involve multistep sequences. This comprehensive review provides a detailed overview of the synthesis and biological studies conducted on benzo[*b*]furans from 2011 to 2022, mainly focusing on their potential as anticancer, antibacterial, and antifungal agents. The selected articles provide invaluable insights related to chemical synthesis, discussions on molecular docking, and both in vitro and in vivo studies. By underscoring the importance of comprehending mechanisms of action, which encompass important processes such as inhibiting the cell cycle, inducing tumor regression, and demonstrating potent antibacterial and antifungal effects. Notably, the anticancer activity of benzo[*b*]furans correlates with their binding to tubulin’s colchicine site, leading to polymerization inhibition. Colchicine-like fragments on specific positions of benzo[*b*]furan engage in interactions that impede cancer cell growth and metastasis. These compounds also influence apoptotic pathways, activate caspases, regulate Bcl-2, stabilize p53, affect angiogenesis, impact cell cycle and signaling pathways, modulate DNA activity, and influence metastasis in vivo. Conversely, benzo[*b*]furans exhibited a dual effectiveness against antibacterial and antifungal infections. In particular, benzo[*b*]furans exhibited significant efficacy against Gram-positive and Gram-negative bacterial strains, positioning them as potential superior antibacterial agents compared to reference drugs. These findings hold utmost importance in propelling scientific research and drug discovery, possibly paving the way for novel and potent therapeutic agents containing the benzo[*b*]furan ring. 

## Figures and Tables

**Table 1:** Anticancer evaluation of benzo[*b*]furans **4**–**6**.

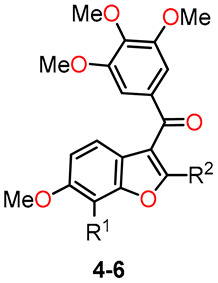							
Compound	R^1^	R^2^	Tubulin ^a^ IC_50_ (µM)	MCF-7 ^b^ IC_50_ (µM)	Activated HUVEC ^c^ IC_50_ (µM)	Quiescent HUVEC ^d^ IC_50_ (µM)	Selectivity Ratio ^e^
CA-4 ^f^	-	-	1.8 ± 0.2	2.9	3.6	3.9	1.1
**4a**	H	H	1.6 ± 0.2	55 ± 5	45	63	1.4
**4b**	OH	H	ND	45 ± 15	83	322	3.9
**5a**	H	Br	ND	495 ± 25	510	309	0.6
**6a**	OH	Me	3.0 ± 0.6	2.4 ± 2	0.31	25	81
**6b**	H	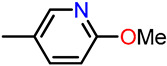	ND	71 ± 1	45	27	0.6
**6c**	H		ND	215 ± 15	485	405	0.8
**6d**	OH	2-Furanyl	8.7 ± 0.8	0.6 ± 0.2	3.6	3.4	0.9
**6e**	OH	2-Thiophenyl	ND	0.5 ± 0.2	0.31	0.36	1.2
**6f**	OH	Pyrrole	ND	ND	2.6	3.3	1.3
**6g**	OH	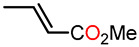	ND	ND	0.38	2.3	6.0
**6h**	OH	CN	ND	ND	1.1	3.0	2.7
**6i**	OH	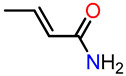	ND	ND	2.6	2.3	0.9
**6j**	OH	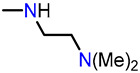	ND	ND	29	36	1.2
**6k**	OH	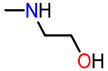	ND	ND	3.1	2.9	0.9
**6l**	OH	NH_2_	ND	ND	1.9	8.8	4.6

^a^ The tubulin concentration used was 10 μM. Inhibition of extent of assembly was the parameter measured (*n* = 2). ^b^ Cells were cultured for 48 h at 37 °C in a humidified atmosphere containing 5% CO_2_. The data presented are the mean ± SD of two independent experiments. ^c^ HUVEC cells were seeded at 2500 and 500 cells/well and cultured in EGM-2 (Lonza) or F12K medium containing 0.03 mg/mL endothelial cell growth supplement. ^d^ HUVEC and HAAE-1 cells were seeded at 15,000 and 5000 cells/well, respectively, in basal medium (EBM-2 or F12K) containing 0.5% fetal calf serum and antibiotics. ^e^ Selectivity ratio (IC_50_ quiescent)/(IC_50_ activated). ^f^ Combretastatin-A4 (CA-4) is the standard drug for the study. ND means not detected. Reprinted (adapted) with permission from ref. [32]. Copyright American Chemical Society, 2023.

**Table 2:** In vitro inhibition of cell proliferation in various cancer cell lines by **6a** and CA-4.

Entry	Cell Line ^a^	Cell Type	6a IC_50_ (nM)	CA-4 IC_50_ (nM)
1	Activated HAAE-1	Endothelial cell	0.1 (120) ^b^	2.2 (1.7) ^b^
2	Quiescent HAAE-1	Endothelial cell	12	1.3
3	U87-MG	Brain glioblastoma	0.41	2.6
4	DU145	Prostate carcinoma	0.36	4
5	Calu-6	Lung anaplastic carcinoma	0.16	0.94
6	MDA-MB-231	Breast adenocarcinoma	0.63	3.2
7	A431	Epidermoid carcinoma	18.6	188
8	A375	Malignant skin melanoma	1.5	2.1
9	SKOV-3	Ovary adenocarcinoma	0.59	5.5
10	LoVo	Colorectal adenocarcinoma	0.24	2.9
11	AU565	Breast adenocarcinoma	5.8	4.4
12	BT549	Breast carcinoma	0.34	3.5

^a^ HAAE-1 cells were seeded at 500 cells/well in medium containing 0.03 mg/mL endothelial cell growth supplement (activated) or in basal medium containing 0.5% fetal calf serum and antibiotics (quiescent). Cancer cell lines were seeded at an average of 500–2000 cells/well and cultured as recommended by the ATCC. ^b^ Selectivity ratio (IC_50_ quiescent)/(IC_50_ activated) in parentheses. Reprinted (adapted) with permission from ref. [32]. Copyright American Chemical Society, 2023.

**Table 3:** In vitro inhibitory effects of benzo[*b*]furans **10**, **11**, and CA-4 against the proliferation of murine leukemia (L1210), murine mammary carcinoma (FM3A), human T-lymphocyte (Molt/4 and CEM), and human cervix carcinoma (HeLa) cells.

Compound	IC_50_ (nM) ^b^				
	L1210	FM3A/0	Molt4/C8	CEM/0	HeLa
**10a**	6700 ± 600	9200 ± 800	ND ^c^	5400 ± 1200	2200 ± 600
**10b**	>10,000	>10,000	>10,000	>10,000	>10,000
**10c**	2500 ± 100	3600 ± 150	ND	2400 ± 400	5500 ± 530
**10d**	>10,000	>10,000	>10,000	>10,000	7500 ± 200
**10e**	>10,000	>10,000	>10,000	>10,000	>10,000
**10f**	>10,000	>10,000	>10,000	>10,000	>10,000
**10g**	73 ± 24	73 ± 2	66 ± 6.7	59 ± 4.1	42 ± 3.8
**10h**	19 ± 2	24 ± 6	22 ± 4	22 ± 5	16 ± 1
**10i**	600 ± 90	560 ± 90	870 ± 20	450 ± 10	370 ± 30
**10j**	67 ± 4	140 ± 13	360 ± 20	120 ± 10	490 ± 54
**11a**	78 ± 2.8	200 ± 90	ND	110 ± 20	1000 ± 80
**11b**	150 ± 8	320 ± 30	230 ± 1	390 ± 30	1100 ± 90
**11c**	320 ± 17	520 ± 29	ND	310 ± 80	430 ± 90
**11d**	420 ± 90	1200 ± 90	ND	600 ± 37	1200 ± 80
**11e**	800 ± 51	780 ± 62	1100 ± 90	970 ± 55	1000 ± 100
**11f**	1200 ± 40	5900 ± 380	ND	3200 ± 240	5900 ± 900
**11g**	430 ± 23	960 ± 78	ND	340 ± 20	1000 ± 100
**11h**	120 ± 14	250 ± 15	300 ± 40	240 ± 38	780 ± 54
**11i**	390 ± 20	1100 ± 100	920 ± 91	770 ± 15	1400 ± 120
**13**	14 ± 1	10 ± 8	18 ± 1	20 ± 2	14 ± 1
CA-4 ^a^	2.8 ± 1.1	42 ± 6.0	16 ± 1.4	1.9 ± 1.6	1.9 ± 1.6

^a^ Combretastatin-A4 (CA-4) is the standard drug for the study. ^b^ The data presented are the mean ± SD of three independent experiments. ^c^ ND means not detected. Reproduced with permission from ref. [34]. Copyright John Wiley & Sons Inc., 2023.

**Table 4:** Reaction conditions for the synthesis of dihydroxylated 5,6-benzo[*b*]furans **16a**–**n**.

Entry	Catechol Derivative	1,3-Dicarbonyl Compound	Reaction Time (h)	Method	Yield 16 (%)
1	**14a**	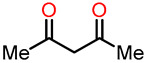	24	A	**16a** (48)
2	**14a**		44	B	**16a** (49)
3	**14b**		24	A	**16b** (50)
4	**14a**	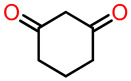	24	A	**16c** (65)
5	**14b**		24	A	**16d** (62)
6	**14b**		44	B	**16d** (67)
7	**14c**		24	A	**16e** (70)
8	**14a**	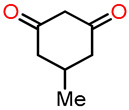	24	A	**16f** (59)
9	**14a**		42	C	**16f** (50)
10	**14b**		24	A	**16g** (78)
11	**14b**		42	C	**16g** (71)
12	**14c**		24	A	**16h** (37)
13	**14a**	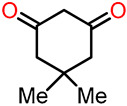	24	A	**16i** (58)
14	**14a**		42	C	**16i** (40)
15	**14b**		24	A	**16j** (98)
16	**14b**		42	C	**16j** (59)
17	**14c**		24	A	**16k** (73)
18	**14c**		44	B	**16k** (77)
19	**14a**	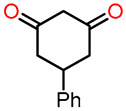	24	A	**16l** (76)
20	**14a**		42	C	**16l** (50)
21	**14b**		24	A	**16m** (80)
22	**14c**		24	A	**16n** (43)
23	**14c**		42	C	**16n** (15)

**Table 5:** The cytotoxicity against a panel of cancer cell lines and the inhibition of tubulin polymerization of compounds **22**, **25**, and CA-4.

Compound	IC_50_ (µM) ^b^					Tubulin	
	ME-180	A549	ACHN	HT-29	B-16	IC_50_ (µM) ^c^	%
**22**	0.19 ± 0.02	0.08 ± 0.16	0.97 ± 0.49	0.08 ± 0.10	1.14 ± 0.50	3.81 ± 0.4	37.9
**25**	0.09 ± 0.02	0.06 ± 0.11	0.09 ± 0.14	0.08 ± 0.01	0.17 ± 0.09	1.95 ± 0.1	65.4
CA-4 ^a^	0.007 ± 0.06	0.05 ± 0.06	0.09 ± 0.08	0.008 ± 0.01	0.06 ± 0.01	1.86 ± 0.1	70.5

^a^ Combretastatin-A4 (CA-4) is the standard drug for the study. ^b^ Values are the mean ± SD of three independent experiments determined after 48 h of treatment. ^c^ Values are the mean ± SD of two independent experiments performed in triplicate.

**Table 6:** Synthesis of diheteroarylalkanal **29** through a one-pot Friedel–Crafts reaction.

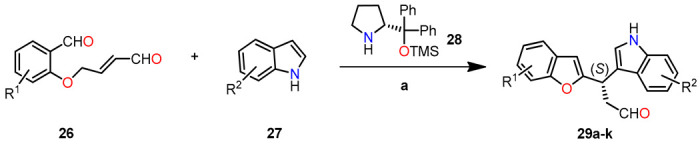				
Compound	R^1^	R^2^	Yield (*S*)-29 (%)	*% ee*
**29a**	H	H	79	94
**29b**	H	5-Br	71	95
**29c**	H	5-Ome	75	96
**29d**	H	2-Me	79	99
**29e**	H	7-Me	78	96
**29f**	H	benzo[*g*]	85	98
**29g**	5-Cl	H	76	97
**29h**	5-Me	H	72	94
**29i**	5-NO_2_	H	75	93
**29j**	7-Ome	H	71	93
**29k**	6-Ome	H	69	94

Reagent and conditions: (**a**) 26 (0.1 mmol), 27 (0.12 mmol), 28 (0.02 mmol), DABCO (0.02 mmol), and toluene (0.3 mL).

**Table 7:** Synthesis of (*Z*)-benzo[*b*]furan-2-yl and (*Z*)-benzo[*b*]furan-3-yl cyanocombretastatins **32a**–**f** and the evaluation of their anti-leukemic activity (LD_50_) against the MV4–11 AML cell line.

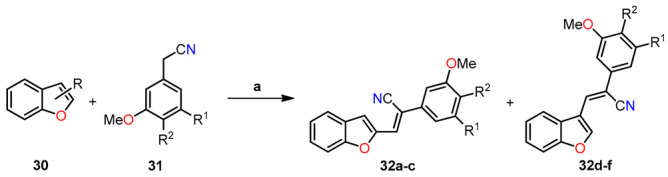				
Compound	R	R^1^	R^2^	LD_50_ (µM)
**32a**	2-CHO	OMe	OMe	0.047
**32b**	2-CHO	H	OMe	>20
**32c**	2-CHO	OMe	H	1.169
**32d**	3-CHO	OMe	OMe	4.529
**32e**	3-CHO	H	OMe	ND
**32f**	3-CHO	OMe	H	ND

Reagent and conditions: (**a**) 30 (1.0 mol), 31 (1.1 mol), NaOMe (5%), CH3OH, reflux, 3–6 h. ND means not detected.

**Table 8:** In vitro cell growth inhibitory effects of compounds **35a**–**l** and CA-4 on various cancer cell lines.

Compound	IC_50_ (nM) ^b^						
	HeLa	A549	HT-29	Jurkat	RS 4; 11	MCF-7	HL-60
**35a**	260 ± 50	5280 ± 800	930 ± 35	4100 ± 200	430 ± 97	7800 ± 900	4400 ± 200
**35b**	1330 ± 580	5470 ± 700	1600 ± 120	180 ± 38	300 ± 80	6600 ± 310	2500 ± 130
**35c**	>10.000	>10.000	>10.000	>10.000	>10.000	>10.000	>10.000
**35d**	>10.000	>10.000	>10.000	>10.000	>10.000	>10.000	>10.000
**35e**	250 ± 88	1570 ± 430	240 ± 60	210 ± 20	39 ± 9	7900 ± 1300	470 ± 30
**35f**	1260 ± 510	8900 ± 1460	1400 ± 540	2300 ± 700	190 ± 15	2900 ± 400	3400 ± 120
**35g**	2 ± 0.1	9 ± 1.4	3 ± 0.9	8 ± 0.6	0.3 ± 0.1	27 ± 2	5 ± 1
**35h**	13 ± 8	36 ± 11	17 ± 8	22 ± 6	100 ± 10	25 ± 3	24 ± 7
**35i**	130 ± 60	1270 ± 400	290 ± 30	30 ± 5	1 ± 0.1	520 ± 40	320 ± 17
**35j**	270 ± 80	1100 ± 300	110 ± 50	290 ± 50	230 ± 10	2100 ± 90	590 ± 50
**35k**	2530 ± 280	8900 ± 1300	3200 ± 210	3700 ± 450	400 ± 100	>10.000	4200 ± 200
**35l**	3280 ± 370	7250 ± 237	5300 ± 290	9100 ± 820	3000 ± 400	>10.000	5500 ± 540
CA-4 ^a^	4 ± 1	180 ± 30	3100 ± 100	5 ± 0.6	0.8 ± 0.2	370 ± 100	1 ± 0.2

^a^ Combretastatin-A4 (CA-4) is the standard drug for the study. ^b^ Data are expressed as the mean ± SE from the dose–response curves of at least three independent experiments.

**Table 9:** Antitubular and colchicine binding evaluation by compounds **35e**, **35g**–**j**, and CA-4.

Compound	Tubulin Assembly IC_50_ (µM) ^a^	Colchicine Binding % Inhibition ^b^
**35e**	7.5 ± 0.5	ND
**35g**	1.1 ± 0.1	83 ± 0.5
**35h**	1.5 ± 0.2	74 ± 4.1
**35i**	7.6 ± 1.0	ND
**35j**	6.4 ± 0.9	ND
CA-4 ^c^	1.1 ± 0.1	99 ± 0.1

^a^ Inhibition of tubulin polymerization: tubulin was used at 10 µM. ^b^ Inhibition of [3H] colchicine binding: tubulin, colchicine, and the tested compound were used at 1, 5, and 5 µM, respectively. ^c^ Combretastatin-A4 (CA-4) is the standard drug for the study. Values represent the mean ± SE from three independent experiments. ND means not detected.

**Table 10:** Antiproliferative activity of compounds **22** and **25**.

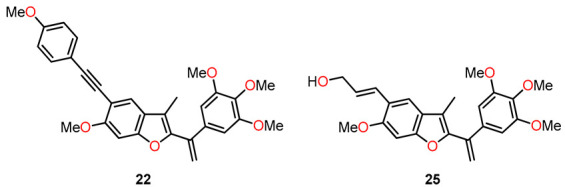		
Compound	IC_50_ (µM)	
	MCF-7	MDA MB-231
**22**	0.057 ± 0.008	0.168 ± 0.008
**25**	0.051 ± 0.002	0.093 ± 0.006

Data are presented as the mean ± SE from at least three independent experiments.

**Table 11:** Prediction of pIC_50_c values for compounds **43a**–**e**.

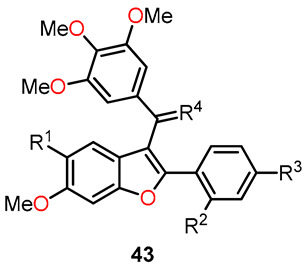						
Compound	R^1^	R^2^	R^3^	R^4^	pIC_50_c	
					CoMFA	CoMSIA
**43a**	H	H	OEt	O	0.351	0.291
**43b**	F	H	Ome	O	0.285	0.308
**43c**	H	Nme_2_	Ome	O	0.348	0.282
**43d**	H	Me	Ome	O	0.425	0.346
**43e**	H	NH_2_	Ome	CH_2_	0.503	0.206
CA-4 ^a^	-	-	-	-	0.000	0.037

^a^ Combretastatin-A4 (CA-4) is the standard drug for the study.

**Table 12:** Antiproliferative activities and inhibition of tubulin polymerization of compounds **43a**–**b**, CA-4, and CA-4P.

Compound	IC_50_ (µM) ^a^						Tubulin
	A549	HCT-116	HeLa	HepG2	MGC-803	MCF-7	IC_50_ (µM) ^a^
**43a**	1.37 ± 0.14	12.55 ± 0.60	8.99 ± 1.11	1.31 ± 0.17	9.97 ± 0.81	0.91 ± 0.16	0.86 ± 0.07
**43b**	6.87 ± 0.34	78.23 ± 3.83	19.1 ± 1.59	4.75 ± 0.20	47.26 ± 1.17	6.49 ± 0.55	>200
CA-4 ^b^	5.99 ± 0.46	9.00 ± 0.85	11.33 ± 1.03	16.04 ± 0.50	1.76 ± 0.85	0.67 ± 0.13	0.88 ± 0.22
CA-4P ^b^	0.40 ± 0.17	0.24 ± 0.05	0.56 ± 0.12	0.27 ± 0.04	0.47 ± 0.14	0.96 ± 0.22	4.79 ± 0.40

^a^ Data are expressed as the mean ± SE of at least three independent experiments. ^b^ Combretastatin-A4 (CA-4) and its disodium phosphate form (CA-4P) serve as the standard drugs for this study.

**Table 13:** Antiproliferative evaluation of compounds **51** and **52**.

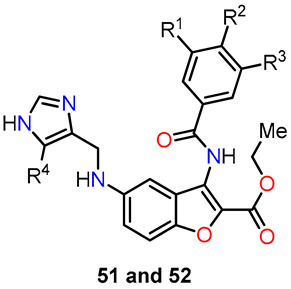						
Compound	R^1^	R^2^	R^3^	R^4^	GI_50_ (µM) ^a^	
					HeLa	MCF-7
**51a**	H	H	H	H	41.59 ± 3.08	49.65 ± 4.91
**51b**	H	Ome	H	H	7.98 ± 0.67	13.36 ± 1.94
**51c**	H	Me	H	H	13.65 ± 1.02	18.22 ± 1.28
**51d**	Ome	Ome	Ome	H	12.18 ± 1.19	3.88 ± 0.40
**51e**	H	CF_3_	H	H	>50	>50
**51f**	Cl	F	H	H	13.61 ± 0.86	11.25 ± 1.11
**52a**	H	H	H	Me	7.69 ± 0.46	12.27 ± 1.46
**52b**	H	Ome	H	Me	14.31 ± 1.97	17.35 ± 1.78
**52c**	H	Me	H	Me	9.20 ± 0.34	15.53 ± 0.98
**52d**	Ome	Ome	Ome	Me	12.19 ± 1.59	9.21 ± 1.02
**52e**	H	CF_3_	H	Me	2.01 ± 0.13	14.16 ± 2.02
**52f**	Cl	F	H	Me	2.14 ± 0.35	1.55 ± 0.14

^a^ Data are expressed as the mean ± SD of at least three independent experiments.

**Table 14:** IFD scores for compounds **51a**–**f**, **52a**–**f**, colchicine, and CA-4.

Compound	Prime Energy	Glide Score	IFD Score ^a^
**51a**	−34,437	−7.17	−1729
**51b**	−34,462	−10.51	−1734
**51c**	−34,487	−11.96	−1736
**51d**	−34,496	−12.23	−1737
**51e**	−34,480	−10.45	−1734
**51f**	−34,395	−11.78	−1732
**52a**	−34,463	−8.16	−1731
**52b**	−34481	−11.04	−1735
**52c**	−34,478	−10.38	−1734
**52d**	−34,545	−10.11	−1737
**52e**	−34,371	−10.53	−1729
**52f**	−34,475	−11.28	−1735
Colchicine	−34,392	−10.38	−1730
CA-4	−34,316	−9.17	−1725

^a^ Defined as: glide score + 0.05 prime energy.

**Table 15:** Antiproliferative evaluation of 1,2,4-oxadiazole fused benzo[*b*]furan derivatives **62a**–**j**.

Compound	IC_50_ (µM)			
	A549	MCF-7	A375	HT-29
**62a**	2.900	10.560	ND	3.78
**62b**	0.030	0.011	1.45	0.33
**62c**	1.900	0.400	2.01	0.18
**62d**	1.030	1.760	ND	1.22
**62e**	3.200	ND	ND	9.70
**62f**	10.400	2.440	5.70	ND
**62g**	0.100	0.014	ND	1.89
**62h**	0.012	0.015	1.09	0.17
**62i**	7.100	13.600	ND	20.50
**62j**	0.100	0.020	0.19	1.23
CA-4 ^a^	0.110	0.180	0.21	0.93

^a^ Combretastatin-A4 (CA-4) is the standard drug for the study. ND means not detected.

**Table 16:** Antiproliferative activity of benzo[*b*]furan derivatives against the A375 cancer cell line.

Compound	IC_50_ (µM) ^a^
**68b**	>100
**69a**	2.85 ± 0.82
**69b**	0.86 ± 0.37
**70**	0.09 ± 0.03
**72**	>100
**73**	>100

^a^ The data indicate the mean ± SD of at least three independent experiments.

**Table 17:** Antiproliferative activity of benzofuran–pyrazole hybrid **77** and its nanoparticles.

Compound	IC_50_ (nM) ^a^		
	MCF-7	MDA-MB-231	MCF-12A
**77**	7 ± 1	10 ± 1	87,600 ± 335
Nanoparticles—77	1 ± 0.4	0.6 ± 0.1	21,540 ± 66
Doxorubicin ^b^	620 ± 31	620 ± 31	ND

^a^ The data indicate the mean ± SD of at least three independent experiments. ^b^ Doxorubicin is the standard drug for the study. ND means not detected.

**Table 18:** Caspase-3, p53, Bax, and Bcl-2 levels of benzofuran–pyrazole hybrid **77** and its nanoparticles.

Compound	Cell Line	Caspasase-3	p53	Bax	Bcl-2
**77**	MCF-7	6.383836	7.453852	5.745321	0.272695
	MDA-MB-231	5.399087	7.792609	7.553853	0.181989
Nanoparticles—77	MCF-7	14.56524	12.51432	9.149760	0.131011
	MDA-MB-231	17.915	14.60536	13.19230	0.134738
Untreated control	MCF-7	1	1	1	1
	MDA-MB-231	1	1	1	1

**Table 19:** PARP-1 inhibitory assay of benzofuran–pyrazole hybrid **77** and its nanoparticles.

Compound	IC_50_ (nM) ^a^	
	MCF-7	MDA-MB-231
**77**	40 ± 1	60 ± 1
Nanoparticles—77	10 ± 4	6 ± 3
Staurosporine ^b^	10 ± 1	8 ± 1

^a^ The data indicate the mean ± SD of at least three independent experiments. ^b^ Staurosporine is the standard drug for the study.

**Table 20:** Antiproliferative activity of shikonin–benzo[*b*]furan hybrids **83a**–**q**, shikonin, colchicine, and CA-4.

Compound	IC_50_ (µM) ^a^					CC_50_ (µM) ^a^	
	HepG2	HT29	HCT116	MDA-MB-231	A549	293T ^b^	LO2 ^b^
**83a**	46.75 ± 6.96	7.16 ± 0.81	10.27 ± 1.60	6.00 ± 0.51	24.91 ± 2.29	169.08 ± 17.58	155.31 ± 9.20
**83b**	75.33 ± 3.23	1.22 ± 0.09	2.28 ± 0.32	1.91 ± 0.34	19.05 ± 1.83	ND	ND
**83c**	73.20 ± 4.03	0.18 ± 0.04	0.58 ± 0.11	0.81 ± 0.13	0.57 ± 0.79	184.86 ± 9.88	154.76 ± 9.98
**83d**	71.83 ± 5.19	7.62 ± 0.85	10.68 ± 0.18	8.22 ± 0.38	18.53 ± 1.43	158.90 ± 19.48	151.52 ± 8.23
**83e**	ND	6.06 ± 0.16	3.86 ± 0.47	3.59 ± 0.33	47.20 ± 2.24	ND	ND
**83f**	52.38 ± 5.27	1.03 ± 0.17	2.21 ± 0.40	2.63 ± 0.13	27.33 ± 1.34	162.38 ± 17.77	197.07 ± 9.88
**83g**	ND	10.37 ± 0.44	20.36 ± 1.82	10.42 ± 0.61	42.43 ± 5.35	170.07 ± 18.85	ND
**83h**	44.60 ± 4.88	5.89 ± 0.77	9.16 ± 0.44	4.09 ± 0.75	30.77 ± 2.11	158.61 ± 9.77	143.36 ± 9.88
**83i**	ND	0.82 ± 0.08	1.21 ± 0.07	1.51 ± 0.10	48.81 ± 5.66	ND	ND
**83j**	70.68 ± 6.96	4.04 ± 0.29	9.30 ± 0.76	10.74 ± 0.60	28.19 ± 2.53	194.98 ± 10.79	107.54 ± 9.93
**83k**	82.60 ± 9.68	9.14 ± 0.65	11.61 ± 0.91	4.88 ± 0.12	23.27 ± 2.65	121.92 ± 9.86	165.62 ± 11.98
**83l**	74.81 ± 8.50	7.55 ± 0.44	11.99 ± 1.21	15.25 ± 0.22	21.55 ± 3.26	148.46 ± 18.78	178.54 ± 8.50
**83m**	80.92 ± 9.13	16.35 ± 1.06	23.01 ± 0.97	18.33 ± 0.03	22.24 ± 3.83	149.38 ± 7.86	104.74 ± 9.85
**83n**	77.71 ± 5.81	1.12 ± 0.11	3.57 ± 0.50	2.01 ± 0.06	16.99 ± 0.66	ND	ND
**83o**	81.32 ± 8.76	0.73 ± 0.09	1.27 ± 0.34	1.33 ± 0.44	9.11 ± 2.21	ND	ND
**83p**	ND	ND	ND	24.68 ± 0.56	ND	ND	ND
**83q**	90.78 ± 9.73	10.94 ± 0.90	11.28 ± 0.94	6.21 ± 0.03	19.13 ± 1.89	108.40 ± 9.14	137.4 ± 8.81
Shikonin ^c^	2.92 ± 0.09	2.80 ± 0.26	2.47 ± 0.19	2.77 ± 0.29	10.25 ± 1.37	7.00 ± 0.89	11.77 ± 0.14
Colchicine ^c^	1.47 ± 0.07	1.81 ± 0.06	2.13 ± 0.13	2.94 ± 0.12	1.17 ± 0.09	8.43 ± 0.71	9.18 ± 0.62
CA-4 ^c^	0.27 ± 0.007	0.31 ± 0.02	0.11 ± 0.006	0.09 ± 0.008	0.23 ± 0.006	1.44 ± 0.011	0.36 ± 0.003

^a^ The data indicate the mean ± SD of at least three independent experiments. ^b^ Cytotoxicity in human normal cells. ^c^ Standard drugs for the study. ND means not detected.

**Table 21:** Evaluation of the impact of hybrids **83c**, **83f**, and **83i** on the inhibition of tubulin polymerization and colchicine binding to tubulin.

Compound	Inhibition of Tubulin Polymerization IC_50_ (µM) ^a^	Inhibition of Colchicine Binding (% Inhibition) ^a^	
		2 µM	4 µM
**83c**	0.98 ± 0.014	82.36 ± 0.88	92.42 ± 0.79
**83f**	4.13 ± 0.26	ND	ND
**83i**	2.37 ± 0.21	ND	ND
Colchicine ^b^	2.11 ± 0.32	ND	ND
CA-4 ^b^	1.12 ± 0.13	81.46 ± 0.94	92.96 ± 0.87

^a^ The data indicate the mean ± SD of at least three independent experiments. ^b^ Standard references for the study. ND means not detected.

**Table 22:** Antiproliferative activity of compounds **103a**–**l**, **103n**–**o**, and CA-4.

Compound	IC_50_ (nM) ^a^					
	HeLa	HT-29	Daoy	HL-60	SEM	Jurkat
**103a**	413 ± 40.3	591 ± 29	371 ± 19	339 ± 21	250 ± 28	385 ± 52
**103b**	5225 ± 312	6512 ± 263	3950 ± 236	4891 ± 445	4557 ± 369	2760 ± 156
**103c**	56.0 ± 19	62.9 ± 12.9	36.2 ± 6.9	36.2 ± 11	38.9 ± 12	29.2 ± 9.3
**103d**	2.9 ± 0.3	5.6 ± 0.9	0.30 ± 0.08	3.0 ± 0.2	4.1 ± 0.2	2.7 ± 0.6
**103e**	496 ± 39	477 ± 42	345 ± 36	233 ± 31	285 ± 26	333 ± 42
**103f**	2.8 ± 0.3	2.1 ± 0.2	0.005 ± 0.001	2.7 ± 0.2	0.31 ± 0.05	0.28 ± 0.08
**103g**	22.7 ± 1.8	17.7 ± 1.2	4.4 ± 0.9	41.7 ± 15.9	29.7 ± 8.9	27.2 ± 1.9
**103h**	1250 ± 98	910 ± 58	1670 ± 87	428 ± 36	385 ± 27	390 ± 39
**103i**	9670 ± 125	8680 ± 458	>10,000	7056 ± 659	3675 ± 298	4390 ± 154
**103j**	591 ± 26	682 ± 45	846 ± 28	413 ± 56	333 ± 45	299 ± 39
**103k**	4.5 ± 0.5	5.4 ± 0.3	2.5 ± 0.2	5.6 ± 0.9	3.5 ± 0.1	2.8 ± 0.6
**103l**	1.1 ± 0.2	0.6 ± 0.02	0.34 ± 0.1	3.5 ± 0.4	0.30 ± 0.03	0.33 ± 0.05
**103n**	591 ± 63	3420 ± 368	428 ± 45	285 ± 2.5	371 ± 1.5	299 ± 37
**103o**	635 ± 1.5	790 ± 98	494 ± 56	413 ± 3.8	259 ± 1.8	399 ± 58
CA-4 ^b^	4 ± 1	3100 ± 100	12.3 ± 0.09	1 ± 0.2	5 ± 0.1	0.8 ± 0.2

^a^ The data indicate the mean ± SD of at least three independent experiments. ^b^ The standard drug for the study.

**Table 23:** Evaluation of the impact of compounds **103c**–**d**, **103f**–**g**, **103k**–**l**, and CA-4 on the inhibition of tubulin polymerization and colchicine binding to tubulin.

Compound	Inhibition of Tubulin Polymerization IC_50_ (µM) ^a^	Inhibition of Colchicine Binding (% Inhibition) ^a^	
		5 µM	0.5 µM
**103c**	0.50 ± 0.09	73 ± 0.5	ND
**103d**	0.48 ± 0.05	99 ± 0.2	79 ± 1
**103f**	0.37 ± 0.02	99 ± 0.3	86 ± 0.8
**103g**	0.51 ± 0.1	91 ± 0.3	42 ± 0.4
**103k**	0.57 ± 0.04	92 ± 1	56 ± 2
**103l**	0.39 ± 0.04	97 ± 0.4	74 ± 0.1
CA-4 ^b^	0.54 ± 0.06	97 ± 0.8	82 ± 2

^a^ The data indicate the mean ± SD of at least three independent experiments. ^b^ The standard drug for the study. ND means not detected.

**Table 24:** Percentage inhibition of evodiamine derivatives **115** and **116** on MDA-MB-435 cell line.

Compound	% Inhibition ^a^		
	100 µM	10 µM	1 µM
**115a**	36.6 ± 2.7	10.6 ± 0.5	6.0 ± 0.4
**115b**	15.0 ± 1.9	11.9 ± 1.5	7.1 ± 1.2
**115c**	13.0 ± 0.7	9.2 ± 0.5	6.8 ± 2.4
**115d**	41.5 ± 2.6	19.2 ± 2.8	7.5 ± 1.0
**115e**	12.1 ± 0.7	5.5 ± 0.2	8.6 ± 0.7
**115f**	15.9 ± 0.9	7.3 ± 0.6	2.3 ± 0.1
**116a**	96.5 ± 9.6	61.2 ± 4.9	55.1 ± 3.0
**116b**	97.4 ± 8.3	66.5 ± 8.5	60.6 ± 12.8
**116c**	94.5 ± 6.2	83.1 ± 6.2	75.7 ± 8.2
**116d**	94.0 ± 6.1	64.8 ± 7.0	62.7 ± 9.3
**116e**	96.9 ± 9.8	59.5 ± 2.1	32.7 ± 4.6
**116f**	95.8 ± 4.1	84.3 ± 5.8	82.4 ± 7.4
Evodiamine ^b^	73.5 ± 5.5	53.3 ± 3.1	38.9 ± 3.1

^a^ The data indicate the mean ± SD of at least three independent experiments. ^b^ The standard drug for the study.

**Table 25:** Antiproliferative activity of evodiamine derivatives **116a**–**f**.

Compound	IC_50_ (µM) ^a^			
	MDA-MB-435	HCT116	A549	MDA-MB-231
**116a**	0.47 ± 0.03	1.26 ± 0.10	4.28 ± 0.30	0.86 ± 0.07
**116b**	1.95 ± 0.23	3.45 ± 0.24	6.79 ± 0.64	2.52 ± 0.33
**116c**	0.81 ± 0.06	1.71 ± 0.13	4.14 ± 0.33	1.16 ± 0.14
**116d**	1.14 ± 0.09	4.71 ± 0.32	10.49 ± 0.97	1.05 ± 0.09
**116e**	2.35 ± 0.18	6.09 ± 0.46	7.58 ± 0.67	3.73 ± 0.28
**116f**	0.42 ± 0.03	0.76 ± 0.07	5.24 ± 0.34	0.36 ± 0.02
Evodiamine ^b^	6.30 ± 0.54	32.67 ± 2.30	77.24 ± 6.33	13.30 ± 1.84
Camptothecin ^b^	0.31 ± 0.07	0.09 ± 0.008	0.10 ± 0.001	0.42 ± 0.03

^a^ The data indicate the mean ± SD of at least three independent experiments. ^b^ The standard drugs for the study.

**Table 26:** Antiproliferative activity of compounds **117a**–**r**.

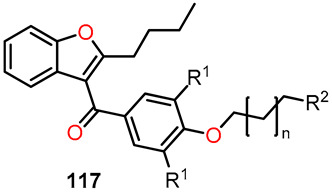								
Compound	R^1^	R^2^	*n*	10 µM of DMSO SIRT3	IC_50_ (µM) ^a^			
					HL60	U937	MCF-7	MDA-MB-231
**117a**	I	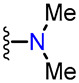	0	116.35 ± 1.34	8.22 ± 1.42	8.68 ± 0.48	39.37 ± 1.28	6.60 ± 0.27
**117b**	I	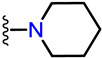	0	121.12 ± 2.76	>50	>50	12.78 ± 0.52	>50
**117c**	I	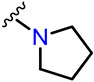	1	144.69 ± 0.73	2.80 ± 0.64	2.52 ± 2.32	12.46 ± 0.85	2.19 ± 0.16
**117d**	I	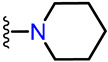	1	134.73 ± 0.93	11.46 ± 1.45	18.19 ± 1.42	37.94 ± 0.37	15.66 ± 0.95
**117e**	I	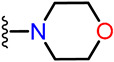	1	136.73 ± 1.24	>50	>50	8.05 ± 0.61	6.81 ± 1.69
**117f**	I	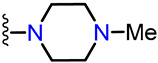	1	114.52 ± 0.71	>50	>50	5.37 ± 0.23	8.76 ± 1.25
**117g**	I	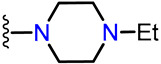	1	126.52 ± 2.19	28.55 ± 1.56	13.65 ± 0.98	39.47 ± 1.26	17.96 ± 0.94
**117h**	I	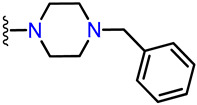	1	93.63 ± 1.97	>50	41.36 ± 1.30	28.67 ± 1.03	15.68 ± 1.52
**117i**	I	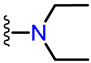	1	113.64 ± 0.17	16.02 ± 1.56	30.04 ± 2.45	21.54 ± 1.92	26.80 ± 0.87
**117j**	I	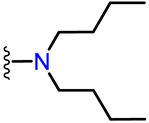	1	123.40 ± 1.50	24.32 ± 1.49	7.44 ± 0.34	18.52 ± 0.79	11.27 ± 0.58
**117k**	I	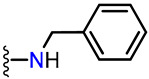	1	107.25 ± 1.53	4.40 ± 0.46	2.30 ± 1.57	8.05 ± 0.16	11.57 ± 0.83
**117l**	I	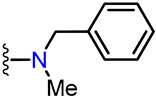	1	92.43 ± 3.64	8.51 ± 1.30	6.86 ± 1.40	8.02 ± 0.29	53.97 ± 1.89
**117m**	H	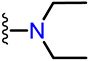	1	92.44 ± 1.53	2.77 ± 3.62	3.47 ± 0.56	5.26 ± 0.68	19.4 ± 0.78
**117n**	H	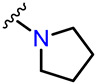	1	93.53 ± 3.14	6.38 ± 0.74	6.82 ± 0.16	3.73 ± 0.26	10.54 ± 0.88
**117o**	I	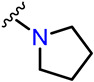	0	111.48 ± 1.83	23.15 ± 2.23	31.66 ± 1.86	>50	20.43 ± 2.06
**117p**	Cl	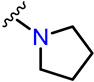	1	121.31 ± 2.92	11.34 ± 1.09	25.24 ± 0.95	27.66 ± 1.42	28.16 ± 1.53
**117q**	Br	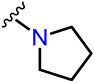	1	119.09 ± 1.06	38.27 ± 1.36	>50	31.34 ± 1.22	21.64 ± 1.53
**117r**	Me	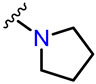	1	108.47 ± 1.95	41.33 ± 2.27	48.29 ± 1.98	15.58 ± 0.96	29.87 ± 0.91
ZINCO3830212 ^b^	I	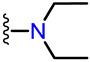	0	115.23 ± 2.20	5.27 ± 1.06	9.31 ± 1.45	>50	33.43 ± 2.84

^a^ The data indicate the mean ± SD of at least three independent experiments. ^b^ The reference compound for the study.

**Table 27:** Emax, EC_50_, and IC_50_ values of compound **117c**.

Compound	Emax ^a^	EC_50_ (µM)	IC_50_ (µM) ^a^ MDA-MB-231
**117c**	1.00 ± 0.07	0.21	2.19 ± 0.16
Resveratrol ^b^	0.25 ± 0.03	55.19	98.89 ± 2.16
Honokiol ^b^	0.91 ± 0.06	0.17	44.89 ± 1.86

^a^ The data indicate the mean ± SD of at least three independent experiments. ^b^ The reference compounds for the study.

**Table 28:** Synthesis of benzo[*b*]furan-based oxadiazole/triazole derivatives **120a**–**g** and **121a**–**h** under ultrasound/microwave irradiation.

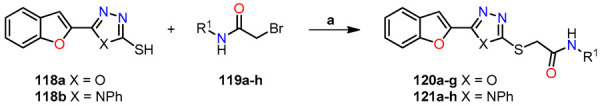			
Compound	R^1^	Yield (%)	
		Method A	Method B
**120a**	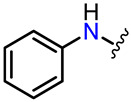	70	75
**120b**	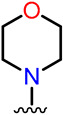	66	70
**120c**	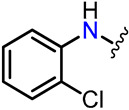	68	75
**120d**	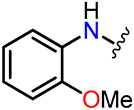	86	94
**120e**	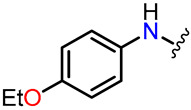	73	82
**120f**	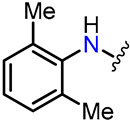	60	69
**120g**	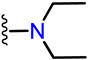	66	74
**121a**	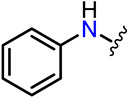	80	90
**121b**	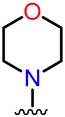	64	73
**121c**	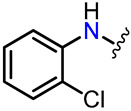	60	68
**121d**	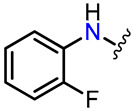	63	74
**121e**	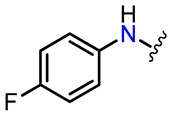	69	77
**121f**	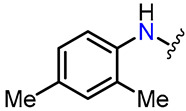	61	70
**121g**	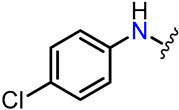	77	89
**121h**	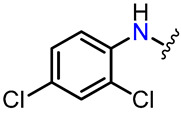	90	96

(**a**) Reagents and conditions: (i) method A: pyridine, CH3CN, 30 min, 40 °C, USI and (ii) method B: pyridine, DMF, 60–70 s, MWI.

**Table 29:** Hemolysis, thrombolysis, and antiproliferative studies of benzo[*b*]furan-based oxadiazole/triazole derivatives **120a**–**g** and **121a**–**h**.

Compound	% Hemolysis ^a^	% Thrombolysis ^a^	% Cell Viability ^a^ A549	IC_50_ (µM) ^a^ A549
**120a**	3.7 ± 0.008	56.8 ± 0.081	64.1 ± 1.72	ND
**120b**	22.12 ± 0.008	50.7 ± 0.081	45.99 ± 4.22	ND
**120c**	1.3 ± 0.008	52.8 ± 0.081	43.7 ± 0.94	ND
**120d**	5.02 ± 0.008	53.5 ± 0.081	27.49 ± 1.90	6.3 ± 0.7
**120e**	0.5 ± 0.008	52.4 ± 0.081	34.47 ± 2.19	17.9 ± 0.46
**120f**	0.74 ± 0.008	56.5 ± 0.081	43.67 ± 4.43	ND
**120g**	4.86 ± 0.047	48.3 ± 0.081	41.45 ± 4.10	ND
**121a**	9.6 ± 0.081	52.2 ± 0.081	49.8 ± 1.06	ND
**121b**	0.1 ± 0.004	52.5 ± 0.081	57.62 ± 4.94	ND
**121c**	2.15 ± 0.008	54 ± 0.081	44.52 ± 5.01	ND
**121d**	6.13 ± 0.047	56.2 ± 0.081	39.12 ± 2.21	ND
**121e**	3.11 ± 0.008	59.1 ± 0.008	44.72 ± 0.84	ND
**121f**	15.7 ± 0.081	61.4 ± 0.081	99.1 ± 5.04	ND
**121g**	23.4 ± 0.081	49.06 ± 0.047	36.26 ± 0.41	19.8 ± 0.54
**121h**	14.8 ± 0.081	48.1 ± 0.081	29.29 ± 3.98	10.9 ± 0.94
**ABTS**	95.9	86	ND	ND
**DMSO**	ND	ND	100 ± 0	ND
Crizotinib ^b^	ND	ND	28.22 ± 3.88	8.54 ± 0.84
Cisplatin ^b^	ND	ND	15.34 ± 2.98	3.88 ± 0.76

^a^ The data indicate the mean ± SD of at least three independent experiments. ^b^ The standard drugs for the study. ND means not determined.

**Table 30:** Isolation of alkaloids **127a**–**i** and their corresponding masses.

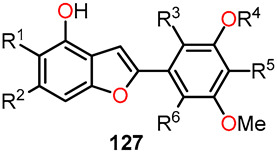							
Compound	R^1^	R^2^	R^3^	R^4^	R^5^	R^6^	Mass (mg)
**127a**	H	H	Me	H	Me	H	13.2
**127b**	H	H	Me	H	Me	Me	1.8
**127c**	H	H	Me	Me	Me	H	33.9
**127d**	H	OMe	Me	H	H	H	6.4
**127e**	H	OMe	H	Me	H	H	0.9
**127f**	H	H	Me	Me	Me	Me	0.6
**127g**	H	OMe	Me	Me	H	H	5.8
**127h**	Me	OMe	Me	H	H	H	2.0
**127i**	H	OMe	Me	Me	Me	H	12.8

**Table 31:** MIC values (µg/mL) of some alkaloids of **127**.

Compound	Gram-Negative Bacteria		Gram-Positive Bacteria		
	*E. coli*	*K. pneumoniae*	*S. aureus*	*MRSA*	*S. pyogenes*
**127a**	62.5	62.5	62.5	15.6	ND
**127c**	62.5	62.5	62.5	15.6	62.5
**127d**	125.0	62.5	31.3	31.3	62.5
**127g**	62.5	125.0	31.3	62.5	ND
**127i**	62.5	62.5	31.3	15.6	ND
Gentamicin ^a^	11.3	11.3	22.5	45.0	5.6

^a^ The standard drug for the study. ND means not determined.

**Table 32:** Synthesis of *bis*-chalcones **130a**–**g** via Claisen–Schmidt condensation using both conventional and microwave heating methods.

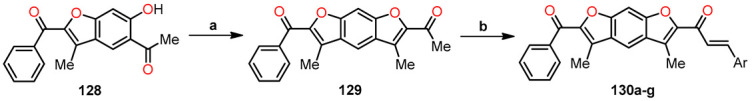					
Compound	Ar	Method A		Method B	
		Time (h)	Yield (%)	Time (min)	Yield (%)
**130a**	Ph	6	68	5	92
**130b**	2-ClPh	7	64	5	90
**130c**	4-MeOPh	7	65	4	94
**130d**	2-Furyl	6	64	4	89
**130e**	*α*-Napthyl	8	66	5	92
**130f**	1,3-diPhenyl-1*H*-pyrazol-4-yl	8	56	5	87
**130g**	1-Phenyl-3-(4-bromophenyl)-1*H*-pyrazol-4-yl	8	53	5	93

(*i*) Method A: (**a**) K_2_CO_3_, ClCH_2_COCH_3_, CH_3_COCH_3_, reflux, 8 h; (**b**) NaOH, ArCHO, EtOH, reflux, 6–8 h, 53–68%, and (*ii*) Method B: (**a**) K_2_CO_3_, ClCH_2_COCH_3_, 120 °C, 4 min, MWI; (**b**) NaOH, ArCHO, 90 °C, 4–5 min, MWI, 87–94%.

**Table 33:** Inhibition zones (in mm) of *bis*-chalcones **130a**–**g**.

Compound	Gram-Negative Bacteria		Gram-Positive Bacteria	
	*E. coli*	*P. aeruginosa*	*B. subtilis*	*S. aureus*
**130a**	9	10	15	10
**130b**	10	9	16	9
**130c**	7	7	10	8
**130d**	8	6	10	8
**130e**	7	8	9	7
**130f**	10	9	16	10
**130g**	11	10	17	10
Chloramphenicol ^a^	13	ND	ND	ND
Carbenicillin ^a^	ND	13	ND	ND
Streptomycin ^a^	ND	ND	22	ND
Tetracycline ^a^	ND	ND	ND	15

^a^ The standard drugs for the study. ND means not determined.

**Table 34:** Structures of benzo[*b*]furancarboxylic acids **131b**–**g**, and benzo[*b*]furancarboxylates **133b**–**d**, **133f**, **134c**–**d**, and **134f**–**g**.

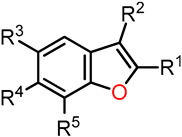					
Compound	R^1^	R^2^	R^3^	R^4^	R^5^
**131b**	COOH	Me	H	OH	Ac
**131c**	COOH	Me	OMe	OH	Ac
**131d**	COOH	Me	H	OH	4-Methoxycinnamaldehyde
**131e**	COOH	Me	H	OMe	Ac
**131f**	COOH	H	Br	OMe	OH
**131g**	COOH	H	H	H	OMe
**133b**	COOMe	Me	H	O(CH_2_)_2_NEt_2_	Ac
**133c**	COOMe	Me	OMe	O(CH_2_)_2_NEt_2_	Ac
**133d**	COOMe	Me	H	O(CH_2_)_2_NEt_2_	4-Methoxycinnamaldehyde
**133f**	COOMe	H	Br	OMe	O(CH_2_)_2_NEt_2_
**134c**	COO(CH_2_)_2_NEt_2_	Me	OMe	OH	Ac
**134d**	COO(CH_2_)_2_NEt_2_	Me	H	OH	4-Methoxycinnamaldehyde
**134f**	COO(CH_2_)_2_NEt_2_	H	Br	OMe	OMe
**134g**	COO(CH_2_)_2_NEt_2_	H	H	H	OMe

**Table 35:** Antimicrobial activity of hydrochlorides of methyl benzo[*b*]furancarboxylates **133**, **134**, and **135**.

Compound	MIC (µmol/cm^3^)							
	*M. luteus*	*B. cereus*	*B. subtilis*	*S. epidermidis*	*S. aureus*	*E. hirae*	*E. coli*	*P. aeruginosa*
**133a.HCl**	0.05	0.05	0.05	0.05	0.05	0.39	6.51	13.02
**133b.HCl**	0.75	1.49	1.49	1.49	3.11	3.11	12.44	ND
**133c.HCl**	0.05	0.36	0.18	0.18	0.18	0.36	6.04	ND
**133d.HCl**	0.04	0.30	0.04	0.04	0.15	0.60	ND	ND
**133f.HCl**	0.003	0.012	0.012	0.012	0.012	0.012	1.50	3.12
**134a.HCl**	0.01	0.10	0.01	ND	0.41	ND	ND	ND
**134c.HCl**	0.01	0.05	0.09	0.19	0.09	0.75	ND	ND
**134d.HCl**	0.01	0.04	0.04	0.01	0.04	0.08	ND	ND
**134f.HCl**	0.09	0.71	0.35	0.09	0.18	ND	ND	ND
**134g.HCl**	15.28	15.28	15.28	15.28	15.28	>30.56	>30.56	30.56
**135a.HCl**	0.04	0.04	0.04	0.04	0.07	0.30	0.59	ND

ND means not determined.

**Table 36:** Zone of inhibition of benzo[*b*]furan derivatives **139a**–**i**.

Compound	Concentration (mg/mL)	Zone of Inhibition (mm) ^a^				
		*P. syringae*	*S. typhi*	*B. subtilis*	*K. pneumoniae*	*E. coli*
**139a**	1.0	9 ± 0.3	10 ± 0.2	12 ± 0.1	13 ± 0.1	10 ± 0.3
	0.5	7 ± 0.1	9 ± 0.3	10 ± 0.3	11 ± 0.1	8 ± 0.1
**139b**	1.0	7 ± 0.2	6 ± 0.1	7 ± 0.2	7 ± 0.3	6 ± 0.2
	0.5	6 ± 0.3	5 ± 0.2	5 ± 0.2	5 ± 0.1	4 ± 0.2
**139c**	1.0	12 ± 0.2	12 ± 0.1	15 ± 0.2	16 ± 0.3	13 ± 0.2
	0.5	10 ± 0.3	10 ± 0.2	13 ± 0.1	14 ± 0.1	11 ± 0.3
**139d**	1.0	10 ± 0.2	10 ± 0.1	10 ± 0.2	11 ± 0.3	10 ± 0.2
	0.5	8 ± 0.3	7 ± 0.2	8 ± 0.1	10 ± 0.1	8 ± 0.3
**139e**	1.0	11 ± 0.2	11 ± 0.1	12 ± 0.2	13 ± 0.3	11 ± 0.2
	0.5	8 ± 0.3	8 ± 0.2	10 ± 0.1	12 ± 0.1	9 ± 0.3
**139f**	1.0	9 ± 0.2	8 ± 0.1	9 ± 0.2	8 ± 0.3	7 ± 0.2
	0.5	6 ± 0.3	6 ± 0.1	6 ± 0.2	ND	6 ± 0.3
**139g**	1.0	10 ± 0.2	9 ± 0.1	10 ± 0.2	9 ± 0.3	9 ± 0.2
	0.5	8 ± 0.3	8 ± 0.2	7 ± 0.1	9 ± 0.1	7 ± 0.3
**139h**	1.0	11 ± 0.2	12 ± 0.1	13 ± 0.2	14 ± 0.3	12 ± 0.2
	0.5	8 ± 0.3	9 ± 0.2	11 ± 0.1	13 ± 0.1	9 ± 0.3
**139i**	1.0	8 ± 0.2	7 ± 0.1	8 ± 0.2	8 ± 0.3	6 ± 0.2
	0.5	7 ± 0.3	6 ± 0.2	6 ± 0.2	5 ± 0.1	4 ± 0.3
Streptomycin ^b^	1.0	15 ± 0.2	16 ± 0.1	18 ± 0.2	19 ± 0.3	16 ± 0.2
	0.5	13 ± 0.3	15 ± 0.2	16 ± 0.1	17 ± 0.1	14 ± 0.3

^a^ The data indicate the mean ± SD of at least three independent experiments. ^b^ The standard drug for the study. ND means not determined.

**Table 37:** Minimum inhibitory concentration (MIC) of benzo[*b*]furan derivatives **139a**–**i**.

Compound	MIC (µg/mL)				
	*P. syringae*	*S. typhi*	*B. subtilis*	*K. pneumoniae*	*E. coli*
**139a**	15.50	15.80	16.50	16.50	16.00
**139b**	120.35	125.40	195.50	125.55	185.30
**139c**	14.80	15.25	15.75	16.00	15.75
**139d**	60.25	55.75	ND	32.85	38.65
**139e**	30.25	25.25	20.25	ND	40.25
**139f**	ND	70.35	85.25	75.35	85.35
**139g**	105.35	110.35	95.25	155.35	185.35
**139h**	16.25	16.75	17.50	17.25	17.50
**139i**	125.35	115.35	195.25	120.35	175.35
Streptomycin ^a^	14.50	14.50	15.50	15.25	15.25

^a^ The standard drug for the study. ND means not determined.

**Table 38:** Antibacterial activity of naftifine analogs **140**, **141**, and **142a**–**c** against *S. aureus* Newman.

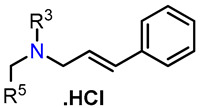			
Compound	R^3^	R^5^	*S. aureus* Newman IC_50_ (nM) ^a^
**140**	Me	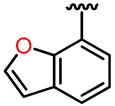	247.3 ± 18.8
**141**	Me	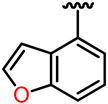	758.7 ± 24.3
**142a**	H	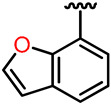	>1000
**142b**	Et	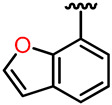	>1000
**142c**	*i*-Propyl	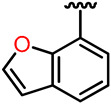	>1000

^a^ The data indicate the mean ± SD of at least three independent experiments.

**Table 39:** Antibacterial activity of naftifine analogs **144a**–**t** against *S. aureus* Newman.

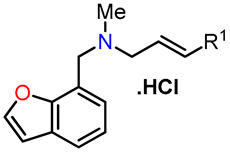					
Compound	R^1^	*S. aureus* Newman IC_50_ (nM) ^a^	Compound	R^1^	*S. aureus* Newman IC_50_ (nM) ^a^
**144a**	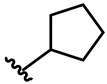	>1000	**144k**	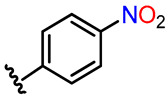	71.1 ± 2.5
**144b**	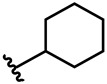	>1000	**144l**	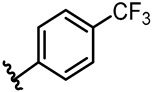	4.0 ± 0.2
**144c**	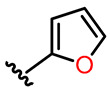	>1000	**144m**	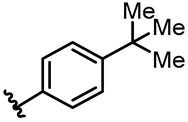	46.7 ± 3.2
**144d**	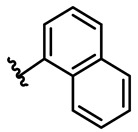	887.7 ± 60.0	**144n**	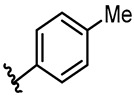	35.2 ± 3.8
**144e**	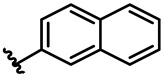	17.1 ± 1.5	**144o**	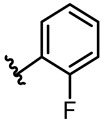	288.3 ± 18.2
**144f**	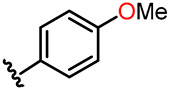	11.2 ± 1.1	**144p**	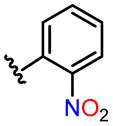	>1000
**144g**	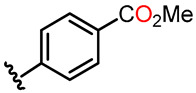	16.9 ± 1.5	**144q**	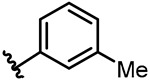	>1000
**144h**	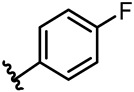	31.2 ± 2.1	**144r**	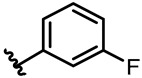	513.0 ± 11.1
**144i**	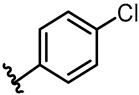	6.3 ± 0.1	**144s**	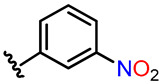	>1000
**144j**	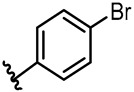	6.7 ± 0.1	**144t**	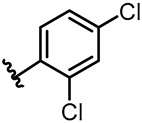	8.3 ± 0.3

^a^ The data indicate the mean ± SD of at least three independent experiments.

**Table 40:** CrtN enzymatic inhibition and water solubility of naftifine analogs **144**.

Compound	CrtN IC_50_ (nM) ^a^	*S. aureus* Newman IC_50_ (nM) ^a^	Solubility (mg/mL)
**144f**	683.7 ± 68.1	11.08 ± 1.06	19.7
**144i**	219.0 ± 16.8	6.20 ± 1.02	7.4
**144j**	355.1 ± 26.1	6.44 ± 1.02	3.9
**144l**	338.8 ± 28.3	3.93 ± 1.02	10.0
**144t**	740.2 ± 55.6	8.21 ± 1.03	7.2
NFT ^b^	8830.0 ± 109.1	296.0 ± 12.2	6.1

^a^ The data indicate the mean ± SD of at least three independent experiments. ^b^ The standard drug for the study.

**Table 41:** Evaluation of DNA binding ability for PDB derivatives **169**, **171**, and **172**.

Compound	ΔT_m_ (°C) at 1 µM ^a^	
	Sequence F1	Sequence F2
**169**	20.8 ± 0.2	9.5 ± 0.5
**171**	0.0 ± 0.2	0.4 ± 0.1
**172**	0.1 ± 0.1	0.5 ± 0.3
Netropsin ^b^	13.8 ± 0.3	11.0 ± 0.2
Ciprofloxacin ^b^	0.0 ± 0.4	0.2 ± 0.3

^a^ The data indicate the mean ± SD of three independent experiments. ^b^ The standard drugs for the study.

**Table 42:** Antibacterial activity of PBD derivatives **169**, **171**, and **172**.

Compound	Gram-Positive Strains MIC (µg/mL)					
	VRE		VSE	MRSA		MSSA
	NCTC 12201	NCTC 12204	NCTC 775	EMRSA 15	EMRSA 16	ATCC 9144
**169**	≤0.125	≤0.125	≤0.125	≤0.125	≤0.125	≤0.125
**171**	2	2	16	>32	>32	16
**172**	0.5	1	4	2	4	2
Ciprofloxacin ^a^	0.5	2	1	32	32	≤0.125

^a^ The standard drug for the study.

**Table 43:** IC_50_ values of benzo[*b*]furan cyanide derivatives in Sortase A inhibition.

Compound	R^1^	R^2^	IC_50_ (μM) ^a^
**175a**	H	H	8.8 ± 1.4
**175b**	H	4-Me	14.1 ± 0.9
**175c**	H	3,4-diMe	17.5 ± 2.2
**175d**	H	2,4,6-triMe	32.2 ± 1.6
**175e**	H	4-Et	9.4 ± 0.9
**175f**	H	4-OMe	11.9 ± 0.7
**175g**	H	3,4-diOMe	9.6 ± 0.3
**175h**	H	2,5-diOMe	21.8 ± 1.4
**175i**	H	4-Cl	9.8 ± 1.5
**175j**	H	4-Br	19.1 ± 0.6
**175k**	H	4-F	19.7 ± 1.0
**175l**	H	4-CF_3_	12.2 ± 1.8
**175m**	H	2-COOH	9.7 ± 0.3
**175n**	H	4-OBn	6.0 ± 0.8
**175o**	H	3,4-diOBn	3.3 ± 0.3
**175p**	7-OMe	H	11.9 ± 1.8
**175q**	7-OMe	4-Me	10.1 ± 0.7
**175r**	7-OMe	3,4-diMe	>100
**175s**	7-OMe	4-Et	48.6 ± 5.9
**175t**	7-OMe	4-OMe	12.1 ± 0.1
**175u**	7-OMe	3,4-diOMe	14.5 ± 1.1
**175v**	7-OMe	2,5-diOMe	52.8 ± 7.9
**175w**	7-OMe	4-Cl	5.9 ± 1.5
**175x**	7-OMe	4-Br	15.5 ± 1.3
**175y**	7-OMe	2-COOH	28.4 ± 0.4
**175z**	5-Cl	H	29.9 ± 1.2
**175a_2_**	5-Cl	4-Me	47.1 ± 7.3
**175b_2_**	5-Cl	3,4-diMe	>100
**175c_2_**	5-Cl	2,4,6-triMe	37.9 ± 1.5
**175d_2_**	5-Cl	4-Et	13.8 ± 0.6
**175e_2_**	5-Cl	4-OMe	64.1 ± 4.4
**175f_2_**	5-Cl	3,4-diOMe	49.1 ± 4.4
**175g_2_**	5-Cl	2,5-diOMe	15.5 ± 0.9
**175h_2_**	5-Cl	4-Cl	6.8 ± 0.1
**175i_2_**	5-Cl	4-Br	16.4 ± 0.8
**176**	--	--	15.2 ± 1.7
**177a**	H	4-OBn	15.0 ± 0.9
**178a**	H	4-OH	7.9 ± 0.7
**178b**	H	3,4-diOH	13.7 ± 1.2
pHMB ^b^	-	-	130.0 ± 4.3

^a^ The data indicate the mean ± SD of at least three independent experiments. ^b^ The standard reference for the study.

**Table 44:** Inhibition of biofilm formation and bacterial invasion using four cyano derivatives of benzo[*b*]furan.

Compound	Biofilm Inhibition IC_50_ (μM) ^a^	% Reduction ^a^
**175a**	19.7 ± 0.3	17.9 ± 3.2
**175o**	2.1 ± 0.1	24.0 ± 6.4
**175h_2_**	54.2 ± 0.5	4.0 ± 1.9
**178a**	14.3 ± 0.4	2.8 ± 1.6

^a^ The data indicate the mean ± SD of at least three independent experiments.

**Table 45:** Minimum inhibitory concentration (MIC) values in µg/mL for alkaloids against two fungal strains.

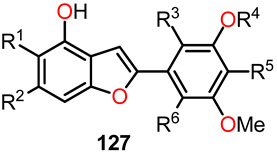		
Compound	*C. albicans*	*C. neoformans*
**127a**	31.3	7.8
**127c**	31.3	7.8
**127d**	31.3	31.3
**127g**	15.6	7.8
**127i**	15.6	15.6
Amphotericin B ^a^	15.6	3.9

^a^ The standard drug for the study.

**Table 46:** Antifungal activity of hydrochlorides of methyl benzo[*b*]furan carboxylates against five fungal strains.

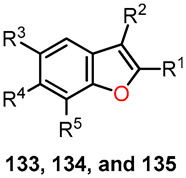					
	MIC (µmol/cm^3^)				
Compound	*C. albicans*	*C. parapsilosis*	*S. cerevisiae*	*Z. rouxii*	*A. brasiliensis*
**133a.HCl**	0.39	0.39	ND	0.39	0.78
**133b.HCl**	1.49	1.49	ND	ND	1.49
**133c.HCl**	0.36	0.72	ND	0.36	0.72
**133d.HCl**	4.98	0.2987	ND	ND	1.19
**133f.HCl**	0.09	0.094	0.187	0.023	0.094
**134a.HCl**	0.41	ND	1.62	ND	ND
**134c.HCl**	ND	ND	3.13	ND	ND
**134d.HCl**	0.08	0.15	0.08	0.04	ND
**134f.HCl**	ND	ND	1.42	5.91	ND
**134g.HCl**	>30.56	>30.56	15.28	>30.56	15.28
**135a.HCl**	4.96	4.96	4.96	0.59	4.96

ND means not determined.

**Table 47:** Antifungal activity of compounds **139a**–**i** against various fungal strains.

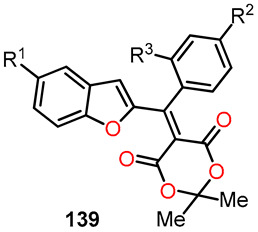				
	MIC (µg/mL)			
Compound	*C. albicans*	*A. flavus*	*M. griseus*	*A. terus*
**139a**	12.75	12.70	6.35	6.50
**139b**	35.50	ND	22.50	25.55
**139c**	12.60	12.60	6.30	6.35
**139d**	15.80	16.85	9.75	7.55
**139e**	13.70	12.85	8.35	8.55
**139f**	23.50	21.65	18.50	21.55
**139g**	31.45	ND	24.70	24.75
**139h**	12.80	12.75	6.45	6.40
**139i**	32.80	35.75	20.50	28.50
Fluconazole ^a^	12.50	12.50	6.25	6.25

^a^ The standard drug for the study. ND means not determined.

**Table 48:** Antifungal activity of the NFT analogue **144l**.

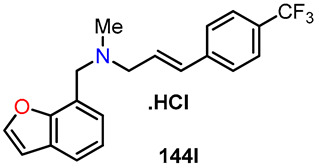			
	MIC (µg/mL)		
Compound	*T. rubrum*	*M. gypseum*	*T. barbae*
**144l**	16	32	>64
NFT ^a^	0.125	0.25	0.125
Fluconazole ^a^	1	8	2
Voriconazole ^a^	0.03125	0.25	0.03125
Ketoconazole ^a^	0.5	2	0.0625

^a^ The standard drugs for the study.

## Data Availability

Data sharing is not applicable.
